# DNA extraction and amplicon production strategies deeply inf luence the outcome of gut mycobiome studies

**DOI:** 10.1038/s41598-019-44974-x

**Published:** 2019-06-27

**Authors:** Alessandra Frau, John G. Kenny, Luca Lenzi, Barry J. Campbell, Umer Z. Ijaz, Carrie A. Duckworth, Michael D. Burkitt, Neil Hall, Jim Anson, Alistair C. Darby, Christopher S. J. Probert

**Affiliations:** 1 Gastroenterology Research Unit, Department of Cellular & Molecular Physiology, Institute of Translational Medicine, University of Liverpool, Ashton Street, Liverpool, L69 3GE UK; 2 Centre for Genomic Research (CGR), University of Liverpool, Crown Street, Liverpool, L69 7ZB UK; 3 School of Engineering, University of Glasgow, Oakfield Avenue, Glasgow, G12 8LT UK; 4 Division of Diabetes, Endocrinology and Gastroenterology, University of Manchester, Dover Street, Manchester, M13 9PT UK; 5 Earlham Institute, Colney Ln, Norwich, NR4 7UZ UK; 6 Liverpool Clinical Laboratories Directorate of Infection and Immunity, Royal Liverpool and Broadgreen University Hospitals NHS Trust, Prescot Street, Liverpool, L7 8XP UK; 7 Teagasc Food Research Centre, Moorepark, Cork Ireland

**Keywords:** Gastroenterology, Microbiome

## Abstract

Microbial ecology studies are often performed through extraction of metagenomic DNA followed by amplification and sequencing of a marker. It is known that each step may bias the results. These biases have been explored for the study of bacterial communities, but rarely for fungi. Our aim was therefore to evaluate methods for the study of the gut mycobiome. We first evaluated DNA extraction methods in fungal cultures relevant to the gut. Afterwards, to assess how these methods would behave with an actual sample, stool from a donor was spiked with cells from the same cultures. We found that different extraction kits favour some species and bias against others. In terms of amplicon sequencing, we evaluated five primer sets, two for ITS2 and one for ITS1, 18S and 28S rRNA. Results showed that 18S rRNA outperformed the other markers: it was able to amplify all the species in the mock community and to discriminate among them. ITS primers showed both amplification and sequencing biases, the latter related to the variable length of the product. We identified several biases in the characterisation of the gut mycobiome and showed how crucial it is to be aware of these before drawing conclusions from the results of these studies.

## Introduction

The microbiota inhabiting the gut is mainly made of prokaryotes, but micro-eukaryotes are also present, including fungi. Recently, the gut mycobiome in health and disease has been explored and its role in some chronic diseases, such as inflammatory bowel disease (IBD), has been proposed^1–4^. As has been the case for prokaryotes, the analysis of the gut mycobiome has mainly been based upon amplicon sequencing. However, unlike the techniques used to evaluate structure and diversity of bacterial communities, those applied to the analysis of the mycobiome have not, as yet, been thoroughly validated. A call has been made to address this issue^5^ but this has only been given attention by a few recent studies^6,7^.

Amplicon sequencing requires the extraction of DNA from a diverse and complex mixture of microorganisms whose identity is determined at the end of the experiment. Therefore, DNA extraction needs to be effective for a wide range of taxa; efficient extraction is crucial and its influence on the outcome is significant^8–10^. As for prokaryotic cells, the main challenge during the extraction of DNA from fungi is the lysis of the cell wall. In fungi, the cell wall is made of several polysaccharides, proteins and glycoproteins. Polysaccharides make up around 80% of the wall, while proteins comprise 20%. Lipid and wax can also be found in low concentration and these are mainly involved in preventing desiccation^11^. Generally, there are three layers: the outermost layer is composed of glycoproteins with glucan or mannan polysaccharides, followed by a layer of *β*-1,3 glucans and the innermost layer is made from chitin^11^. The structure of the cell wall varies among different taxa: ratios of the components listed above can change and their composition may vary^11^. The cell wall also varies according to the cell status within the same species (e.g. spore vs hyphae)^12^. This variation influences the extraction efficiency of a given DNA extraction procedure in various species^13,14^. Cell lysis can be achieved by chemical treatment (with detergents, acids or alkaline agents), enzymatic degradation of cell wall components (with enzymes such as proteinase K, lyticase and chitinase) or using mechanical/physical approaches (e.g. sonication, bead beating, high temperature etc.)^15^. Often, a combination of these may be applied, especially if the goal is to broaden the range of species extracted. In early studies describing the gut mycobiome, three main approaches have been applied to extract DNA from faecal samples: (i) published protocols, which have often included bead beating^4,16,17^, (ii) commercial kits used as per manufacturer defined protocols^18–23^ and (iii) commercial kit protocols which have been modified with the addition of extra initial steps, such as bead beating and/or enzymatic degradation, to improve efficiency of cell lysis^24–30^.

To date, no unique genetic biomarker for the study of fungal communities has been identified. The entire internal transcribed spacer (ITS) has been proposed as the “*universal barcode marker for fungi*”^31^. However, whether the ITS1 or ITS2 region of fungal ribosomal DNA (rDNA) is better for metabarcoding complex fungal communities is unclear: ITS1 was thought to be more variable and hence should allow better distinction among species than ITS2^32^, but the opposite has been shown^33,34^. ITS allows classification at species level, but the high variability in sequence and in length does not enable phylogenetic analysis or classification at high taxonomic ranks^35,36^. Genes encoding for the large (LSU) and small (SSU) rRNA subunit (28S rRNA and 18S rRNA) have also been considered as biomarkers for fungi^31^. These are more conserved, of regular length and can be used to make phylogenetic analysis^35^. However, the variability is considered too low to enable classification at species level, especially for 18S rRNA^31^. Higher resolution may be possible with 28S rRNA^31^, comparable to ITS^37,38^, although even this has been questioned^34^. A negative aspect of using LSU is that the reference sequence database is limited^6^. In order to achieve classification at species level and to enable phylogenetic analysis, the use of two markers, one from ITS and another from a region of the large or small rRNA subunit, could be considered; this would also allow us to understand amplification biases between these approaches^39^. The choice of primer set is also crucial, as it influences the community profile markedly^5,34^. It has been shown that ITS1F^40^ and ITS2^41^ primers, commonly used to characterise the gut mycobiome^3,28–30^, have mismatches to species relevant to the human mycobiome, including yeasts such as Saccharomycetes (ITS1F) and Basidiomycetes (ITS2) (i.e. *Malassezia* and *Cryptococcus*)^5^. Therefore, validation is needed before they are used to characterise metagenomes from the human body. In this context, it is clear that the choice of a suitable primer set is complicated. Because different markers and/or primers seem to bias against specific taxonomic groups, the environment analysed and its likely mycobiome must be taken into account when designing the experiment^34^.

This study has two main aims. First, we wanted to understand if DNA extraction kits specific for stool and developed for the extraction of bacterial metagenomes were also suitable for the extraction of fungal DNA. To do this, we first tested the PSP®Spin Stool DNA kit (Stratec) on seven pure fungal strains (*Candida albicans*, *Candida tropicalis*, *Saccharomyces cerevisiae*, *Cryptococcus neoformans*, *Malassezia furfur*, *Aspergillus fumigatus* and *Penicillium chrysogenum*), each of which has been detected in the gut in three or more studies^1,19,23,28^. The PSP®Spin Stool DNA kit was used as per manufacturer’s protocol and compared with a modification of the same, i.e. inclusion of an extra initial bead beating step, to determine whether this facilitated an increase in fungal DNA extraction. Secondly, the QIAamp®Fast DNA Stool Mini Kit (Qiagen) was tested on pure cultures of two of the seven fungal strains (*C*. *albicans* and *A*. *fumigatus*) with the goal of comparing two different DNA extraction kits. Extractions were compared by gene quantification of 18S rRNA^42^. Following this, and to evaluate the outcomes of the extraction on fungal cells in actual stool matrix, a human faecal sample from a healthy donor was spiked with cells from the seven fungal species listed above, and the extraction methods used for the pure cultures applied. These tests were also made on the non-spiked sample in order to exclude and evaluate the community already present in the sample as a background matrix for all other experiments. The outcomes were evaluated by 18S rRNA gene quantification^42^ and amplicon sequencing on the Illumina HiSeq platform. Finally, to test the performance of the extraction kits in a real scenario, 24 stool samples from donors were extracted with the two kits under evaluation (i.e. the PSP kit with additional bead beating and the Qiagen kit) and the fungal load (18S rRNA qPCR^42^) was measured. The second aim was to evaluate five primer sets to assess their performance and eventual biases on the characterisation of the gut mycobiome. The markers evaluated were 18S and 28S rRNA, ITS1 and ITS2. For ITS2, two primer sets were evaluated. A mock community made of the seven fungal species listed above and the templates obtained with the multiple extractions of the spiked/non-spiked stool sample (from a healthy donor) were used as a template for amplicon production and sequencing. To further test this in a real scenario, two primer sets were used to amplify and sequence stool DNA from 24 donors.

## Results

### DNA extraction from pure fungal cultures

**Figure 1:**
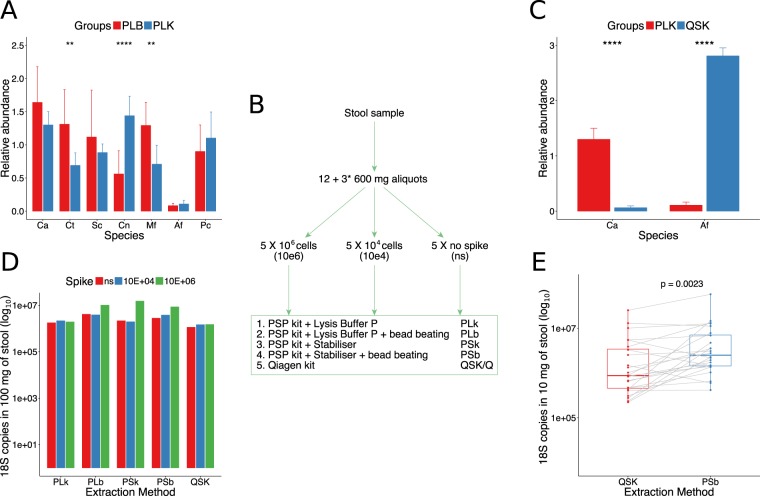
18S rRNA qPCR results (**A**,**C**,**D**,**E**) and flowchart (**B**) showing the aliquoting, spiking and extraction of the stool sample. This was aliquoted (line 2 of the flowchart), then a third of the aliquots was spiked with 10^6^ cells of each species of the mock community, a third was spiked with 10^4^ cells and the final third was analysed as it was (non-spiked). These were then extracted using 5 different methods (box). (**A**) Comparison of the PSP kit performance with extra bead beating (PLb) and without (PLk). (**C**) Comparison of the PSP kit (PLk) performance versus the Qiagen kit (QSK). (**A**,**C**) The relative abundance (DNA concentration) was normalised to the mean of all values. A Welch t-test was used to compare the means of the two extraction methods for each species; where results were found to be significant, this is shown in the figure (ns: p > 0.05; *p <= 0.05; **p <= 0.01; ***p <= 0.001; ****p <= 0.0001). The error bars denote standard deviation of the mean (SD). Fungal strains: Ca = *C*. *albicans*, Ct = *C*. *tropicalis*, Sc = *S*. *cerevisiae*, Cn = *C*. *neoformans*, Mf = *M*. *furfur*, Af = *A*. *fumigatus*, Pc = *P*. *crysogenum*. (**D**) Bar chart showing the number of 18S rRNA copies in 100 mg of spiked/non-spiked stools. The samples are grouped according to the extraction method, as shown in the flowchart. The colours indicate the spiking (ns = non-spiked, 10^4^ and 10^6^ cells). (**E**) Pair-wise comparison of 18S rRNA gene copies (method = wilcox.test) in 10 mg of stool from donors (n = 24) extracted with PSb and QSK kits. A list of sample names with related descriptions can be found in Table 1. *Sample preparation for the QSK extraction was done separately following the first aliquoting.

The approach and outcome of the extraction of DNA from fungal isolates are shown in Table 1 and Fig. 1 respectively. The addition of an initial 6-minute bead beating step prior to extraction using the PSP kit improved the yield of DNA from two of the isolates: *C*. *tropicalis* and *M*. *furfur*. However, the yield of *C*. *neoformans* DNA was reduced following bead beating (Fig. 1A). Comparing the PSP kit and the Qiagen kit (Fig. 1C) showed these to have significant and opposite results. The first improved the extraction of *C*. *albicans*, while the second greatly improved the extraction of *A*. *fumigatus*.

**Table 1:** Names of samples as used in Figs 1–5.

*Name*	*Extraction method*	
Pure cultures and stool samples extractions		
PLk	PSP kit + Lysis Buffer P	
PLb	PSP kit + Lysis Buffer P + bead beating	
PSk	PSP kit + Stabilizer	
PSb	PSP kit + Stabilizer + bead beating	
QSK/Q	Qiagen Kit	
***Name***	***Extraction method***	***Spike***
**Stool sample extractions and spike**		
PL10e6K	PSP kit + Lysis Buffer P	10^6^ cells
PL10e4K		10^4^ cells
PLnsK		Non-spiked
PL10e6b	PSP kit + Lysis Buffer P + bead beating	10^6^ cells
PL10e4b		10^4^ cells
PLnsb		Non-spiked
PS10e6K	PSP kit + Stabilizer	10^6^ cells
PS10e4K		10^4^ cells
PSnsK		Non-spiked
PS10e6b	PSP kit + Stabilizer + bead beating	10^6^ cells
PS10e4b		10^4^ cells
PSnsb		Non-spiked
Q10e6	Qiagen Kit	10^6^ cells
Q10e4		10^4^ cells
Qns		Non-spiked
***Name***	***Preparation***	
**Mock Community**		
PreMC	Genomic DNA from each fungal isolate was pooled then amplified (when replicates were processed, these are numbered 1 and 2)	
POSTMC	DNA was amplified separately for each species, then pooled before sequencing	
***Name***	***Sample type***	
**Donors**		
D	Stool samples from donors collected in the context of the SysMedIBD project

### DNA extraction from stool

**Figure 2:**
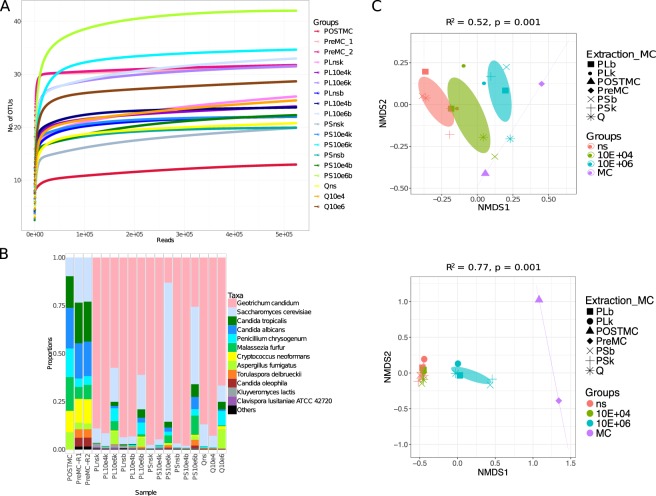
Use of the 18S rRNA primer set to define fungal diversity in stool (n = 15) and mock community (n = 3). (**A**) Rarefaction curves showing the number of OTUs versus the number of reads per sample. (**B**) Taxa summary showing the relative abundance of species, with each bar representing a sample. (**C**) Ordination of samples according to their community calculated through Non-Metric Distance Scaling (NMDS) was produced using unweighted UniFrac (top) and Bray-Curtis (bottom) distances. The ellipses were drawn at the 95% confidence interval of standard error and the mean value of the groups. PERMANOVA was used to assess if the clustering was significant. The *R*^2^ refers to the percentage of variability explained by the groups in terms of microbial community structure. A list of sample names, each with a related description, can be found in Table 1.

A flowchart summarising the aliquoting, spiking and extractions performed in stool is shown in Fig. 1B. In general, the methods behaved similarly, each retrieving approximately 10^6^ copies (Fig. 1D). However, the PSP kit did recover more fungal DNA than the Qiagen kit (Fig. 1E). As expected, the stool samples that were spiked with each of the seven fungal strains contained fungal DNA. Non-spiked samples (ns) also showed a conspicuous number of copies (from 1 × 10^6^ up to 5 × 10^6^ gene copies) that masked the spike with 10^4^ cells, especially if we consider that sequences assigned to the pure cultures were found in very low abundance in these samples (Fig. 2B).

Sequencing results of spiked stool samples are shown in Fig. 2. The taxa summary (Fig. 2B) shows that extraction with different methods generated different taxonomic profiles. The difference between the PSP kit with stabilizer and bead beating (PSb) and the Qiagen kit for the spike with 10^6^ cells was striking. The PSb method showed a higher relative abundance of *C*. *albicans* and less *A*. *fumigatus*, whereas the Qiagen kit showed the opposite. The reduced *C*. *albicans* and increased *A*. *fumigatus* in Q10e6 confirm the qPCR results (Fig. 1C). However, the sequencing results of PLk, the third extraction method tested, did not entirely confirm the qPCR results carried out in pure cultures. In PL10e6k there was a reduction of *A*. *fumigatus* compared to Q10e6, but it was not coupled with an increase in *C*. *albicans*. Intriguingly, when samples extracted with the PSP kit with Lysis Buffer P (PL10e6k and PL10e6b) were compared with those extracted with the Stabilizer (PS10e6k and PS10e6b), beta diversity calculated by Bray-Curtis distance (Fig. 2C) showed that the PS10e6k and PS10e6b samples clustered together, whereas the PL10e6k and PL10e6b were separate and closer to Q10e6. The Stabilizer seemed to be a key factor in the extraction. Moreover, when samples were treated with Stabilizer and then bead beaten (PS10e6b), a greater abundance of both *Candida* species and *M*. *furfur* was obtained. *S*. *cerevisiae* DNA was extracted by all methods and while it was already present in the sample, its abundance was much higher when using the PSP kit with the Stabilizer. The extraction of *C*. *neoformans* was challenging regardless of the method used: the greatest relative abundance was found with the Qiagen kit (Fig. 2B). The results for *P*. *crysogenum* were also interesting: its abundance was higher with the Qiagen kit and PSP with the lysis buffer than with the PSP kit with the Stabilizer buffer.

**Figure 3:**
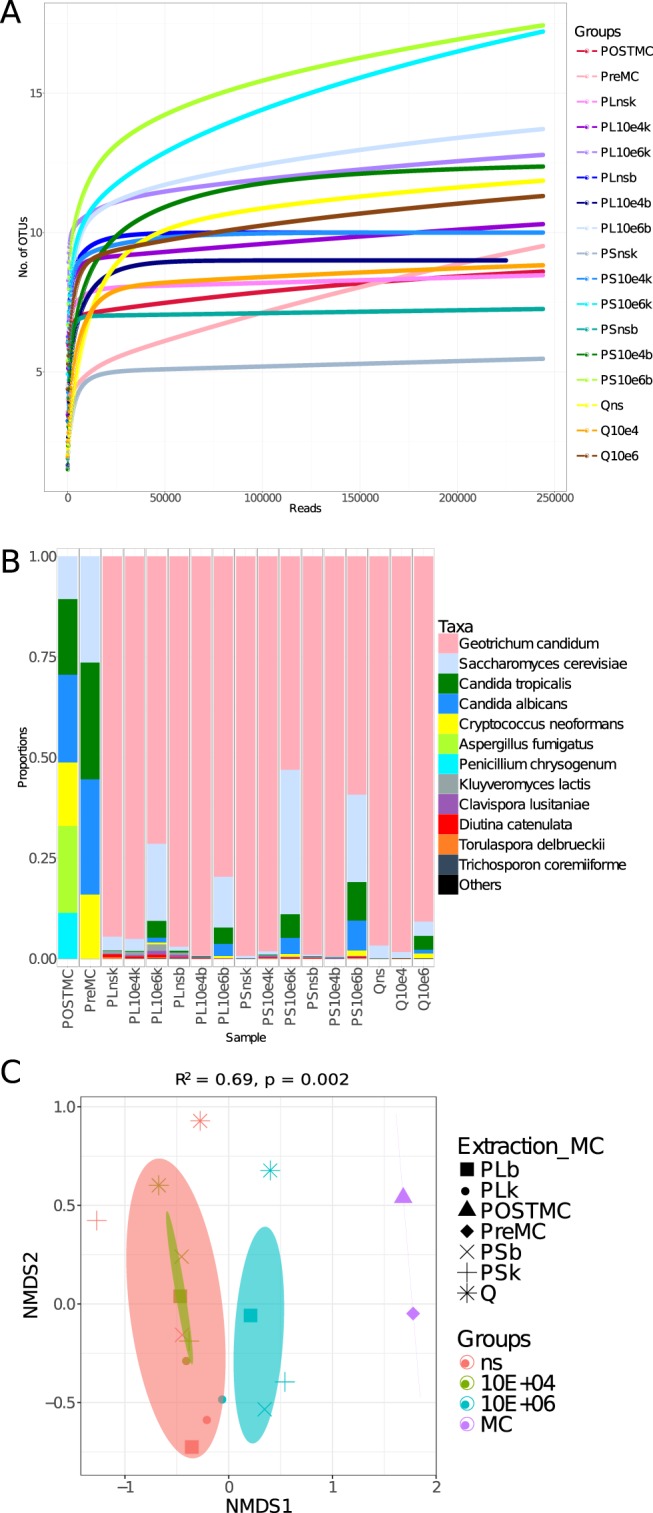
Use of the ITS2 (ITS3tagmix/ITS4NGS) primer set to define fungal diversity in stool (n = 15) and mock community (n = 2). (**A**) Rarefaction curves showing the number of OTUs versus the number of reads per sample. (**B**) Taxa summary showing the relative abundance of species, with each bar representing a sample. (**C**) Ordination of samples according to their community, calculated through NMDS, was produced using Bray-Curtis distance. The ellipses were drawn at the 95% confidence interval of standard error and the mean value of the groups. PERMANOVA was used to assess if the clustering was significant. The *R*^2^ refers to the percentage of variability explained by the groups in terms of microbial community structure. A list of sample names, each with a related description, can be found in Table 1.

OTU richness results (Fig. 2B) showed that the highest number of species was identified with the highest spike (10^6^ cells) as expected. This result was consistent when analysing the samples with ITS2 primer sets (Fig. 3). Beta diversity results for 18S rRNA amplicons (Fig. 2C) showed that the samples clustered according to the spike; in the unweighted UniFrac chart, a gradient from the non-spiked samples to the samples spiked with 10^6^ cells was observed, with the 10^4^-spiked samples in the centre. When abundance was taken into account (Bray-Curtis distance), the non-spiked and the 10^4^-spiked samples clustered more tightly.

### Primer set performance

The 5 primer sets gave different taxonomic profiles. The ITS primers lacked some of the species of the mock community. This was observed more in the pre-PCR mock community (Table 2), showing that this was related to amplification biases. Moreover, primers amplifying the small and large rRNA subunits showed a higher number of extra OTUs in the pre-PCR mock community (Table 2), suggesting the amplification of spurious products in mixed samples. These observations are described in detail for each primer set in the next sections; moreover, a summary of sequencing results with the number of raw and quality filtered reads, chimeras and number of OTUs and reads in the OTU tables can be found in Supplementary Table S1.

**Table 2:** Performance summary for each primer set in terms of MC species identified and inflation of species.

Species	18S rRNA		ITS2 (ITS3tagmix/ITS4NGS)		ITS2 (gITS7/ITS4NGS)		ITS1		28S rRNA	
	PreMC	POSTMC	PreMC	POSTMC	PreMC	POSTMC	PreMC	POSTMC	PreMC	POSTMC
*C*. *albicans*	√	√	√	√	√	√	√	√	√	√
*C*. *tropicalis*	√	√	√	√	√	√	√	√	√	√
*C*. *neoformans*	√	√	√	√	√	√	√	√	√	√
*M*. *furfur*	√	√	√	√	√	√	√	√	√	√
*S*. *cerevisiae*	√	√	√	√	√	√	√	√	√	√
*A*. *fumigatus*	√	√	X	√	X	√	X	√	√	√
*P*. *chrysogenum*	√	√	X	√	X	√	X	√	√	√
Extra OTUs	√	√	X	X	X	X	√	√	√	√
N of OTUs	33*	13	14	9	14*	16	27	15	58	29

The lack of some species in the pre-PCR compared to the post-PCR mock community using ITS primers suggests that the primer sets bias against these species during their amplification in mixed samples. In the same way, the observation of extra OTUs in the pre-PCR mock community with 18S and 28S rRNA primers suggests that spurious products were produced by these primers when using a template made of DNA from multiple species. NOTE: To be considered present (√), OTUs had to have more than 10 reads. However, all the OTUs were considered in the OTU total count. Details of OTU numbers are in Supplementary Table S1. *Average of the two replicates.

#### 18S rRNA

The two most significant outcomes of using this primer set were (i) The primers retrieved all the species in the mock community samples (Fig. 2B) and assignment was possible at genus/species level, even though this marker has been considered too conserved for such a deep classification in the taxonomic hierarchy; and (ii) the rarefaction curve in Fig. 2A shows that the number of OTUs is inflated; only 7 species were expected in the mock community. This number is close for the post-PCR mock community (13 instead of 7) and may be related to the presence of two different OTUs assigned to *C*. *tropicalis* and a few other OTUs with a very low number of reads (<60) assigned to *Saccharomyces*. The pre-PCR mock community was more inflated (33 OTUs were recorded). It was speculated that the extra OTUs were spurious products created during the amplification process.

To further explore these two outcomes, the OTUs were aligned and analysed. The 8 OTUs of the post-PCR mock community (3 OTUs had <10 reads and were excluded) showed that the OTU assigned to *C*. *albicans* differed only by 1 base with one *C*. *tropicalis* OTU and by 2 bases with the other OTU assigned to this species. By contrast, the OTU assigned to *S*. *cerevisiae* differed by 10 bases (almost 3%), showing why a discrimination was obtained among these Saccharomycetales. The alignment showed that *A*. *fumigatus* and *P*. *chrysogenum* were also very similar. These belong to the same family (Trichocomaceae) and their sequences differed by 3 bases.

To understand the inflation in the number of OTUs, the 8 OTUs found in the post-PCR mock community were used as a gold standard and compared with the 22 OTUs found in excess in the pre-PCR mock community. The alignment showed that these were indeed chimeras, sharing their sequence with 2 or more of the gold standard OTUs. For example, the chart (Fig. 2) shows that two extra species (not included in the mock community) were rather abundant: *Torulospora delbrueckii* and *Candida oleophila*. Their alignment was analysed against the gold standard OTUs and we found that these were in fact chimeras (see Supplementary Table S3).

During the preparation of the pre-PCR mock community, an attempt was made to balance the amount of the 7 species added by adjusting the concentration according to the Cp value. Despite this effort, the proportion of some species was lower, especially *A*. *fumigatus*, *M*. *furfur* and *P*. *crysogenum*. This discrepancy may be explained by the fact that different amplification conditions between the qPCR and amplicon production were used. Moreover, other factors such as the different number of gene copies in the genome of the seven strains may have contributed to this discrepancy. No mismatches were detected for this primer set (see Supplementary Table S2), indicating this was not the reason for the imbalance observed in pre-PCR community profile. As shown in Fig. 2B, the stool sample is rich in a single species (*G*. *candidum*); this species was assigned to the OTU using BLASTN (see Methods section for details), as SILVA assigned it to an “uncultured Geotrichum”.

#### ITS2 (ITS3tagmix/ITS4NGS set)

While 18S rRNA primers enabled the assessment of diversity in the mock community, this was not the case for the ITS3tagmix/ITS4NGS primer set. The post-PCR mock community showed all of the species. However, *M*. *furfur* was present in much lower abundance (0.04% for the post-PCR and 0.01% for the pre-PCR mock community) compared to the other species (see Fig. 3B and Supplementary Table S1). Because its amplification product was longer than that of other species (>600 bp), we initially assumed that *M*. *furfur* was missing because the reads were too long to be paired during data analysis. To explore this further, the unpaired reads (R1 and R2) for this sample were analysed separately. Despite this, *M*. *furfur* reads were very low. The most likely explanation is that the longer amplicons which were already present at a low abundance compared to shorter reads (see fragment analyser output: Supplementary Fig. S1) were biased against during clustering on the sequencing flowcell because of their length^43^. Secondly, the pre-PCR mock community lacked *A*. *fumigatus* and *P*. *crysogenum* (Fig. 3B). The pre-PCR mock community results were confirmed by the results obtained from stool samples, where moulds were not observed in any of the samples spiked with 10^6^ cells, indicating that in a mixed sample, the primers seem to bias against these species. Primer mismatches were explored also for this set (see Supplementary Table S2) and again no mismatches were detected. Other factors, such as the different number of template copies in the genome of the species and the use of different PCR protocols for the qPCR assay to prepare the mock community and the amplicons production, which in this case also included different primers, could have contributed to this observation. In terms of number of OTUs amplified by these primers (see Fig. 3A), the number of species was less inflated than that seen for 18S rRNA (Table 2). The OTU table (Supplementary Table S1) showed that the abundance of the extra 2 OTUs in the post-PCR and 8 OTUs in the pre-PCR mock communities was very low (<10 reads) and could be excluded as noise or contamination. As observed with 18S rRNA, the community from the sample had a very high relative abundance of a single species/OTU (*G*. *candidum*) (Fig. 3B). This was originally assigned to *Dipodascus australiensis*; however, when running the read in BLASTN, it was assigned to *G*. *candidum* (consistent with the 18S rRNA marker). Finally, beta diversity results (Fig. 3C) were similar to those found with 18S rRNA primers.

#### ITS2 (gITS7/ITS4NGS set)

**Figure 4:**
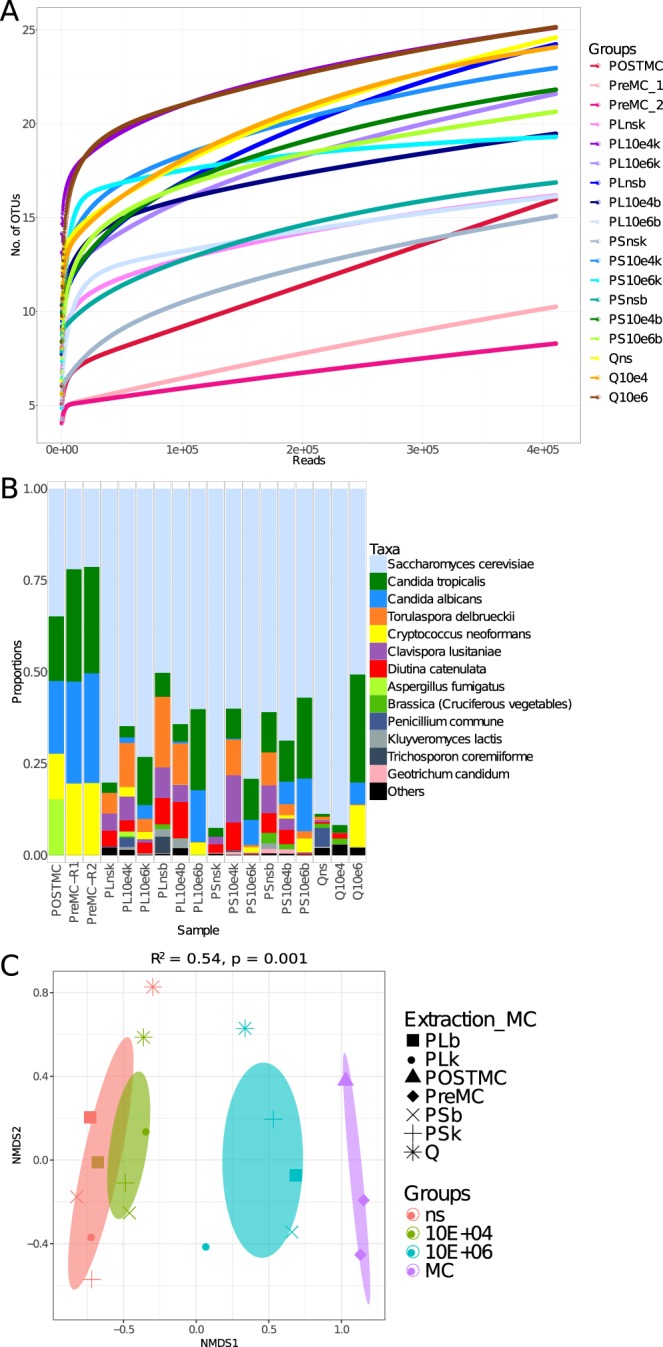
ITS2 (gITS7/ITS4NGS) primer set performance in defining fungal diversity in stool (n = 15) and mock community (n = 3). (**A**) Rarefaction curves showing the number of OTUs versus the number of reads per sample. (**B**) Taxa summary showing the relative abundance of species, with each bar representing a sample. (**C**) Ordination of samples according to their community, calculated through NMDS, was produced using Bray-Curtis distance. The ellipses were drawn at the 95% confidence interval of standard error and the mean value of the groups. PERMANOVA was used to assess if the clustering was significant. The *R*^2^ refers to the percentage of variability explained by the groups in terms of microbial community structure. A list of sample names, each with a related description, can be found in Table 1.

As with the first ITS2 primer set (ITS3tagmix/ITS4NGS), very little *M*. *furfur* was observed (Supplementary Table S1). *Aspergillus* abundance was also very low. Moreover, the beta diversity results (Fig. 4C) showed similar clustering to that observed with 18S rRNA and ITS3tagmix/ITS4NGS sets. Apart from these similarities, several discrepancies were found with this set: reads assigned to *Penicillium* were very low in the POSTMC sample (41 reads; see Supplementary Table S1). However, this genus was found in some of the stool samples, both spiked and non-spiked (see Supplementary Table S1). The stool sample had a very low relative abundance of *G*. *candidum* (which dominated with 18S rRNA and ITS3tagmix) but showed a very high abundance of *Saccharomyces*. Moreover, this primer set gave extra taxa compared to the previous two primer sets: i.e. *C*. *tropicalis* was recorded in most of the samples, along with *Torulospora*, *Clavispora* and others (Fig. 4B). The number of OTUs observed in the non-spiked samples was higher than that which was observed when amplified using the ITS3tagmix/ITS4NGS primer set (Fig. 4A and Table 2).

#### ITS1

**Figure 5:**
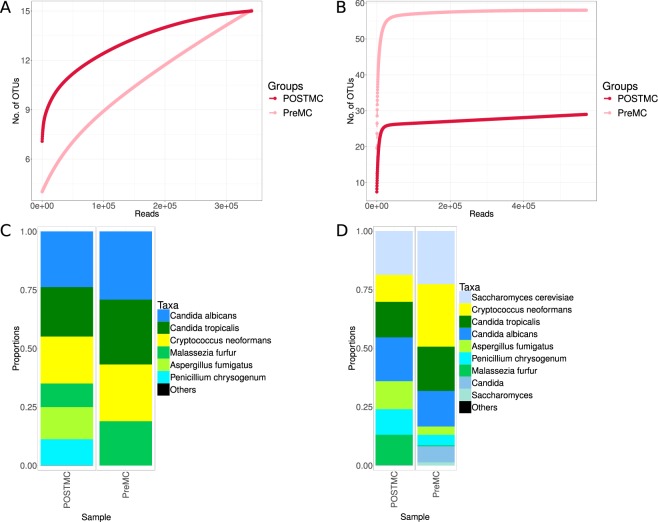
Performance of the ITS1 primer set in the mock community (n = 2) (**A**,**C**) and 28S rRNA in the mock community (n = 2) (**B**,**D**). ITS1 (**A**) and 28S rRNA (**B**) rarefaction curves showing the number of OTUs versus the number of reads per sample. ITS1 (**C**) and 28S rRNA (**D**) taxa summaries showing the relative abundance of species with each bar representing a sample. A list of sample names, each with a related description, can be found in Table 1.

This set showed similar biases to the two ITS2 primers. However, in this case, *Saccharomyces* was in very low abundance because of the length of its product (>600 bp). Two OTUs were assigned to *S*. *cerevisiae* and a total of 269 reads were assigned to this species in the POSTMC and 31 in the PreMC sample (Supplementary Table S1). As shown with the other ITS primer sets, when in a mixed sample, the primers appeared to bias against *A*. *fumigatus* and *P*. *crysogenum* amplification (Fig. 5C). Contrary to what was observed for the three primer sets previously described, mismatches were observed for this primer set; ITS1-F had mismatches against *S*. *cerevisiae*, *A*. *fumigatus* and *P*. *crysogenum*, while ITS2r showed mismatches against *C*. *neoformans* and *M*. *furfur* (see Supplementary Table S2).

#### 28S rRNA

This primer set gave interesting results: it was able to amplify most of the species in both mock community types. Nevertheless, it seemed to bias against *M*. *furfur*, as only a small amount was observed in the PreMC sample (Fig. 5D and Supplementary Table S1). This may be related to the fact that the reverse primer (LF402) showed a mismatch against this species (see Supplementary Table S2). However, the main issue was the lack of a comprehensive database, which prevented the classification of OTUs. *Candida* spp. were not classified with the database, but manually using BLASTN.

As observed for 18S rRNA, more spurious products were observed than with ITS, confirmed by an inflation in the number of OTUs (Fig. 5B, Table 2 and Supplementary Table S1). To explore this further, the OTUs’ sequences were aligned. The post-PCR mock community showed 7 OTUs (one for each strain of the mock community) that were very abundant (>10^4^ reads) and 13 OTUs had between 100 reads and up to 264 reads. The first, very abundant, OTUs were used as gold standards and the second were considered errors. Analysis of the alignment showed that these were not chimeras but errors. For example, for some OTUs (i.e. OTU5, OTU6, OTU8, OTU12, OTU19 and OTU124) the primers were not correctly trimmed, causing an excess in the OTUs picked. In other cases, up to two base differences with the gold standard caused the detection of extra OTUs (i.e. OTU7, OTU51, OTU38, OTU121, OTU144 and OTU150). The pre-PCR mock community OTU table showed the same 7 gold standard OTUs with a high abundance, the lowest having 4255 reads (*M*. *furfur*) and the highest 299,044 reads (*C*. *neoformans*). Of the 49 OTUs in excess with more than 50 reads, 6 had more than 5000 reads (up to 17590 reads). All these were assigned to Saccharomycetales species belonging to genera such as *Candida* and *Saccharomyces*. The alignment of these OTUs showed that these were chimeras of the gold standard OTUs (see Supplementary Table S3).

#### Negative controls

Most of the negative controls were clean (i.e. the number of reads was below our threshold of 1000). Two exceptions were observed: the amplification negative control for the primer set ITS3tagmix/ITS4NGS and the DNA extraction negative control for the method PLb (Table 1) amplified with the gITS7/ITS4NGS primer set. The former gave 3436 high-quality reads, 98.75% of which were assigned to a single OTU, classified as *Aureobasidium pullulans*. This is a ubiquitous Ascomycetes yeast^44^. Considering that all the other PCR controls were clean, a contamination of the reagents can be excluded; therefore, this likely occurred during the preparation of the PCR. The latter negative control gave 1284 reads, of which 99.5% were assigned to a single OTU classified as *Malassezia restricta*, a species that is commonly found on the skin of healthy people^45^. While the contamination may have occurred during the extraction, it cannot be excluded that the kit reagents were contaminated.

### Primer comparison with stool samples

**Figure 6:**
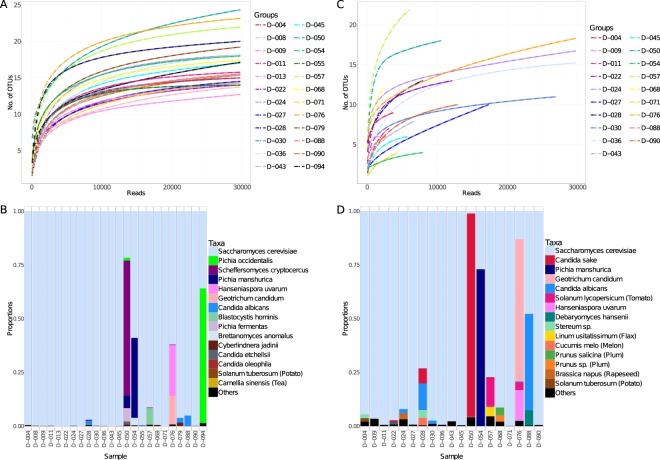
Sequencing results of 18S rRNA (**A**,**B**) from donors (n = 24) and ITS2 (**C**,**D**) (n = 19). (**A**) Rarefaction curves showing the number of OTUs versus the number of reads per sample. (**B**) Taxa summary at species level, with each column representing a sample. (**C**) Rarefaction curves showing the number of OTUs versus the number of reads per sample. (**D**) Taxa summary at species level, with each column representing a sample.

The first observation made when comparing the performance of the 18S rRNA and ITS3tagmix/ITS4 sets was that the latter produced a very low amount of amplification product. When comparing the fragment analyser traces (Supplementary Fig. S2) of the 24 samples tested, only 4 samples gave a signal comparable to that observed with the 18S rRNA primer set. Twelve samples gave smaller peaks and the rest did not generate any product. All of the samples showed a large signal for a short (<200 bp) nonspecific product, likely a primer dimer. In spite of the poor PCR yield of some samples, all were sequenced. During the filtering of reads, samples with <2000 reads were discarded, leaving only 19 samples for analysis. The low PCR yield was reflected in the low number of reads gathered, as shown in the rarefaction curve in Fig. 6C. Taxonomy profiles were similar (Fig. 6B for 18S rRNA and Fig. 6D for ITS2 (ITS3tagmix/ITS4 set)): while most of the samples contained mainly *Saccharomyces cerevisiae*, 6 donor stool samples showed a more diverse profile. These were: D-028, D-050, D-054, D-057, D-076 and D-088. The assignment of the OTUs and the diversity were not always consistent between the two primer sets. For certain OTUs, this was due to discrepancies between the databases used for ITS2 (UNITE) and 18S rRNA (SILVA). For example, OTU5 (ITS2) and OTU49 (18S rRNA) were the same species, but UNITE assigned OTU5 to *Pichia mandshurica*, while SILVA assigned OTU49 to *Candida ethanolica*. This was solved when running the OTUs in BLASTN and both were assigned to *P*. *mandshurica*. For other OTUs, we could not explain these differences, which may be due to the different taxonomical resolution between the two markers or they may be related to biases during the amplification or sequencing steps, as described in previous sections. Nevertheless, some species seem to be consistent between samples. For example, *C*. *albicans* was recorded in samples D-024, D-028, D-030 and D-088 by both primer sets. It must be noted that its relative abundance was higher when using ITS2 primer set. For this set, several OTUs were not assigned to any taxa. When these were evaluated in BLASTN, it was found that they belonged to plants: *Solenum* (tomato and potato), *Lynum* (pale flax), *Cucumis* (melon), *Prunus* (plum) and *Lactuca* (lettuce), and were very likely to have originated in the subjects’ diet. The 18S rRNA primers also amplified other eukaryotic DNA (e.g. *Blastocystis*), but in a lower relative abundance than that observed using the ITS2 primers.

## Discussion

Microbial ecology studies are known to be influenced by the methods used. What we observe is a function of what we are able to extract, amplify and sequence. These biases have been extensively evaluated for the study of bacterial communities of the human GI tract^8,46^, although the methods to resolve bias have yet to be fully established. Regarding fungal communities, only a few studies have reported the effectiveness of the methods used^6,7,25^. Our study evaluated several DNA extraction approaches of pure fungal cultures and of fungal strain-spiked stool samples and analysed stool samples from a cohort of donors. We have established that DNA extraction techniques can bias the community profile by being more effective with certain species than others. We also tested five primer sets that amplify fungal rDNA and have demonstrated how primer selection can significantly influence outcomes, both through preferential amplification and with respect to the relative quality of reference databases available. Finally, we have shown that when using markers with a variable length, the sequencing method also influences the outcome by biasing against longer reads.

The extraction of metagenomic DNA from stool is made challenging by the nature of the stool matrix and requires the use of specific reagents to reduce DNA degradation and neutralise inhibitors^47^. The PSP kit with the Stabilizer buffer addresses both of these issues. Its effectiveness for the extraction of fungal DNA has been shown in one study^25^, and our initial hypothesis was that by adding an additional bead beating step, we could improve the extraction of fungal DNA. The effectiveness of bead beating on sturdy fungal structures including spores was observed in other studies (in combination with other methods/kits)^48,49^ and it is considered a better alternative than the use of an enzymatic step, because of the lower cost and shorter processing time^50^. It was interesting to see that this was observed only for two species (*C*. *tropicalis* and *M*. *furfur*) out of seven, and that the bead beating actually decreased the extraction of a fungus (*C*. *neoformans*). More striking was the comparison of the PSP kit and the Qiagen kit. On the one hand, the fact that the Qiagen kit was effective for the extraction of *A*. *fumigatus* spores was not surprising, as this has been already shown in studies focused on the diagnosis of invasive aspergillosis^49^. We confirmed this result through quantification, but also with a sequencing-based experiment, showing that a higher relative abundance of *A*. *fumigatus* is gathered with the Qiagen kit. The PSP kit, on the other hand, was more efficient in extracting *Candida* and *Malassezia* DNA, especially when bead beating was combined with the Stabilizer buffer. Our work confirms that DNA extraction methods influence the community profile, and in order to maximise the diversity of species extracted, we took the informed decision to use both methods (PSb and QSK) to extract DNA from stool. A limitation of our experimental design is the use of spores for *A*. *fumigatus* instead of hyphae. This fungus is normally found in the latter state when active^14^. However, spores were chosen because these allow an exact quantification of the number of cells used during the extraction and therefore enable the standardisation of the extractions from pure cultures and the spiking of stool.

The comparison of four markers (18S and 28S rRNA, ITS1 and ITS2) and five primer sets produced some intriguing results. First, in contrast to a previous study^25^ and the general view of this marker^31^, 18S rRNA performed similarly to the other markers in terms of depth of classification. This set was not only able to distinguish all of the mock community species, but also those that were phylogenetically close (i.e. *C*. *albicans*, *C*. *tropicalis* and *S*. *cerevisiae*). Moreover, when comparing its performance using stool samples, this set gave a higher yield of PCR product and more of its reads were assigned to fungi than ITS2, which amplified more plant/food DNA. 18S rRNA was also able to gather a diversity profile similar to ITS2. These outcomes may be related to the use of a primer set specific for fungi and thoroughly tested with human pathogens^42^. Two limitations of using 18S rRNA are that (i) it is difficult to assess if any diversity (outside the mock community) is lost because of the high level of conservation of the gene, and therefore it is not recommended to rely on a classification at species level in “black box” samples, whose taxonomic profile is discovered at the end of the experiment; and (ii) the use of an rRNA gene (18S or 28S for fungi or 16S for bacteria) made of variable and conserved regions increases the chance of producing spurious products, i.e. chimeras generated during amplification that inflate diversity^46,51^. This issue may be attenuated by reducing the number of cycles during PCR^46^, along with the use of bioinformatic tools to filter out chimeras. Production of spurious products is reduced with ITS primer sets, as by being hypervariable in its entire length, the formation of chimeras during amplification is more unlikely^51^. This was confirmed in our study: when taking abundance into account, the percentage of chimeras obtained with ITS primers was at least 16 times lower compared to the rRNA primer sets; this was 3.2% for 18S rRNA, 0.2% and 0.1% for the two ITS2 primer sets respectively, 0% for ITS1 and 5.5% for 28S rRNA (see Supplementary Table S1). Despite these limitations, the use of this 18S rRNA primer set was very promising for the study of the gut mycobiome; also, compared to priming for ITS, it allowed phylogenetic analysis, the product had a regular length (reducing PCR and sequencing biases) and it gave a higher PCR yield when amplifying DNA from a matrix of stool. Moreover, it can be used when RNA (or cDNA) is the template in amplicon sequencing studies. The 18S rRNA database from SILVA is very comprehensive and allowed us to classify at genus/species level for most OTUs. This was not the case for 28S rRNA.

If 18S rRNA gave us the mock community profile closest to that which we expected, this was not the case for the ITS sets. All three primer sets tested showed two principal drawbacks: (i) preferential amplification and (ii) sequencing bias due to the variable length of the ITS amplicons. The first explains why *A*. *fumigatus* and *P*. *crysogenum* were not found in the pre-PCR mock community with any of the ITS primer sets. This indicates that when these are present in a mixed sample, their amplification is biased against. This also occurred with *Geotrichum candidum* in stool amplified with gITS7/ITS4NGS. This OTU was detected at much lower levels with this primer set, giving taxonomic profiles for the stool sample (spiked and non-spiked) that were radically different from those we obtained with 18S rRNA and ITS3tagmix/ITS4NGS-targeted primer sets.

The second issue was related to the sequencing process. Certain species (*M*. *furfur* with ITS2 and *S*. *cerevisiae* with ITS1) gave amplicons that were lost because of their length. This raises the question of how much diversity is unknowingly lost in metagenomic metabarcoding studies (with actual environmental samples) when ITS is sequenced using an Illumina platform (HiSeq or MiSeq). With this work, we confirmed the issue related to preferential amplification and ITS markers^6^ and uncovered, for the first time, to our knowledge, ITS issues related to the length of the amplicons and sequencing platforms.

A final issue was related to the analysis of the data. We showed that, by using different markers and therefore databases, different results were obtained in terms of taxonomical assignment, with some markers (i.e. 28S rRNA) being particularly weak in this aspect^6^. We harmonised the outcomes of different markers in this study by evaluating all of the OTUs in the NCBI web tool BLASTN. This issue can make the comparison of different studies misleading, particularly when different markers and databases are used and the taxonomy assignment is not cross-validated. Moreover, an attempt to unify the nomenclature of all databases should be made, along with a thorough evaluation of spurious products, especially for SSU and LSU markers.

In conclusion, we have demonstrated how three main steps (DNA extraction, choice of primer and taxonomy assignment) of amplicon sequencing for the characterisation of the gut mycobiome can bias results. It is therefore important to test these methods, select them according to the environment studied and be aware of their limitations before drawing conclusions. Based on the observations presented in this study, we believe that combining two extraction methods (PSb and QSK) allows the extraction of fungal DNA from stool to be maximised. For the characterization of the gut mycobiome we recommend the 18S rRNA primer set. This primer set is highly specific towards fungi, allowing phylogenetic analysis and producing a classification comparable to ITS.

## Methods

### Preparation of cells

Seven fungal strains were tested: *Candida albicans*, *Candida tropicalis*, *Saccharomyces cerevisiae*, *Cryptococcus neoformans*, *Malassezia furfur*, *Aspergillus fumigatus* and *Penicillium chrysogenum*. All were clinical isolates (Liverpool Clinical Laboratories of Infection and Immunity, Royal Liverpool and Broadgreen University Hospitals, NHS Trust), except for *Malassezia furfur*, which was from the CBS collection (CBS 1878). The identity of all strains was confirmed by Sanger sequencing (GATC Biotech Ltd.) of ITS2 with ITS3tagmix/ITS4NGS PCR product of each of these (for sequences, see Supplementary Data S1). *C*. *albicans*, *C*. *tropicalis*, *S*. *cerevisiae* and *C*. *neoformans* were grown in Yeast extract-peptone-dextrose agar (YPD; Sigma-Aldrich) containing 26 mg/L chloramphenicol (Sigma-Aldrich) for 2 days at 30 °C. Afterwards, a single colony of each strain was inoculated in 8 mL of YPD broth at 30 °C, with overnight shaking at 200 rpm. *M*. *furfur* was grown in modified Dixon’s (mDixon) agar^52^ containing 26 mg/L chloramphenicol for 3–4 days at 32 °C. An 8 ml volume of mDixon broth^52^ was then inoculated with a single colony and incubated at 32 °C, shaking at 200 rpm for 48 hours. To assess cell concentration, the *OD*_600_ of a ten-fold dilution from each culture broth was measured with a GeneQuant Pro RNA/DNA calculator spectrophotometer (GE Healthcare). A standard curve of *OD*_600_/CFU was previously produced for each strain. Quarter strength sterile Ringer solution (Sigma-Aldrich) was used as diluent. *A*. *fumigatus* and *P*. *chrysogenum* were inoculated in Sabouraud dextrose agar (SDA) containing chloramphenicol (Sigma-Aldrich); *A*. *fumigatus* for three days at 37 °C, and *P*. *chrysogenum* for 5–7 days at room temperature. Spores were harvested from the SDA plate with a sterile swab and suspended in 10 mL of sterile PBS pH 7.3 containing 0.05% v/v Tween 20 (Sigma-Aldrich). Following incubation for 10 minutes to allow the hyphae to deposit in the bottom, a 2 mL volume was then taken from the top, and spores were counted with a Neubauer improved haemocytometer. Yeast cell and spore concentrations were adjusted to spike 1.2 mL of Lysis Buffer P (PSP®Spin Stool DNA Kit, Stratec) with 1X10^6^ cells/spores. Because the QIAamp®Fast DNA Stool Mini Kit (Qiagen) protocol carries a lower volume of starting material than the PSP kit (200 *μ*L instead of 800 *μ*L), the number of cells of *C*. *albicans* and spores of *A*. *fumigatus* were increased to use the same amount of material. DNA was extracted immediately (see DNA extraction section).

### Stool samples

A stool sample from a healthy volunteer was collected and immediately brought to the laboratory for processing. The spiking and aliquoting processes are summarised in Fig. 1B. Briefly, twelve aliquots were made; four were spiked with 10^6^ cells of each species, four with 10^4^ cells and four were not spiked. On the day of the extraction, 3.6 mL of Lysis Buffer P (PSP®Spin Stool DNA Kit, Stratec) was added to two aliquots of each spike (10^6^, 10^4^, non-spiked) and DNA was extracted immediately (see next section for details). 3.6 mL of Stabilizer (PSP Spin Stool DNA Plus Kit, Stratec) was added to a further two aliquots of each spike (10^6^, 10^4^, non-spiked), which were vortexed briefly and incubated at RT overnight. Each tube was split in three and three extractions for each extraction method and spike were carried out in parallel. Because the extraction with the Qiagen kit was performed at a later timepoint, the sample was frozen, not fresh, when spiked. Nevertheless, the sample was aliquoted, spiked and stored as described above.

Stool samples used to compare the performance of 18S rRNA and ITS2 primers were collected at the Royal Liverpool University Hospital (UK). In accordance with ethical approval from 15/NW/0045, as part of the EU-funded SysMedIBD (Systems medicine of chronic Inflammatory Bowel Disease) Project. Written, informed consent was obtained from volunteers before they donated a faecal sample. Samples were brought to the laboratory the same day of clinic and stored at −80 °C until extraction. DNA was extracted from 220 mg of stool.

### DNA extraction

Half of the samples extracted with the PSP kit underwent a bead beating step. For this step, 0.5 g of 0.1 and 0.5 mm zirconia/silica beads (BioSpec) and 5 Zirconia II beads from the PSP®Spin Stool DNA Kit were used. Briefly, all the samples were first heated at 95 °C, with mixing at 900 rpm for 10 minutes in a Thermomixer (Eppendorf). Half of the samples were then processed following the kit protocol (Protocol 2^53^); the other half were bead beaten for a total of 6 minutes at 30 Hz in a TissueLyser II (Qiagen®). After every 1.5 minutes of bead beating, the samples were incubated in ice for one minute. After this step, all the samples were processed in parallel as per kit protocol. The extraction with the QIAamp®Fast DNA Stool Mini Kit (Qiagen®) was performed following the kit protocol “Isolation of DNA from Stool for Pathogen Detection”^54^.

A negative control (without starting material) was also extracted each time. For the extraction from cells or spores, DNA was eluted from the column in 100 *μ*L buffer; when extracting from stool samples, the elution was repeated for a total of 200 *μ*L. Extractions from pure cultures were done in triplicate and each replicate was analysed separately. For the spiked stool sample, extractions were carried out in triplicate, and 20 *μ*L from each replicate was pooled and used for downstream analysis. Following extraction, DNA was quantified with a Qubit (Qubit dsDNA HS assay Kit, Life Technologies). Stool samples from donors were extracted with the methods PSb and Q (Table 1). The Qubit assay was used to quantify DNA, which was normalised to 10 ng/*μ*L and pooled.

### Gene quantification

18S rRNA gene quantification was achieved using the FungiQuant Assay^42^. PCR reactions were made in a total volume of 10 *μ*L using 1X PrimeTime®Gene Expression Master Mix (IDT), 1.8 *μ*M of each primer^42^ (Table 3), 225 nM of LNA PrimeTime probe (/5′6-FAM/TG + GTG + CATGG + CC + GTT/3IABkFQ/) (IDT), 3.5 *μ*L of template and Ultrapure DNase/RNase-free water (Life Technologies). The reactions were run in a Roche LightCycler 480 with the following program: 95 °C for 3 minutes, then 45 cycles of 95 °C for 15 seconds and 65 °C for 1 minute. To assess assay efficiency, a calibration curve was made for each species where a quantified amount of genomic DNA was diluted, and serial ten-fold dilutions made. To quantify the number of gene copies in stool samples, a calibration curve was made with known concentration of a gBlock (IDT) of the 18S rRNA amplicon sequence from *C*. *albicans* SC5314. Cp values and efficiencies were calculated with the LightCycler 480 Software (v 1.5.0)^55^ and gene quantification analyses were made using the Second Derivative Maximum Method^56^. Samples were run in triplicate along with triplicates of a negative control (no template) and a positive control (calibrator). All negatives controls were clean (no signal), or when a signal was detected this had a Cp value >3 compared to the most diluted point of the calibration curve. Efficiencies and calibration curves are shown in the Supplementary Fig. S3.

**Table 3:** Primers used for amplicon production and qPCR.

Name	Sequence 5′ to 3′	Marker	References
Forward overhang	ACA CTC TTT CCC TAC ACG ACG CTC TTC CGA TCT	na	^46^
Reverse overhang	GTG ACT GGA GTT CAG ACG TGT GCT CTT CCG ATC T		^46^
FungiQuant-F (forward)	GGR AAA CTC ACC AGG TCCA G	18S rRNA	^42^
FungiQuant-R (reverse)	GSW CTA TCC CCA KCA CGA		^42^
ITS1-F (forward)	CTT GGT CAT TTA GAG GAA GTA A	ITS1	^40^
ITS2r (reverse)	GCT GCG TTC TTC ATC GAT GC		^41^
ITS3tagmix (forward)	CTA GAC TCG TCA NCG ATG AAG AAC GYR G	ITS2	^34^
gITS7 (forward)	GTG ART CAT CGA RTC TTT G		^86^
ITS4NGS (reverse)	TTC CTS CGC TTA TTG ATA TGC		^34^
LR0Rngs (forward)	ACS CGC TGA ACT TAA GC	28S rRNA	^34^
LF402 (reverse)	TTC CCT TTY ARC AAT TTC AC		^34^

### Amplicon production and sequencing

The primers tested are shown in Table 3. A universal tail tag dual index barcoding approach^46^ was used. In the first PCR round, reactions were made in a total volume of 20 *μ*L, with 0.02 U/*μ*L Q5 High-Fidelity DNA Polymerase (NEB), 1X Q5 Reaction Buffer (NEB), 0.125 *μ*M of each primer (overhang + specific primer, Table 3) (HPLC grade, IDT), 200 *μ*M of dNTPs (NEB) and Ultrapure DNase/RNase-free water (Life Technologies) to reach the volume. For the stool samples, 10 ng of DNA was used per reaction. Meanwhile, two types of mock community amplicons were produced. In the first, DNA from each species was adjusted to the same Cp value (see Gene quantification section for details) and pooled (PreMC). In the second, DNA from each species was amplified separately and the same amount was pooled after the second PCR (POSTMC). Samples were amplified in a thermocycler (Multigene®and Multigene®OptiMax, Labnet International) with the following program: 98 °C for 30 seconds, then 15 cycles at 98 °C for 10 seconds, followed by 55 °C (ITS2 and 28S primer sets)/56.1 °C (ITS1)/62 °C (18S) for 30 seconds and 72 °C for 20 seconds, followed by a final extension at 72 °C for 2 minutes. Amplicons were then purified with the AxyPrep Mag PCR Clean-up kit (Axygen) and eluted in 10 *μ*L.

The second PCR was carried out using the same amount of polymerase, buffer and dNTPs as per the first PCR. However, this time 0.25 *μ*M of each index primer^46^ (TruGrade, IDT) was used. The whole volume of purified amplicons was used as template. The amplification program was 98 °C for 30 seconds, then 20 cycles at 98 °C for 10 seconds, followed by 65 °C for 30 seconds and 72 °C for 20 seconds, followed by a final extension at 72 °C for 2 minutes. Negative controls of the stool DNA extraction and of the PCR (no template) were also amplified; these did not give a visible product when run on an agarose gel. However, a few *μ*L were included in the pool and sequenced. All of the amplifications were performed in triplicate, and the replicates were pooled for a total of 60 *μ*L which was purified with the AxyPrep Mag PCR Clean-up kit and eluted in 25 *μ*L. Amplicons were quantified with a Qubit dsDNA HS assay Kit and electrophoresis was performed using a 1.3% w/v agarose gel (UltraPureTM Agarose, Invitrogen). Amplicons were then pooled equimolarly.

Samples were then sent to the University of Liverpool CGR for sequencing. There, the pool of amplicons was quantified by Qubit and the fragment distribution was assessed using an Agilent DNA high sensitivity kit (2100 Bioanalyzer, Agilent). The pool of amplicons was size selected within the 250–750 bp range using a 1.5% w/v agarose cassette on a PippinPrep instrument. The size-selected pool was quantified again by Qubit. The fragment distribution was checked and further assessed by qPCR, using the Illumina Library Quantification Kit (KAPA, cat. no. KK4854; Roche) on a Roche Light Cycler LC480II according to manufacturer instructions. The concentration obtained by qPCR was used in combination with Bioanalyzer data to prepare the template DNA for the sequencing run. To increase library diversity, 15% PhiX was used as spike-in. The sequencing was carried out on one lane of an Illumina HiSeq. 2500 with v2 2 × 300 bp paired-end sequencing chemistry. Stool samples from donors were prepared similarly, except that the fragment distribution of individual amplicons was assessed with an Agilent DNA high sensitivity kit before pooling. The length and the concentration were then used to pool the samples equimolarly. Libraries were sequenced with a MiSeq with v2 2 × 250 bp paired-end sequencing chemistry.

### Data analysis

qPCR data were analysed in R (v 3.4.2)^57^. Barplots and a boxplot were produced with ggbarplot() and ggpaired() functions respectively.

Demultiplexing, adaptor and quality trimming (Cutadapt v 1.2.1^58^ and Sickle v 1.2^59^) of reads was performed by the University of Liverpool CGR. Reads were then error-corrected with BayesHammer^60,61^ in SPAdes (v 3.7.0)^62^, merged with PEAR (v 0.9.10)^63^ and PhiX was removed. Primers were trimmed and samples with less than 1000 reads were excluded. Clustering was done with SWARM 2.0 (d = 3 for 18S and 28S rRNA and d = 2 for ITS)^64^. ITS reads were also processed with ITSx (v 1.0.11)^65^. Chimeras were filtered out with UCHIME^66^ ref mode. Taxonomy was assigned with BLAST^67^, with assign_taxonomy.py in MacQIIME (v 1.9.1)^68^. Reference databases used were SILVA^69^ (SILVA_123) for 18S rRNA, a curated database for 28S rRNA^70^, UNITE^71^ (01.12.2017 release) for ITS. An OTU table was produced (make_otu_table.py) and those of 18S and 28S rRNA amplicons were also filtered (filter_otus_from_otu_table.py) at a 0.05% threshold^72^. For 18S rRNA reads, alignment (align_seqs.py) and phylogeny (make_phylogeny.py) analyses were also carried out using MacQIIME (v 1.9.1)^68^ default algorithms (PyNAST^73^ and FastTree2^74^). Aligned OTUs were filtered (filter_alignment.py) to remove the 10% most variable positions, with an 80% gap filter threshold. It must be noted that the names of taxa assigned to OTUs were not always consistent among these databases and in some cases no name was given. Therefore, to unify the nomenclature among the taxonomy summary graphs (Figs 2B, 3B, 4B, 5C,D and 6B,D), OTUs were also run in BLASTN webtool^75^ excluding uncultured and environmental sample sequences, and the names were then changed manually. When the identity percentage was 100%, the name of the species was used; when this was <100%, genera were reported. OTUs assigned to the same species (or genus) are merged in the taxonomy summary. Mismatches between the primers and the reference sequences of the species of the mock community were assessed with Primer Prospector^76^ (analyze_primers.py function). Reference sequences were downloaded from NCBI^77^. If a reference sequence was not available for a species, the sequence of another species from the same genus was used. 18S rRNA, 28S rRNA and ITS2 sequences were aligned to explore the similarity among and inflation of OTUs. This alignment was made with MEGA X^78^, using clustalW^79^ (default parameters).

Statistical analyses were performed in R (v 3.4.2)^57^ using the OTU tables, phylogenetic trees (where applicable) and metadata associated with this study. Samples with less than 2000 reads were excluded from analysis. This threshold was justified by the need to find a minimum number of reads that would allow most of the alpha diversity to be gathered, as we found that samples with a number of reads lower than this threshold do not reach a plateau in the rarefaction analysis. In general, the majority of analyses were performed using the Vegan^80^ package including rarefaction curves (rarefy() function) and beta diversity analysis. For beta diversity, non-metric distance scaling (NMDS) was performed using two different distance measures in metamds() function: (i) Bray-Curtis and (ii) unweighted UniFrac^81^. For Unifrac distances, the Phyloseq^82^ package was employed. Additionally, ordiellipse() function was used to draw the 95% confidence interval of standard errors on NMDS plots. Analysis of variance (PERMANOVA) against sources of variations (groups in this study) was performed using Vegan’s adonis() function against the distance matrices as described above, while aov() was used to calculate pair-wise ANOVA. To give an account of abundant OTUs, proportional abundances of top *N* OTUs were plotted using a custom script. These scripts and workflows are available at Dr. Umer Z. Ijaz webpage^83^.

### Ethical approval

Ethical approval for the use of stool samples (SysMedIBD; European Union Seventh Framework Programme (FP7) under grant agreement no. 305564) was granted by NRES Committee North West - Liverpool East, reference (15/NW/0045).

## Supplementary information

Supplementary Information and dataset

Supplementary Table S1

Supplementary Table S2

Supplementary Table S3

## Data Availability

The datasets are available at the European Nucleotide Archive^84^ under study accession numbers ERP111304 and ERP111352. The first includes the reads produced during the testing phase of the 5 primer sets, when mock community and spiked stool sample were used as template. The second includes the amplicons produced with 18S rRNA and ITS2 primers, in which the templates were stool samples from the SysMed IBD project. Raw data related to gene quantification experiments can be found in the platform Figshare^85^.

## References

[1] Richard ML, Lamas B, Liguori G, Hoffmann TW, Sokol H (2014). Gut fungal microbiota: the Yin and Yang of inflammatory bowel disease. Inflammatory Bowel Diseases.

[2] Liguori G (2015). Fungal dysbiosis in mucosa-associated microbiota of Crohn’s disease patients. Journal of Crohn’s and Colitis.

[3] Hoarau G (2016). Bacteriome and mycobiome interactions underscore microbial dysbiosis in familial Crohn’s disease. Mbio.

[4] Sokol H (2017). Fungal microbiota dysbiosis in IBD. Gut.

[5] Tedersoo L, Lindahl B (2016). Fungal identification biases in microbiome projects. Environmental Microbiology Reports.

[6] De Filippis, F., Laiola, M., Blaiotta, G. & Ercolini, D. Different amplicon targets for sequencing-based studies of fungal diversity. *Applied and Environmental Microbiology* **83**, 10.1128/AEM.00905-17 (2017). 10.1128/AEM.00905-17 PMC5561290 28625991

[7] Huseyin CE, Rubio RC, O’Sullivan O, Cotter PD, Scanlan PD (2017). The fungal frontier: a comparative analysis of methods used in the study of the human gut mycobiome. Frontiers in Microbiology.

[8] Wesolowska-Andersen A (2014). Choice of bacterial DNA extraction method from fecal material influences community structure as evaluated by metagenomic analysis. Microbiome.

[9] Guo F, Zhang T (2013). Biases during DNA extraction of activated sludge samples revealed by high throughput sequencing. Applied Microbiology and Biotechnology.

[10] Kim D (2017). Optimizing methods and dodging pitfalls in microbiome research. Microbiome.

[11] Moore, D., Robson, G. D. & Trinci, A. P. *21st century guidebook to fungi* (Cambridge University Press, 2011).

[12] Erwig LP, Gow NA (2016). Interactions of fungal pathogens with phagocytes. Nature Reviews Microbiology.

[13] Karakousis A, Tan L, Ellis D, Alexiou H, Wormald P (2006). An assessment of the efficiency of fungal DNA extraction methods for maximizing the detection of medically important fungi using PCR. Journal of Microbiological Methods.

[14] Fredricks DN, Smith C, Meier A (2005). Comparison of six DNA extraction methods for recovery of fungal DNA as assessed by quantitative PCR. Journal of Clinical Microbiology.

[15] Saunders, G. C. & Rossi, J. M. DNA extraction. In Keer, J. T. & Birch, L. (eds) *Essentials of Nucleic Acid Analysis: A Robust Approach*, chap. 4, 59–82, 10.1039/9781847558213-00059 (Royal Society of Chemistry, 2008).

[16] Scanlan PD, Marchesi JR (2008). Micro-eukaryotic diversity of the human distal gut microbiota: qualitative assessment using culture-dependent and -independent analysis of faeces. The ISME Journal.

[17] Ukhanova M (2014). Effects of almond and pistachio consumption on gut microbiota composition in a randomised cross-over human feeding study. British Journal of Nutrition.

[18] Ott SJ (2008). Fungi and inflammatory bowel diseases: alterations of composition and diversity. Scandinavian Journal of Gastroenterology.

[19] David LA (2014). Diet rapidly and reproducibly alters the human gut microbiome. Nature.

[20] Li Q (2014). Dysbiosis of gut fungal microbiota is associated with mucosal inflammation in Crohn’s disease. Journal of Clinical Gastroenterology.

[21] Chehoud C (2015). Fungal signature in the gut microbiota of pediatric patients with inflammatory bowel disease. Inflammatory Bowel Diseases.

[22] Heisel T (2015). Complementary amplicon-based genomic approaches for the study of fungal communities in humans. PLoS One.

[23] Qiu X (2015). Changes in the composition of intestinal fungi and their role in mice with dextran sulfate sodium-induced colitis. Scientific Reports.

[24] Chen Y (2011). Correlation between gastrointestinal fungi and varying degrees of chronic hepatitis B virus infection. Diagnostic Microbiology and Infectious Disease.

[25] Dollive S (2012). A tool kit for quantifying eukaryotic rRNA gene sequences from human microbiome samples. Genome Biology.

[26] Iliev ID (2012). Interactions between commensal fungi and the C-type lectin receptor Dectin-1 influence colitis. Science.

[27] Pandey PK (2012). Molecular typing of fecal eukaryotic microbiota of human infants and their respective mothers. Journal of Biosciences.

[28] Hallen-Adams HE, Kachman SD, Kim J, Legge RM, Martnez I (2015). Fungi inhabiting the healthy human gastrointestinal tract: a diverse and dynamic community. Fungal Ecology.

[29] Luan C (2015). Dysbiosis of fungal microbiota in the intestinal mucosa of patients with colorectal adenomas. Scientific Reports.

[30] Tang J, Iliev ID, Brown J, Underhill DM, Funari VA (2015). Mycobiome: approaches to analysis of intestinal fungi. Journal of Immunological Methods.

[31] Schoch CL (2012). Nuclear ribosomal internal transcribed spacer (ITS) region as a universal DNA barcode marker for Fungi. Proceedings of the National Academy of Sciences.

[32] Nilsson RH (2008). variability in the kingdom Fungi as expressed in the international sequence databases and its implications for molecular species identification. Evolutionary Bioinformatics.

[33] Bazzicalupo AL, Bálint M, Schmitt I (2013). Comparison of ITS1 and ITS2 rDNA in 454 sequencing of hyperdiverse fungal communities. Fungal Ecology.

[34] Tedersoo Leho, Anslan Sten, Bahram Mohammad, Põlme Sergei, Riit Taavi, Liiv Ingrid, Kõljalg Urmas, Kisand Veljo, Nilsson Henrik, Hildebrand Falk, Bork Peer, Abarenkov Kessy (2015). Shotgun metagenomes and multiple primer pair-barcode combinations of amplicons reveal biases in metabarcoding analyses of fungi. MycoKeys.

[35] Bruns TD, Shefferson RP (2004). Evolutionary studies of ectomycorrhizal fungi: recent advances and future directions. Canadian Journal of Botany.

[36] O’Brien HE, Parrent JL, Jackson JA, Moncalvo J-M, Vilgalys R (2005). Fungal community analysis by large-scale sequencing of environmental samples. Applied and Environmental Microbiology.

[37] Brown SP, Rigdon-Huss AR, Jumpponen A (2014). Analyses of ITS and LSU gene regions provide congruent results on fungal community responses. Fungal Ecology.

[38] Mueller, R. C., Gallegos-Graves, L. V. & Kuske, C. R. A new fungal large subunit ribosomal RNA primer for high-throughput sequencing surveys. *FEMS Microbiology Ecology* **92**, 10.1093/femsec/fiv153 (2016). 10.1093/femsec/fiv153 26656064

[39] Stoeck T (2010). Multiple marker parallel tag environmental DNA sequencing reveals a highly complex eukaryotic community in marine anoxic water. Molecular Ecology.

[40] Gardes M, Bruns TD (1993). ITS primers with enhanced specificity for basidiomycetes-application to the identification of mycorrhizae and rusts. Molecular Ecology.

[41] White, T. J., Bruns, T., Lee, S. & Taylor, J. Amplification and direct sequencing of fungal ribosomal RNA genes for phylogenetics. In Innis, M. A., Gelfand, D. H., Sninsky, J. J. & White, T. J. (eds) *PCR protocols: a guide to methods and applications*, chap. 38, 315–322 (Academic Press, San Diego, CA, 1990).

[42] Liu CM (2012). FungiQuant: a broad-coverage fungal quantitative real-time PCR assay. BMC Microbiology.

[43] Illumina. Technical Note: DNA Sequencing. Nextera®Library Validation and Cluster Density Optimization. Tech. Rep., Illumina (Pub. No. 770-2013-003, 23 November 2014).

[44] Zalar P (2008). Redefinition of *Aureobasidium pullulans* and its varieties. Studies in Mycology.

[45] Sugita T, Tajima M, Amaya M, Tsuboi R, Nishikawa A (2004). Genotype analysis of *Malassezia restricta* as the major cutaneous flora in patients with atopic dermatitis and healthy subjects. Microbiology and Immunology.

[46] D’Amore R (2016). A comprehensive benchmarking study of protocols and sequencing platforms for 16S rRNA community profiling. BMC Genomics.

[47] Thomas V, Clark J, Doré J (2015). Fecal microbiota analysis: an overview of sample collection methods and sequencing strategies. Future Microbiology.

[48] Griffiths LJ (2006). Comparison of DNA extraction methods for *Aspergillus fumigatus* using real-time PCR. Journal of Medical Microbiology.

[49] Nawrot U (2010). Comparison of the utility of five commercial kits for extraction of DNA from *Aspergillus fumigatus* spores. Acta Biochimica Polonica.

[50] White PL (2010). *Aspergillus* PCR: one step closer to standardization. Journal of Clinical Microbiology.

[51] Bjørnsgaard Aas A, Davey ML, Kauserud H (2017). ITS all right mama: investigating the formation of chimeric sequences in the ITS 2 region by DNA metabarcoding analyses of fungal mock communities of different complexities. Molecular Ecology Resources.

[52] Guého-Kellermann Eveline, Boekhout Teun, Begerow Dominik (2010). Biodiversity, Phylogeny and Ultrastructure. Malassezia and the Skin.

[53] STRATEC Molecular GmbH, Berlin, Germany. *User manual PSP*®*Spin Stool DNA Kit*/*User manual PSP*®*Spin Stool DNA Plus Kit* (2013).

[54] QIAGEN®. *QIAamp*®*Fast DNA Stool Mini Handbook 03*/*2014* (2014).

[55] Roche. *LightCycler*®*480 Instrument Operator*’*s Manual*, *Software Version 1*.*5* (2008).

[56] Rasmussen Randy (2001). Quantification on the LightCycler. Rapid Cycle Real-Time PCR.

[57] R Core Team. *R: A Language and Environment for Statistical Computing*. R Foundation for Statistical Computing, Vienna, Austria (2017).

[58] Martin M (2011). Cutadapt removes adapter sequences from high-throughput sequencing reads. EMBnet. journal.

[59] Joshi, N. & Fass, J. Sickle: A sliding-window, adaptive, quality-based trimming tool for FastQ files (Version 1.33)[Software]. link to Version 1.2, https://github.com/najoshi/sickle/releases/tag/v1.2, Online; accessed 30 January 2019 (2011).

[60] Nikolenko Sergey I, Korobeynikov Anton I, Alekseyev Max A (2013). BayesHammer: Bayesian clustering for error correction in single-cell sequencing. BMC Genomics.

[61] Schirmer M (2015). Insight into biases and sequencing errors for amplicon sequencing with the Illumina MiSeq platform. Nucleic Acids Research.

[62] Nurk S (2013). Assembling single-cell genomes and mini-metagenomes from chimeric MDA products. Journal of Computational Biology.

[63] Zhang J, Kobert K, Flouri T, Stamatakis A (2013). PEAR: a fast and accurate Illumina Paired-End reAd mergeR. Bioinformatics.

[64] Mahé F, Rognes T, Quince C, de Vargas C, Dunthorn M (2015). Swarm v2: highly-scalable and high-resolution amplicon clustering. PeerJ.

[65] Bengtsson-Palme J (2013). Improved software detection and extraction of ITS1 and ITS2 from ribosomal ITS sequences of fungi and other eukaryotes for analysis of environmental sequencing data. Methods in Ecology and Evolution.

[66] Edgar RC, Haas BJ, Clemente JC, Quince C, Knight R (2011). UCHIME improves sensitivity and speed of chimera detection. Bioinformatics.

[67] Altschul SF, Gish W, Miller W, Myers EW, Lipman DJ (1990). Basic local alignment search tool. Journal of Molecular Biology.

[68] Caporaso JG (2010). QIIME allows analysis of high-throughput community sequencing data. Nature Methods.

[69] Quast C (2012). The SILVA ribosomal RNA gene database project: improved data processing and web-based tools. Nucleic Acids Research.

[70] Liu K-L, Porras-Alfaro A, Kuske CR, Eichorst SA, Xie G (2012). Accurate, rapid taxonomic classification of fungal large-subunit rRNA genes. Applied and Environmental Microbiology.

[71] Kõljalg U (2013). Towards a unified paradigm for sequence-based identification of fungi. Molecular Ecology.

[72] Bokulich NA (2013). Quality-filtering vastly improves diversity estimates from Illumina amplicon sequencing. Nature Methods.

[73] Caporaso JG (2009). PyNAST: a flexible tool for aligning sequences to a template alignment. Bioinformatics.

[74] Price MN, Dehal PS, Arkin AP (2010). FastTree 2–approximately maximum-likelihood trees for large alignments. PLoS One.

[75] NCBI. Nucleotide Blast (blastn), https://blast.ncbi.nlm.nih.gov (Online; accessed August-September 2018).

[76] Walters WA (2011). Primerprospector: de novo design and taxonomic analysis of barcoded polymerase chain reaction primers. Bioinformatics.

[77] NCBI. Nucleotide database, https://blast.ncbi.nlm.nih.gov (Online; accessed February 2019).

[78] Kumar S, Stecher G, Li M, Knyaz C, Tamura K (2018). MEGA X: Molecular Evolutionary Genetics Analysis across Computing Platforms. Molecular Biology and Evolution.

[79] Larkin MA (2007). Clustal W and Clustal X version 2.0. Bioinformatics.

[80] Oksanen, J. *et al*. *vegan: Community Ecology Package*, R package version 2.5-1 (2018).

[81] Lozupone C, Knight R (2005). UniFrac: a new phylogenetic method for comparing microbial communities. Applied and Environmental Microbiology.

[82] McMurdie PJ, Holmes S (2013). phyloseq: an R package for reproducible interactive analysis and graphics of microbiome census data. PLoS One.

[83] Ijaz, U. Z. Dr Umer Zeeshan Ijaz bioinformatic resources, http://userweb.eng.gla.ac.uk/umer.ijaz#bioinformatics (Online; accessed 8 October 2018).

[84] EMBL-EBI. European Nucleotide Archive (ENA), https://www.ebi.ac.uk/ena (Online; accessed 8 October 2018).

[85] Frau, A. *et al*. qPCR raw data relevant to this study are available in Figshare, 10.6084/m9.figshare.8138018 (Online; accessed 16 May 2019).

[86] Ihrmark K (2012). New primers to amplify the fungal ITS2 region–evaluation by 454-sequencing of artificial and natural communities. FEMS Microbiology Ecology.

