# Supplementary material for: DNA extraction and amplicon production strategies deeply inf luence the outcome of gut mycobiome studies
Source: Sci Rep. 2019 Jun 27;9:9328. doi: 10.1038/s41598-019-44974-x (PMC6597572; doi:10.1038/s41598-019-44974-x)
Supplement: Supplementary file 1 — Supplementary Information and dataset [file 41598_2019_44974_MOESM1_ESM.pdf]

DNA extraction and amplicon production strategies deeply influence the outcome of gut mycobiome studies

Alessandra Frau[1,*], John G Kenny[2], Luca Lenzi[2], Barry J Campbell[1], Umer Z Ijaz[3], Carrie A Duckworth[1], Michael D Burkitt[1,4], Neil Hall[5], Jim Anson[6], Alistair C Darby[2], and Christopher S J Probert[1,*]

[1]Gastroenterology Research Unit, Department of Cellular & Molecular Physiology, Institute of Translational Medicine, University of Liverpool, Ashton Street, Liverpool, L69 3GE, UK
[2]Centre for Genomic Research (CGR), University of Liverpool, Crown Street, Liverpool, L69 7ZB, UK
[3]School of Engineering, University of Glasgow, Oakfield Avenue, Glasgow, G12 8LT, UK
[4]Division of Diabetes, Endocrinology and Gastroenterology, University of Manchester, Dover Street, Manchester, M13 9PT, UK
[5]Earlham Institute, Colney Ln, Norwich, NR4 7UZ, UK
[6]Liverpool Clinical Laboratories Directorate of Infection and Immunity, Royal Liverpool and Broadgreen University Hospitals NHS Trust, Prescot Street, Liverpool, L7 8XP, UK
*afrau@liverpool.ac.uk; mdcsjp@liverpool.ac.uk

Supplementary information and dataset

- **Supplementary Figure S1**
- **Supplementary Figure S2**
- **Supplementary Figure S2**
- **Supplementary Data S1**

Supplementary Figure S1. Fragment analyser trace of the pool of amplicons of the trial plate were the 5 primer sets were tested before (A) and after (B) size selection. The numbers on top of the peaks represents their length in base pairs (bp), shown also in the *x* axis. The *y* axis reports fluorescent units (FU).

A. Before size selection

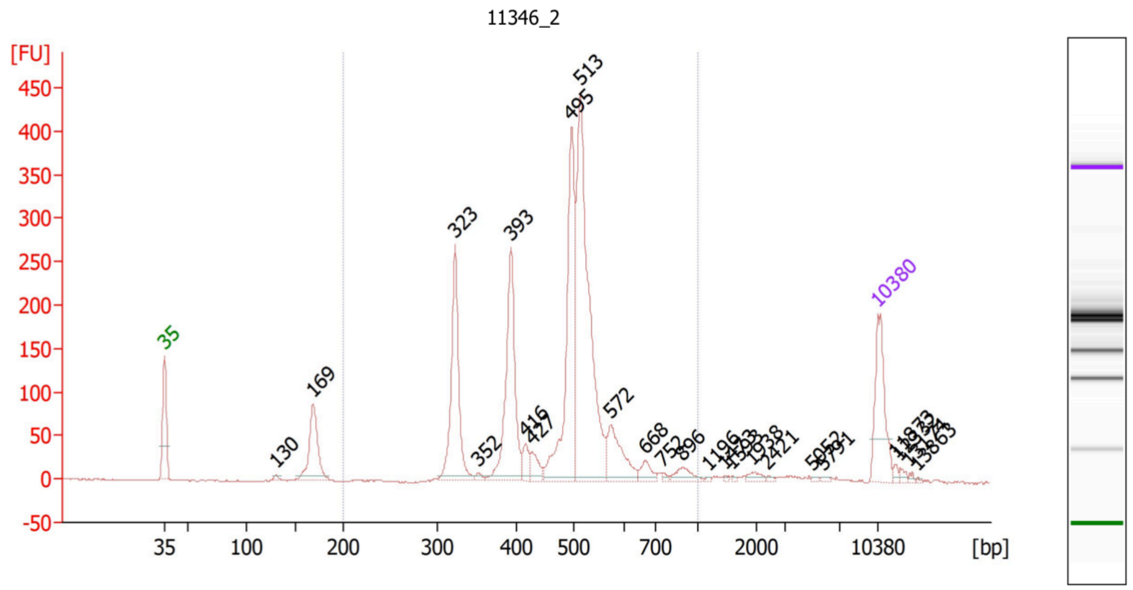

B. After size selection

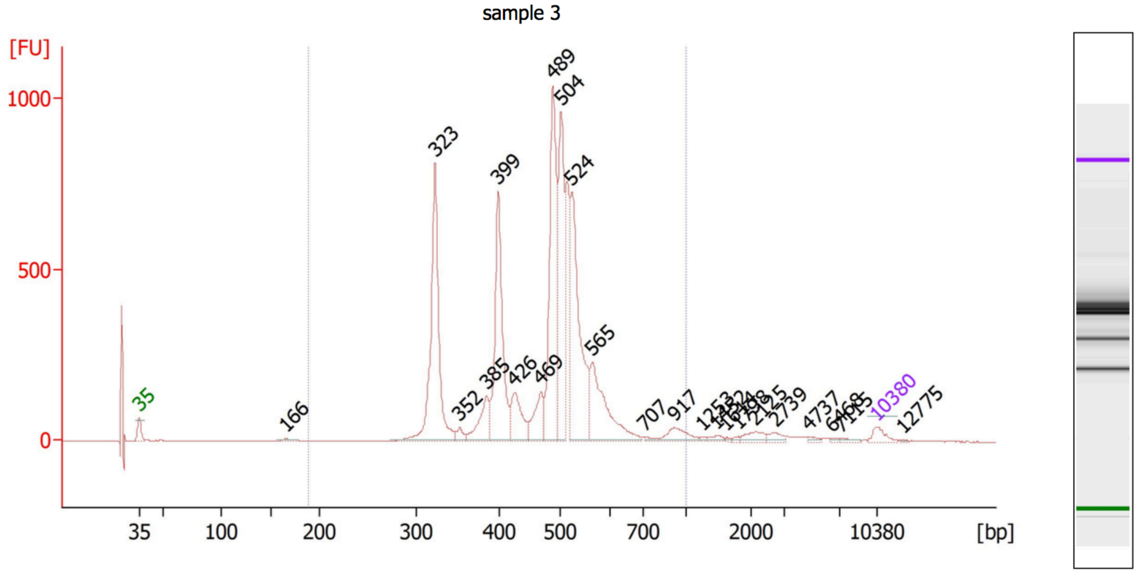

Supplementary Figure S2: fragment analyser traces (18S rRNA and ITS2 amplions from stool samples from donors). Below is the key of the Sample # and its position in the plate. Afterwards, each page shows a trace for each of the samples, in these the plate position is indicated.

Sample #	Plate position
D-004-18S	A2
D-008-18S	A5
D-009-18S	A6
D-011-18S	A7
D-013-18S	A9
D-022-18S	B3
D-024-18S	B5
D-027-18S	B7
D-028-18S	B8
D-030-18S	B10
D-036-18S	C1
D-043-18S	C3
D-045-18S	C4
D-050-18S	C7
D-054-18S	C9
D-055-18S	C10
D-057-18S	C12
D-068-18S	D4
D-071-18S	D5
D-076-18S	D9
D-079-18S	D11
D-088-18S	E5
D-090-18S	E7
D-094-18S	E9
D-004-ITS2	F10
D-008-ITS2	F11
D-009-ITS2	F12
D-011-ITS2	G1
D-013-ITS2	G2
D-022-ITS2	G3
D-024-ITS2	G4
D-027-ITS2	G5
D-028-ITS2	G6
D-030-ITS2	G7
D-036-ITS2	G8
D-043-ITS2	G9
D-045-ITS2	G10
D-050-ITS2	G11
D-054-ITS2	G12
D-055-ITS2	H1
D-057-ITS2	H2
D-068-ITS2	H3
D-071-ITS2	H4
D-076-ITS2	H5
D-079-ITS2	H6
D-088-ITS2	H7
D-090-ITS2	H8
D-094-ITS2	H9

Sample: ID13507_2
Well Location: A2
Created: Tuesday, June 27, 2017 12:18:19 PM

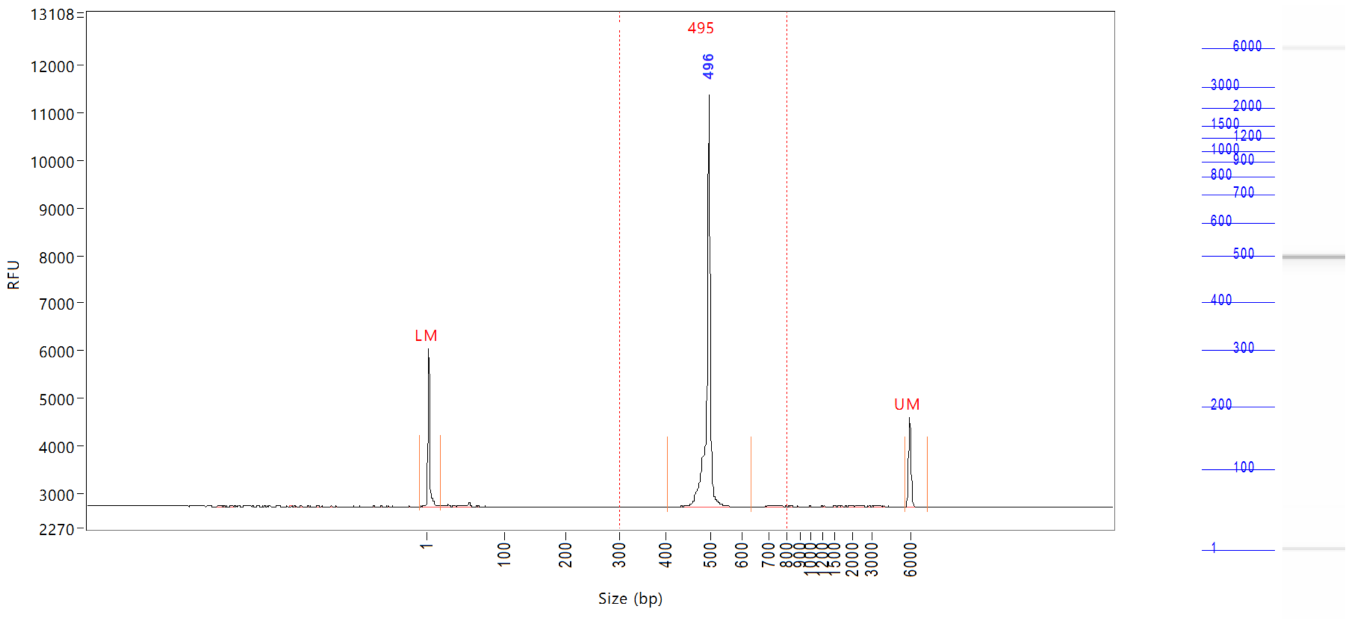

Peak	Size (bp)	Conc. (ng/uL)	From (bp)	To (bp)	Avg. Size (bp)	CV%	RFU	Corr. Peak Area
1	1 (LM)	0.0184	0	17	1	227.98	3325	19.813
2	496	0.6539	403	637	493	2.57	8658	58.781
3	6000 (UM)	0.0065	5652	7463	5987	1.84	1881	6.998

TIC:	0.6539	ng/uL
TIM:	2.168	nmole/L
Total Conc.:	0.6963	ng/uL

Smear Analysis	300 bp to 800 bp	0.6607 ng/ul	94.9 %Total	2.198 nmole/L	495 Avg. Size (b.p.)	5.24 %CV

Sample Peak Width (sec): 50 Sample Min Peak Height: 25 Sample Baseline V to V?: Y Sample Baseline V to V pts: 3
Sample Filter: Binomial # of Pts for Filter: 3 Sample Start Region (min): 0 Sample End Region (min): 50
Manual Baseline Start (min): 10 Manual Baseline End (min): 48
Marker Peak Width (sec): 5 Marker Min Peak Height: 200 Marker Baseline V to V?: Y Marker Baseline V to V pts: 3
Lower Marker Selection: First Peak > 200 RFU Upper Marker Selection: Last Peak > 200 RFU
Ladder Size (bp): 1, 100, 200, 300, 400, 500, 600, 700, 800, 900, 1000, 1200, 1500, 2000, 3000, 6000
Quantification Using: Ladder Final Concentration (ng/uL): 0.0830 Dilution Factor: 12.0

 Copyright 2015 Advanced Analytical Technologies, Inc.

Sample: ID13507_5
Well Location: A5
Created: Tuesday, June 27, 2017 12:18:19 PM

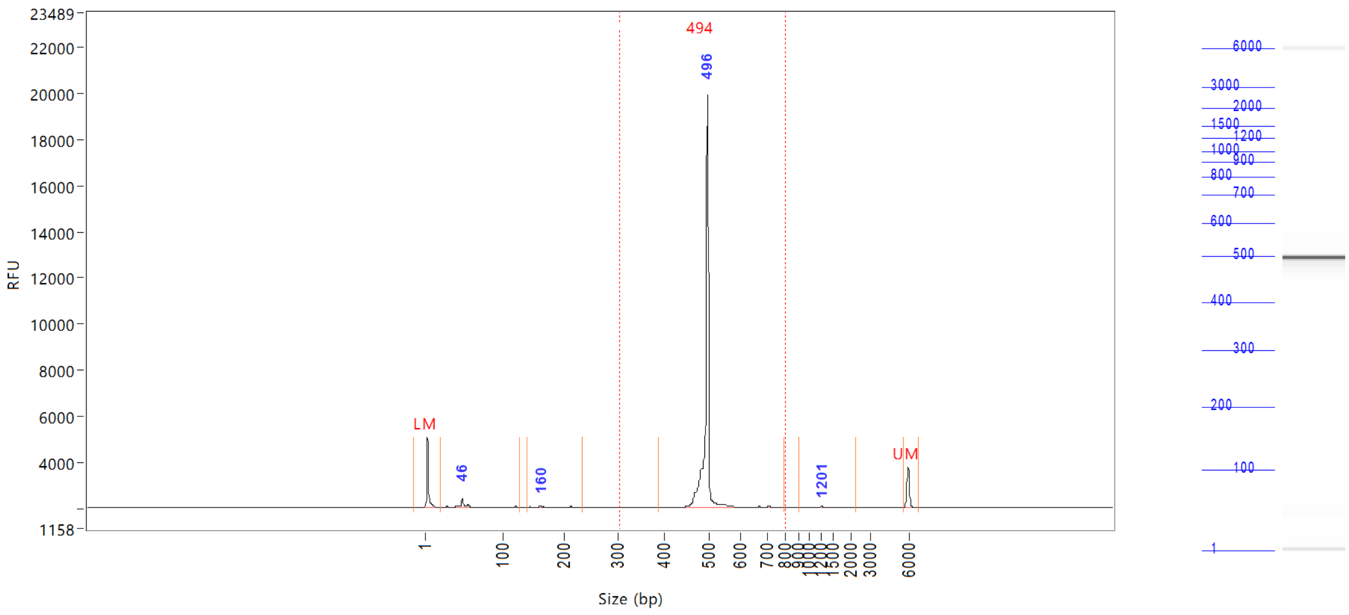

Peak	Size (bp)	Conc. (ng/uL)	From (bp)	To (bp)	Avg. Size (bp)	CV%	RFU	Corr. Peak Area
1	1 (LM)	0.0184	0	19	1	191.99	3051	18.136
2	46	0.0709	19	126	52	39.61	397	5.832
3	160	0.0142	139	233	173	14.06	49	1.171
4	496	1.2671	387	794	495	5.03	17852	104.262
5	1201	0.0169	900	2294	1500	24.95	47	1.387
6	6000 (UM)	0.0066	5598	6824	5969	1.79	1726	6.476

TIC:	1.3691	ng/uL
TIM:	6.881	nmole/L
Total Conc.:	1.3936	ng/uL

Smear Analysis 300 bp to 800 bp 1.2724 ng/ul 91.3 %Total 4.237 nmole/L 494 Avg. Size (b.p.) 5.44 %CV

```
Sample Peak Width (sec): 50   Sample Min Peak Height: 25    Sample Baseline V to V?: Y    Sample Baseline V to V pts: 3
Sample Filter: Binomial       # of Pts for Filter: 3        Sample Start Region (min): 0  Sample End Region (min): 50
Manual Baseline Start (min): 10     Manual Baseline End (min): 48
Marker Peak Width (sec): 5    Marker Min Peak Height: 200   Marker Baseline V to V?: Y    Marker Baseline V to V pts: 3
Lower Marker Selection: First Peak > 200 RFU                Upper Marker Selection: Last Peak > 200 RFU
Ladder Size (bp): 1, 100, 200, 300, 400, 500, 600, 700, 800, 900, 1000, 1200, 1500, 2000, 3000, 6000
Quantification Using: Ladder          Final Concentration (ng/uL): 0.0830          Dilution Factor: 12.0
```


Copyright 2015 Advanced Analytical Technologies, Inc.

Sample: ID13507_6
Well Location: A6
Created: Tuesday, June 27, 2017 12:18:19 PM

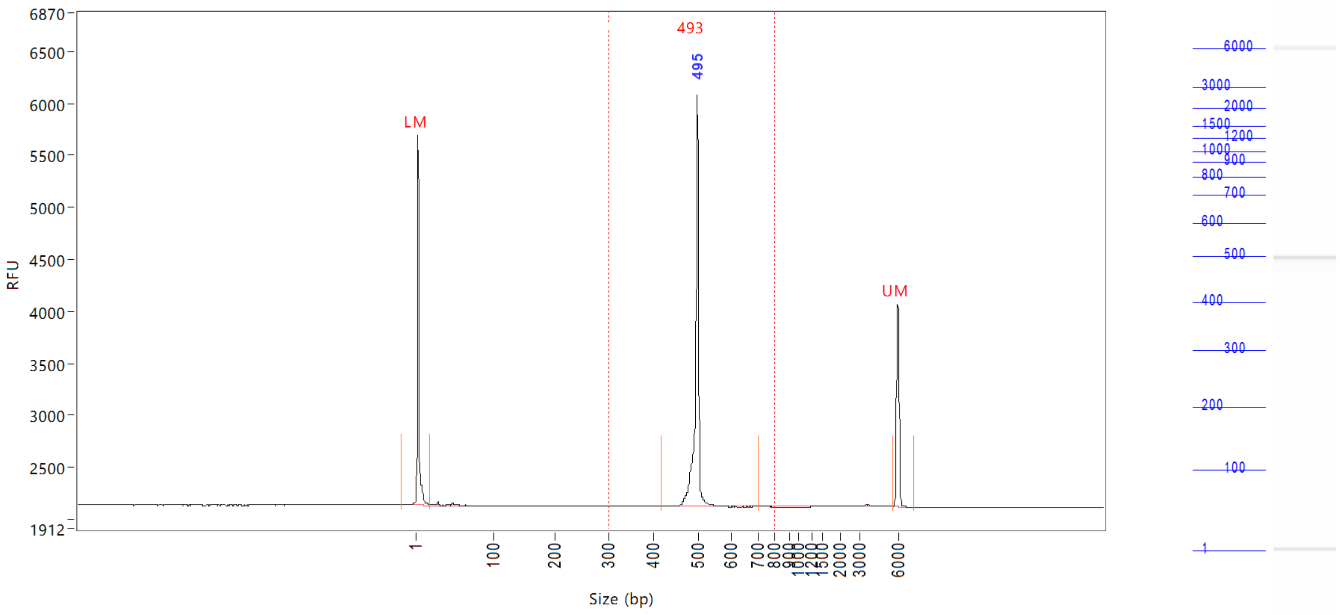

Peak	Size (bp)	Conc. (ng/uL)	From (bp)	To (bp)	Avg. Size (bp)	CV%	RFU	Corr. Peak Area
1	1 (LM)	0.0184	0	17	1	211.94	3563	20.454
2	495	0.2548	417	707	493	1.81	3950	23.649
3	6000 (UM)	0.0064	5652	7303	5983	1.60	1952	7.161

TIC:	0.2548	ng/uL
TIM:	0.846	nmole/L
Total Conc.:	0.2681	ng/uL

Smear Analysis 300 bp to 800 bp 0.2549 ng/ul 95.1 %Total 0.852 nmole/L 493 Avg. Size (b.p.) 1.95 %CV

Sample Peak Width (sec): 50 Sample Min Peak Height: 25 Sample Baseline V to V?: Y Sample Baseline V to V pts: 3
Sample Filter: Binomial # of Pts for Filter: 3 Sample Start Region (min): 0 Sample End Region (min): 50
Manual Baseline Start (min): 10 Manual Baseline End (min): 48
Marker Peak Width (sec): 5 Marker Min Peak Height: 200 Marker Baseline V to V?: Y Marker Baseline V to V pts: 3
Lower Marker Selection: First Peak > 200 RFU Upper Marker Selection: Last Peak > 200 RFU
Ladder Size (bp): 1, 100, 200, 300, 400, 500, 600, 700, 800, 900, 1000, 1200, 1500, 2000, 3000, 6000
Quantification Using: Ladder Final Concentration (ng/uL): 0.0830 Dilution Factor: 12.0

 Copyright 2015 Advanced Analytical Technologies, Inc.

Sample: ID13507_7
Well Location: A7
Created: Tuesday, June 27, 2017 12:18:19 PM

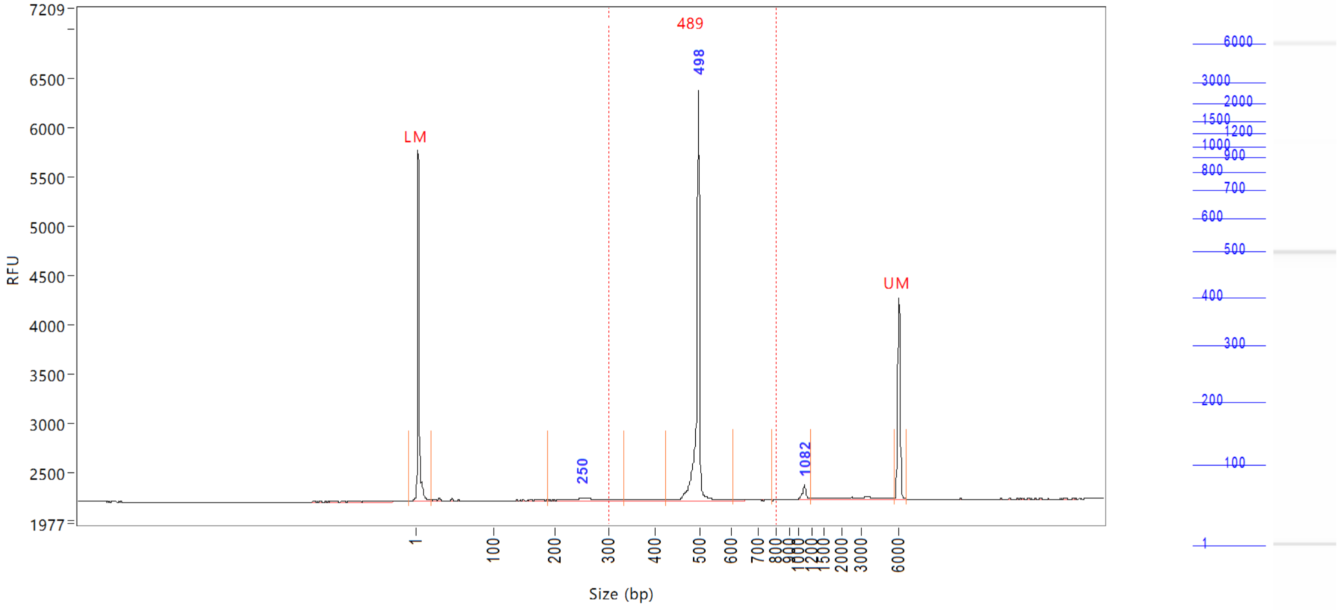

Peak	Size (bp)	Conc. (ng/uL)	From (bp)	To (bp)	Avg. Size (bp)	CV%	RFU	Corr. Peak Area
1	1 (LM)	0.0184	0	20	2	182.04	3564	21.162
2	250	0.0241	189	334	258	13.65	36	2.317
3	498	0.2485	423	605	494	2.41	4162	23.856
4	1082	0.0115	780	1189	1056	6.56	155	1.106
5	6000 (UM)	0.0066	5679	6612	5990	1.67	2043	7.586

TIC:	0.2841	ng/uL
TIM:	0.998	nmole/L
Total Conc.:	0.3451	ng/uL

Smear Analysis	300 bp to 800 bp	0.2605 ng/ul	75.5 %Total	0.876 nmole/L	489 Avg. Size (b.p.)	6.95 %CV

```
Sample Peak Width (sec): 50   Sample Min Peak Height: 25    Sample Baseline V to V?: Y    Sample Baseline V to V pts: 3
Sample Filter: Binomial       # of Pts for Filter: 3        Sample Start Region (min): 0  Sample End Region (min): 50
Manual Baseline Start (min): 10     Manual Baseline End (min): 48
Marker Peak Width (sec): 5    Marker Min Peak Height: 200   Marker Baseline V to V?: Y    Marker Baseline V to V pts: 3
Lower Marker Selection: First Peak > 200 RFU                Upper Marker Selection: Last Peak > 200 RFU
Ladder Size (bp): 1, 100, 200, 300, 400, 500, 600, 700, 800, 900, 1000, 1200, 1500, 2000, 3000, 6000
Quantification Using: Ladder          Final Concentration (ng/uL): 0.0830         Dilution Factor: 12.0
```

 Copyright 2015 Advanced Analytical Technologies, Inc.

Sample: ID13507_9
Well Location: A9
Created: Tuesday, June 27, 2017 12:18:19 PM

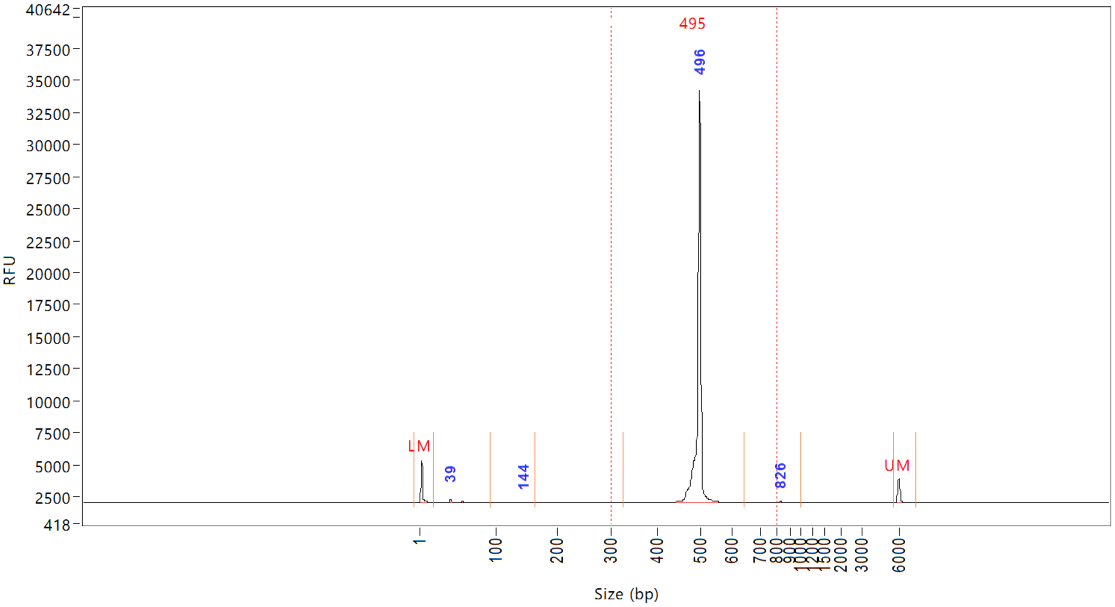

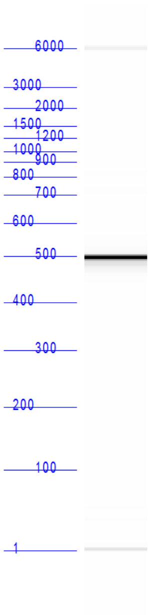

Peak	Size (bp)	Conc. (ng/uL)	From (bp)	To (bp)	Avg. Size (bp)	CV%	RFU	Corr. Peak Area
1	1 (LM)	0.0184	0	18	1	204.98	3320	19.783
2	39	0.0429	18	92	44	32.46	260	3.853
3	144	0.0105	92	162	134	14.09	44	0.945
4	496	2.1907	327	641	492	3.46	32172	196.627
5	826	0.0381	641	1028	762	10.87	82	3.420
6	6000 (UM)	0.0064	5625	7356	5978	1.89	1838	6.848

TIC:	2.2822	ng/uL
TIM:	9.267	nmole/L
Total Conc.:	2.3265	ng/uL

Smear Analysis 300 bp to 800 bp 2.2205 ng/ul 95.4 %Total 7.386 nmole/L 495 Avg. Size (b.p.) 6.16 %CV

Sample Peak Width (sec): 50 Sample Min Peak Height: 25 Sample Baseline V to V?: Y Sample Baseline V to V pts: 3
Sample Filter: Binomial # of Pts for Filter: 3 Sample Start Region (min): 0 Sample End Region (min): 50
Manual Baseline Start (min): 10 Manual Baseline End (min): 48
Marker Peak Width (sec): 5 Marker Min Peak Height: 200 Marker Baseline V to V?: Y Marker Baseline V to V pts: 3
Lower Marker Selection: First Peak > 200 RFU Upper Marker Selection: Last Peak > 200 RFU
Ladder Size (bp): 1, 100, 200, 300, 400, 500, 600, 700, 800, 900, 1000, 1200, 1500, 2000, 3000, 6000
Quantification Using: Ladder Final Concentration (ng/uL): 0.0830 Dilution Factor: 12.0

 Copyright 2015 Advanced Analytical Technologies, Inc.

Sample: ID13507_15
Well Location: B3
Created: Tuesday, June 27, 2017 12:18:19 PM

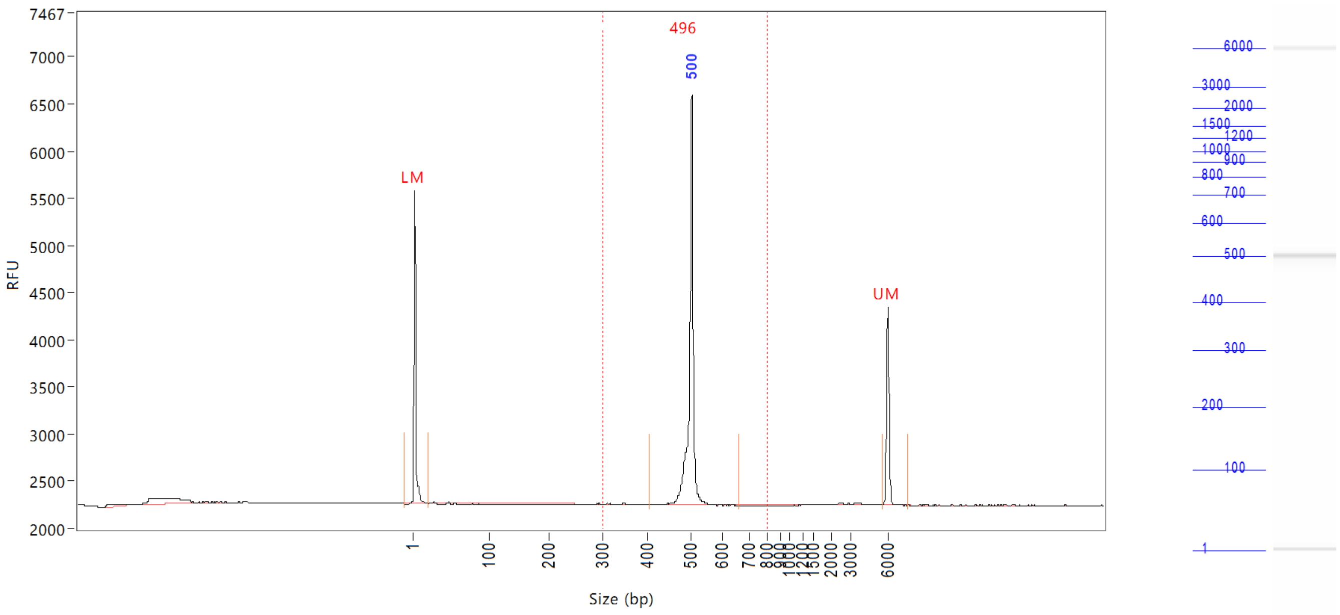

Peak	Size (bp)	Conc. (ng/uL)	From (bp)	To (bp)	Avg. Size (bp)	CV%	RFU	Corr. Peak Area
1	1 (LM)	0.0184	0	19	1	144.67	3307	18.931
2	500	0.3591	405	663	496	2.04	4337	30.847
3	6000 (UM)	0.0077	5598	7596	5983	1.94	2104	7.933

TIC:	0.3591	ng/uL
TIM:	1.182	nmole/L
Total Conc.:	0.3712	ng/uL

Smear Analysis 300 bp to 800 bp 0.3597 ng/ul 96.9 %Total 1.194 nmole/L 496 Avg. Size (b.p.) 2.41 %CV

```
Sample Peak Width (sec): 50   Sample Min Peak Height: 25    Sample Baseline V to V?: Y    Sample Baseline V to V pts: 3
Sample Filter: Binomial       # of Pts for Filter: 3        Sample Start Region (min): 0  Sample End Region (min): 50
Manual Baseline Start (min): 10     Manual Baseline End (min): 48
Marker Peak Width (sec): 5    Marker Min Peak Height: 200   Marker Baseline V to V?: Y    Marker Baseline V to V pts: 3
Lower Marker Selection: First Peak > 200 RFU                Upper Marker Selection: Last Peak > 200 RFU
Ladder Size (bp): 1, 100, 200, 300, 400, 500, 600, 700, 800, 900, 1000, 1200, 1500, 2000, 3000, 6000
Quantification Using: Ladder          Final Concentration (ng/uL): 0.0830          Dilution Factor: 12.0
```

 Copyright 2015 Advanced Analytical Technologies, Inc.

Sample: ID13507_17
Well Location: B5
Created: Tuesday, June 27, 2017 12:18:19 PM

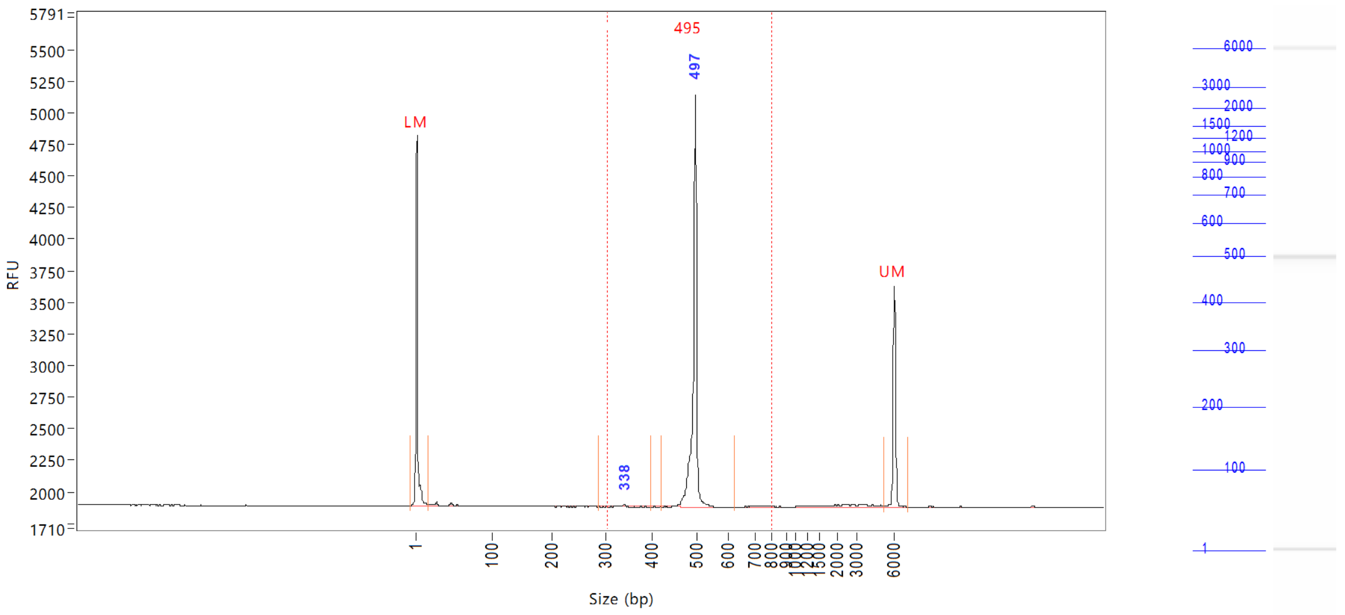

Peak	Size (bp)	Conc. (ng/uL)	From (bp)	To (bp)	Avg. Size (bp)	CV%	RFU	Corr. Peak Area
1	1 (LM)	0.0184	0	18	1	205.22	2933	17.299
2	338	0.0016	286	398	334	2.18	22	0.125
3	497	0.2652	420	623	494	1.89	3257	20.817
4	6000 (UM)	0.0071	5170	7064	5974	2.19	1751	6.667

TIC:	0.2668	ng/uL
TIM:	0.886	nmole/L
Total Conc.:	0.3056	ng/uL

Smear Analysis 300 bp to 800 bp 0.2694 ng/ul 88.2 %Total 0.896 nmole/L 495 Avg. Size (b.p.) 5.47 %CV

```
Sample Peak Width (sec): 50   Sample Min Peak Height: 25    Sample Baseline V to V?: Y    Sample Baseline V to V pts: 3
Sample Filter: Binomial       # of Pts for Filter: 3        Sample Start Region (min): 0  Sample End Region (min): 50
Manual Baseline Start (min): 10     Manual Baseline End (min): 48
Marker Peak Width (sec): 5    Marker Min Peak Height: 200   Marker Baseline V to V?: Y    Marker Baseline V to V pts: 3
Lower Marker Selection: First Peak > 200 RFU                Upper Marker Selection: Last Peak > 200 RFU
Ladder Size (bp): 1, 100, 200, 300, 400, 500, 600, 700, 800, 900, 1000, 1200, 1500, 2000, 3000, 6000
Quantification Using: Ladder          Final Concentration (ng/uL): 0.0830         Dilution Factor: 12.0
```

 Copyright 2015 Advanced Analytical Technologies, Inc.

Sample: ID13507_19
Well Location: B7
Created: Tuesday, June 27, 2017 12:18:19 PM

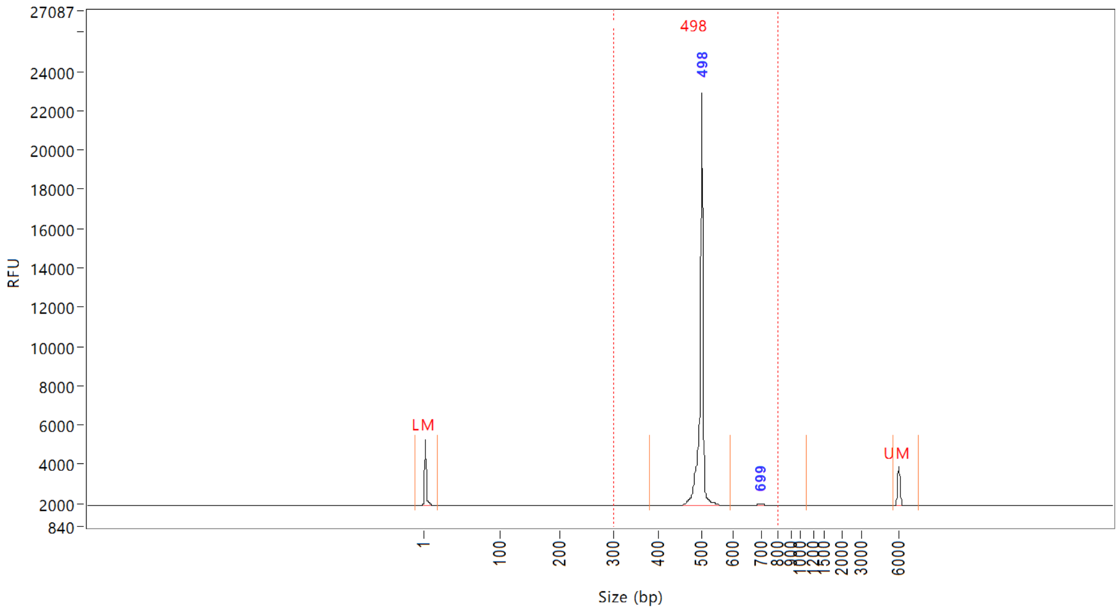

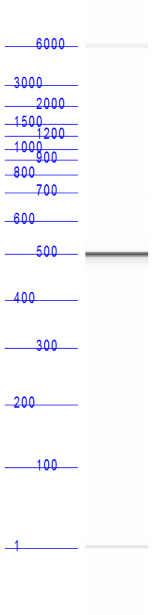

Peak	Size (bp)	Conc. (ng/uL)	From (bp)	To (bp)	Avg. Size (bp)	CV%	RFU	Corr. Peak Area
1	1 (LM)	0.0184	0	18	1	234.47	3379	20.214
2	498	1.3609	382	588	495	2.23	20990	124.811
3	699	0.0253	588	1087	713	11.13	69	2.321
4	6000 (UM)	0.0067	5598	7569	5993	2.33	1970	7.392

TIC:	1.3863	ng/uL
TIM:	4.559	nmole/L
Total Conc.:	1.4308	ng/uL

Smear Analysis	300 bp to 800 bp	1.3877 ng/ul	97.0 %Total	4.589 nmole/L	498 Avg. Size (b.p.)	5.91 %CV

Sample Peak Width (sec): 50 Sample Min Peak Height: 25 Sample Baseline V to V?: Y Sample Baseline V to V pts: 3
Sample Filter: Binomial # of Pts for Filter: 3 Sample Start Region (min): 0 Sample End Region (min): 50
Manual Baseline Start (min): 10 Manual Baseline End (min): 48
Marker Peak Width (sec): 5 Marker Min Peak Height: 200 Marker Baseline V to V?: Y Marker Baseline V to V pts: 3
Lower Marker Selection: First Peak > 200 RFU Upper Marker Selection: Last Peak > 200 RFU
Ladder Size (bp): 1, 100, 200, 300, 400, 500, 600, 700, 800, 900, 1000, 1200, 1500, 2000, 3000, 6000
Quantification Using: Ladder Final Concentration (ng/uL): 0.0830 Dilution Factor: 12.0

 Copyright 2015 Advanced Analytical Technologies, Inc.

Sample: ID13507_20
Well Location: B8
Created: Tuesday, June 27, 2017 12:18:19 PM

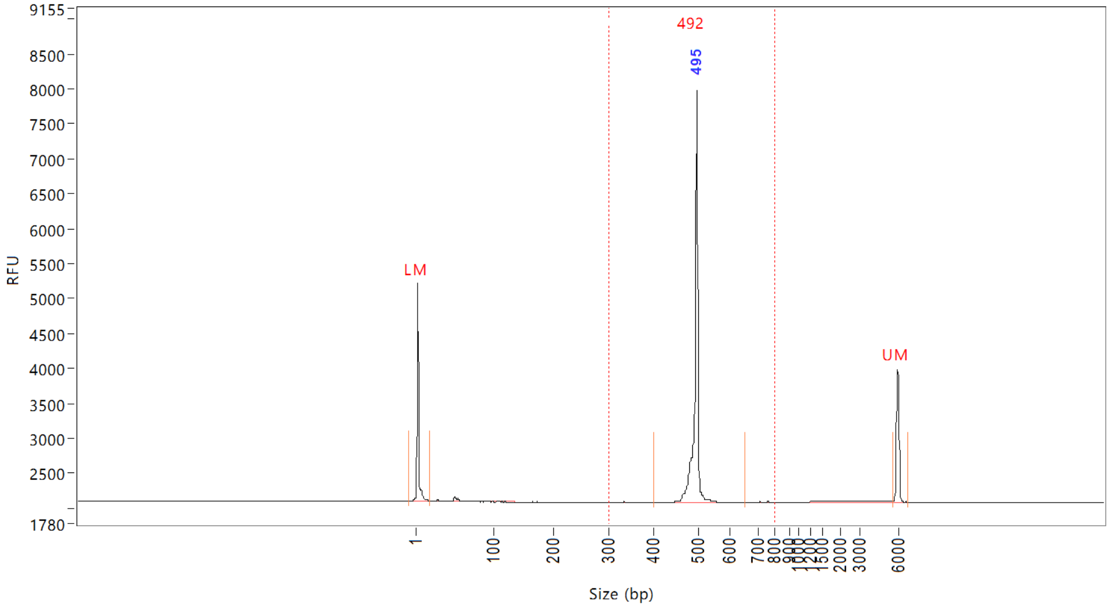

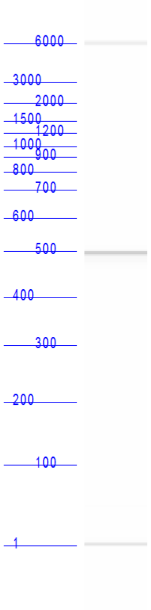

Peak	Size (bp)	Conc. (ng/uL)	From (bp)	To (bp)	Avg. Size (bp)	CV%	RFU	Corr. Peak Area
1	1 (LM)	0.0184	0	17	1	205.79	3123	18.230
2	495	0.4145	398	654	491	1.96	5889	34.288
3	6000 (UM)	0.0072	5625	6745	5976	1.80	1908	7.163

TIC:	0.4145	ng/uL
TIM:	1.379	nmole/L
Total Conc.:	0.4545	ng/uL

Smear Analysis 300 bp to 800 bp 0.4160 ng/ul 91.5 %Total 1.391 nmole/L 492 Avg. Size (b.p.) 3.52 %CV

```
Sample Peak Width (sec): 50   Sample Min Peak Height: 25    Sample Baseline V to V?: Y    Sample Baseline V to V pts: 3
Sample Filter: Binomial       # of Pts for Filter: 3        Sample Start Region (min): 0  Sample End Region (min): 50
Manual Baseline Start (min): 10     Manual Baseline End (min): 48
Marker Peak Width (sec): 5    Marker Min Peak Height: 200   Marker Baseline V to V?: Y    Marker Baseline V to V pts: 3
Lower Marker Selection: First Peak > 200 RFU                Upper Marker Selection: Last Peak > 200 RFU
Ladder Size (bp): 1, 100, 200, 300, 400, 500, 600, 700, 800, 900, 1000, 1200, 1500, 2000, 3000, 6000
Quantification Using: Ladder          Final Concentration (ng/uL): 0.0830          Dilution Factor: 12.0
```

 Copyright 2015 Advanced Analytical Technologies, Inc.

Sample: ID13507_22
Well Location: B10
Created: Tuesday, June 27, 2017 12:18:19 PM

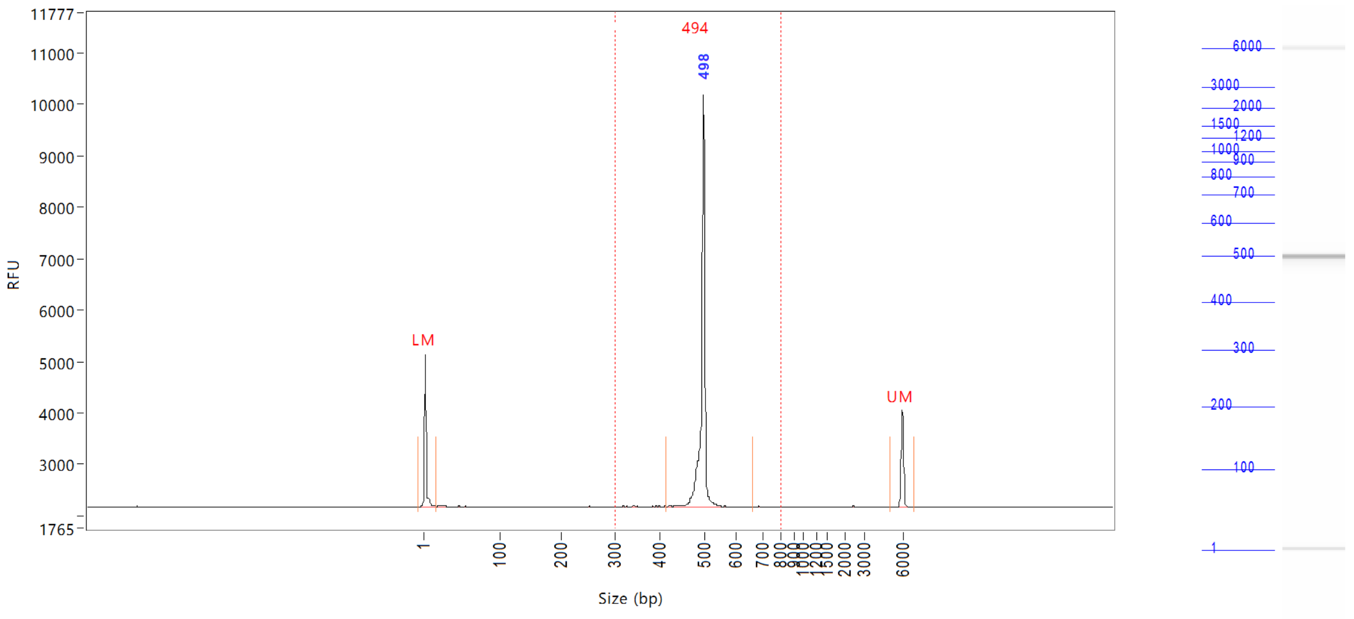

Peak	Size (bp)	Conc. (ng/uL)	From (bp)	To (bp)	Avg. Size (bp)	CV%	RFU	Corr. Peak Area
1	1 (LM)	0.0184	0	17	1	165.43	2951	17.477
2	498	0.6481	413	665	495	3.16	8002	51.391
3	6000 (UM)	0.0076	5090	6984	5987	2.06	1898	7.227
	TIC:	0.6481	ng/uL					
	TIM:	2.143	nmole/L					
	Total Conc.:	0.7069	ng/uL					

Smear Analysis	300 bp to 800 bp	0.6649 ng/ul	94.1 %Total	2.214 nmole/L	494 Avg. Size (b.p.)	6.19 %CV

Sample Peak Width (sec): 50   Sample Min Peak Height: 25   Sample Baseline V to V?: Y   Sample Baseline V to V pts: 3
Sample Filter: Binomial   # of Pts for Filter: 3   Sample Start Region (min): 0   Sample End Region (min): 50
Manual Baseline Start (min): 10   Manual Baseline End (min): 48
Marker Peak Width (sec): 5   Marker Min Peak Height: 200   Marker Baseline V to V?: Y   Marker Baseline V to V pts: 3
Lower Marker Selection: First Peak > 200 RFU   Upper Marker Selection: Last Peak > 200 RFU
Ladder Size (bp): 1, 100, 200, 300, 400, 500, 600, 700, 800, 900, 1000, 1200, 1500, 2000, 3000, 6000
Quantification Using: Ladder   Final Concentration (ng/uL): 0.0830   Dilution Factor: 12.0

 Copyright 2015 Advanced Analytical Technologies, Inc.

Sample: ID13507_25
Well Location: C1
Created: Tuesday, June 27, 2017 12:18:19 PM

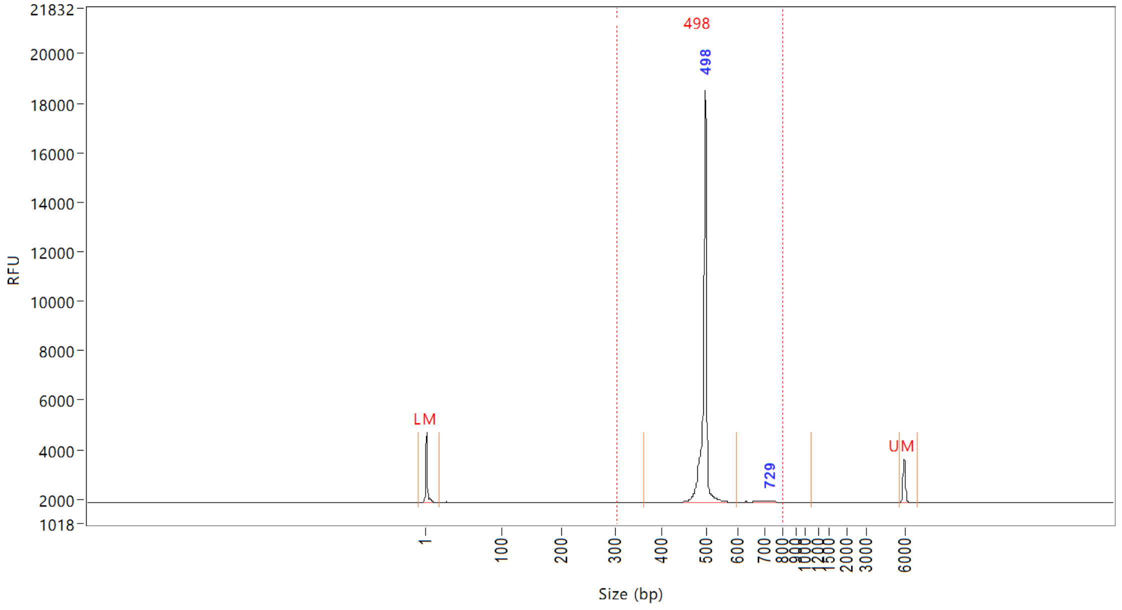

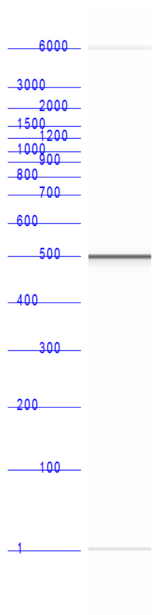

Peak	Size (bp)	Conc. (ng/uL)	From (bp)	To (bp)	Avg. Size (bp)	CV%	RFU	Corr. Peak Area
1	1 (LM)	0.0184	0	19	2	163.18	2806	16.768
2	498	1.3565	363	596	494	2.84	16641	103.200
3	729	0.0400	596	1109	727	14.09	60	3.040
4	6000 (UM)	0.0072	5625	7011	5976	1.68	1758	6.536
	TIC:	1.3965	ng/uL					
	TIM:	4.575	nmole/L					
	Total Conc.:	1.4383	ng/uL					

Smear Analysis 300 bp to 800 bp 1.3965 ng/ul 97.1 %Total 4.612 nmole/L 498 Avg. Size (b.p.) 7.15 %CV

Sample Peak Width (sec): 50 Sample Min Peak Height: 25 Sample Baseline V to V?: Y Sample Baseline V to V pts: 3
Sample Filter: Binomial # of Pts for Filter: 3 Sample Start Region (min): 0 Sample End Region (min): 50
Manual Baseline Start (min): 10 Manual Baseline End (min): 48
Marker Peak Width (sec): 5 Marker Min Peak Height: 200 Marker Baseline V to V?: Y Marker Baseline V to V pts: 3
Lower Marker Selection: First Peak > 200 RFU Upper Marker Selection: Last Peak > 200 RFU
Ladder Size (bp): 1, 100, 200, 300, 400, 500, 600, 700, 800, 900, 1000, 1200, 1500, 2000, 3000, 6000
Quantification Using: Ladder Final Concentration (ng/uL): 0.0830 Dilution Factor: 12.0

 Copyright 2015 Advanced Analytical Technologies, Inc.

Sample: ID13507_27
Well Location: C3
Created: Tuesday, June 27, 2017 12:18:19 PM

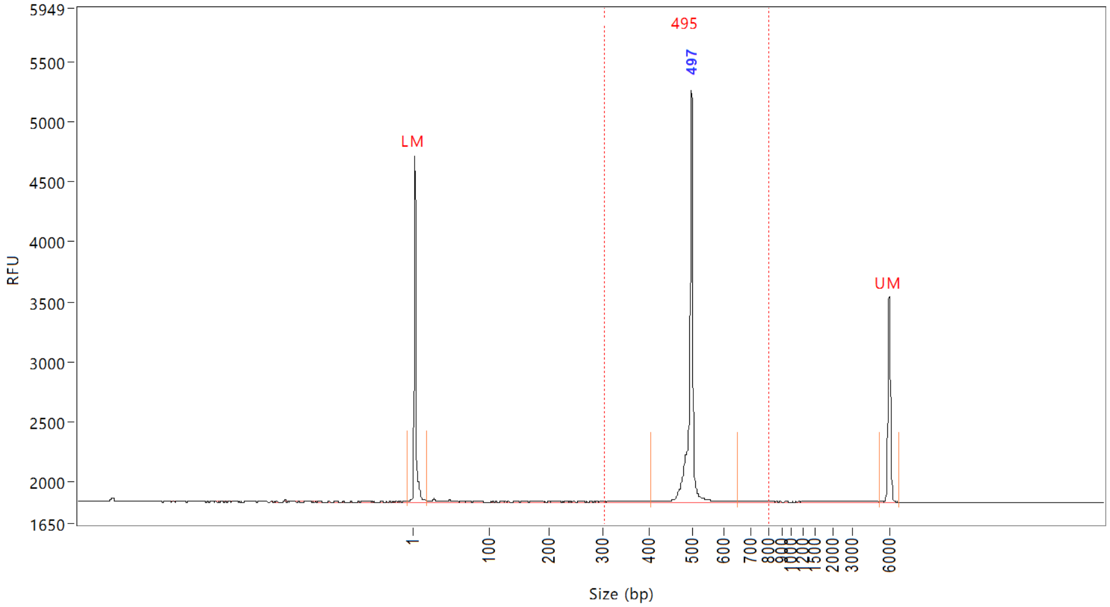

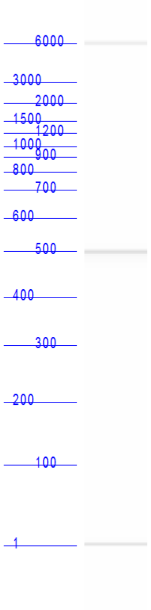

Peak	Size (bp)	Conc. (ng/uL)	From (bp)	To (bp)	Avg. Size (bp)	CV%	RFU	Corr. Peak Area
1	1 (LM)	0.0184	0	18	1	203.09	2885	16.991
2	497	0.2958	406	650	495	3.80	3434	22.800
3	6000 (UM)	0.0071	5170	6745	5966	1.99	1716	6.554

TIC:	0.2958	ng/uL
TIM:	0.979	nmole/L
Total Conc.:	0.3468	ng/uL

Smear Analysis	300 bp to 800 bp	0.3069 ng/ul	88.5 %Total	1.020 nmole/L	495 Avg. Size (b.p.)	8.00 %CV

```
Sample Peak Width (sec): 50   Sample Min Peak Height: 25    Sample Baseline V to V?: Y    Sample Baseline V to V pts: 3
Sample Filter: Binomial       # of Pts for Filter: 3        Sample Start Region (min): 0  Sample End Region (min): 50
Manual Baseline Start (min): 10     Manual Baseline End (min): 48
Marker Peak Width (sec): 5    Marker Min Peak Height: 200   Marker Baseline V to V?: Y    Marker Baseline V to V pts: 3
Lower Marker Selection: First Peak > 200 RFU                Upper Marker Selection: Last Peak > 200 RFU
Ladder Size (bp): 1, 100, 200, 300, 400, 500, 600, 700, 800, 900, 1000, 1200, 1500, 2000, 3000, 6000
Quantification Using: Ladder          Final Concentration (ng/uL): 0.0830          Dilution Factor: 12.0
```

 Copyright 2015 Advanced Analytical Technologies, Inc.

Sample: ID13507_28
Well Location: C4
Created: Tuesday, June 27, 2017 12:18:19 PM

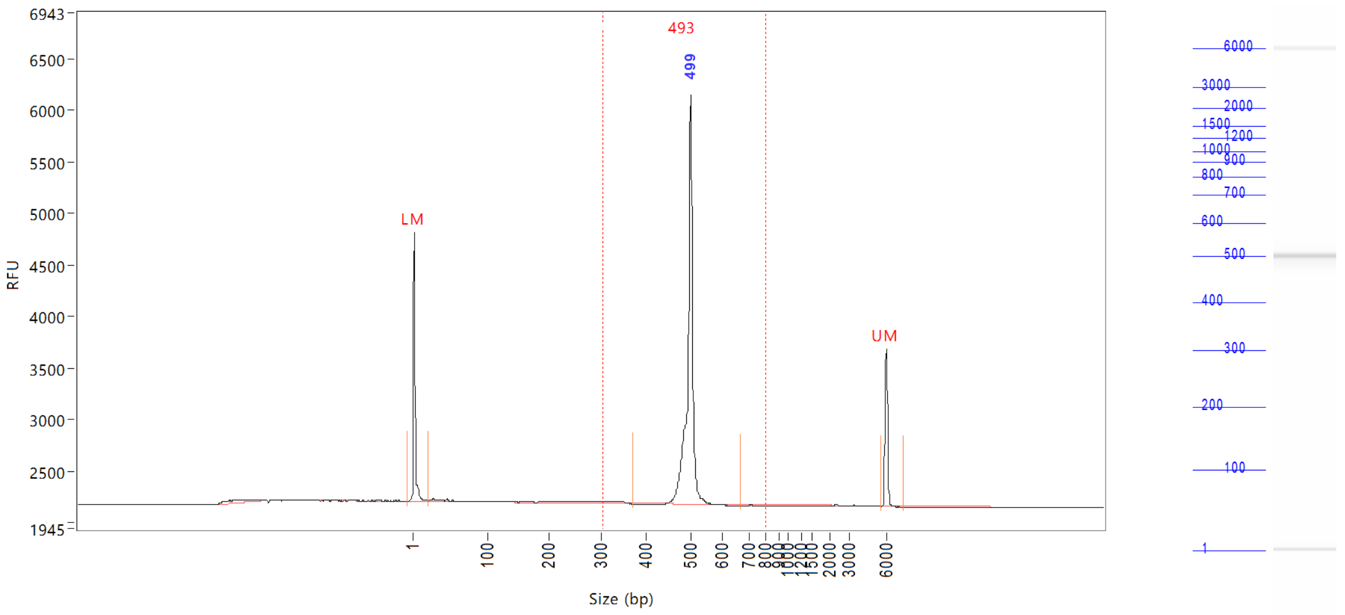

Peak	Size (bp)	Conc. (ng/uL)	From (bp)	To (bp)	Avg. Size (bp)	CV%	RFU	Corr. Peak Area
1	1 (LM)	0.0184	0	20	1	220.73	2604	15.754
2	499	0.5042	369	672	495	2.42	3961	36.039
3	6000 (UM)	0.0065	5545	7330	5966	1.55	1513	5.611

TIC:	0.5042	ng/uL
TIM:	1.662	nmole/L
Total Conc.:	0.5556	ng/uL

Smear Analysis 300 bp to 800 bp 0.5124 ng/ul 92.2 %Total 1.711 nmole/L 493 Avg. Size (b.p.) 4.92 %CV

```
Sample Peak Width (sec): 50   Sample Min Peak Height: 25    Sample Baseline V to V?: Y    Sample Baseline V to V pts: 3
Sample Filter: Binomial       # of Pts for Filter: 3        Sample Start Region (min): 0  Sample End Region (min): 50
Manual Baseline Start (min): 10     Manual Baseline End (min): 48
Marker Peak Width (sec): 5    Marker Min Peak Height: 200   Marker Baseline V to V?: Y    Marker Baseline V to V pts: 3
Lower Marker Selection: First Peak > 200 RFU                Upper Marker Selection: Last Peak > 200 RFU
Ladder Size (bp): 1, 100, 200, 300, 400, 500, 600, 700, 800, 900, 1000, 1200, 1500, 2000, 3000, 6000
Quantification Using: Ladder          Final Concentration (ng/uL): 0.0830          Dilution Factor: 12.0
```

 Copyright 2015 Advanced Analytical Technologies, Inc.

Sample: ID13507_31
Well Location: C7
Created: Tuesday, June 27, 2017 12:18:19 PM

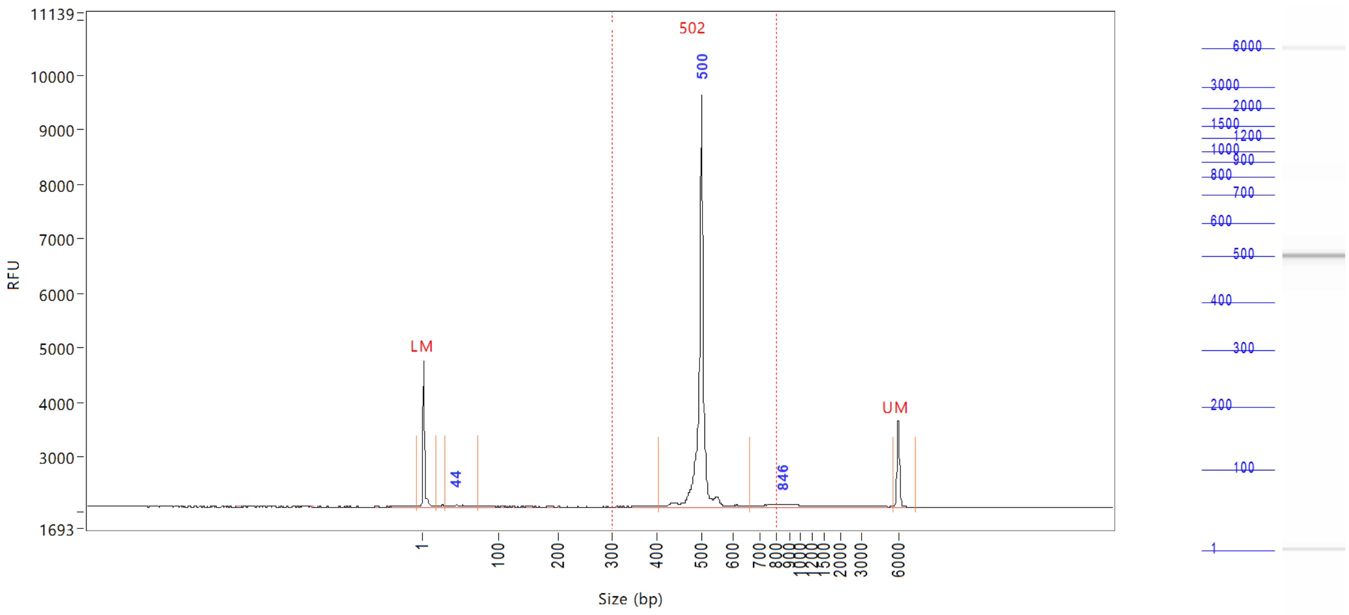

Peak	Size (bp)	Conc. (ng/uL)	From (bp)	To (bp)	Avg. Size (bp)	CV%	RFU	Corr. Peak Area
1	1 (LM)	0.0184	0	18	1	191.69	2660	15.790
2	44	0.0156	31	72	49	20.87	41	1.121
3	500	0.8893	403	662	499	4.69	7543	63.710
4	846	0.0747	662	5598	1554	74.33	59	5.352
5	6000 (UM)	0.0072	5598	7436	6010	2.61	1580	6.154

TIC:	0.9797	ng/uL
TIM:	3.648	nmole/L
Total Conc.:	1.0120	ng/uL

Smear Analysis 300 bp to 800 bp 0.9204 ng/ul 90.9 %Total 3.016 nmole/L 502 Avg. Size (b.p.) 8.86 %CV

```
Sample Peak Width (sec): 50   Sample Min Peak Height: 25    Sample Baseline V to V?: Y    Sample Baseline V to V pts: 3
Sample Filter: Binomial       # of Pts for Filter: 3        Sample Start Region (min): 0  Sample End Region (min): 50
Manual Baseline Start (min): 10     Manual Baseline End (min): 48
Marker Peak Width (sec): 5    Marker Min Peak Height: 200   Marker Baseline V to V?: Y    Marker Baseline V to V pts: 3
Lower Marker Selection: First Peak > 200 RFU                Upper Marker Selection: Last Peak > 200 RFU
Ladder Size (bp): 1, 100, 200, 300, 400, 500, 600, 700, 800, 900, 1000, 1200, 1500, 2000, 3000, 6000
Quantification Using: Ladder          Final Concentration (ng/uL): 0.0830          Dilution Factor: 12.0
```

 Copyright 2015 Advanced Analytical Technologies, Inc.

Sample: ID13507_33
Well Location: C9
Created: Tuesday, June 27, 2017 12:18:19 PM

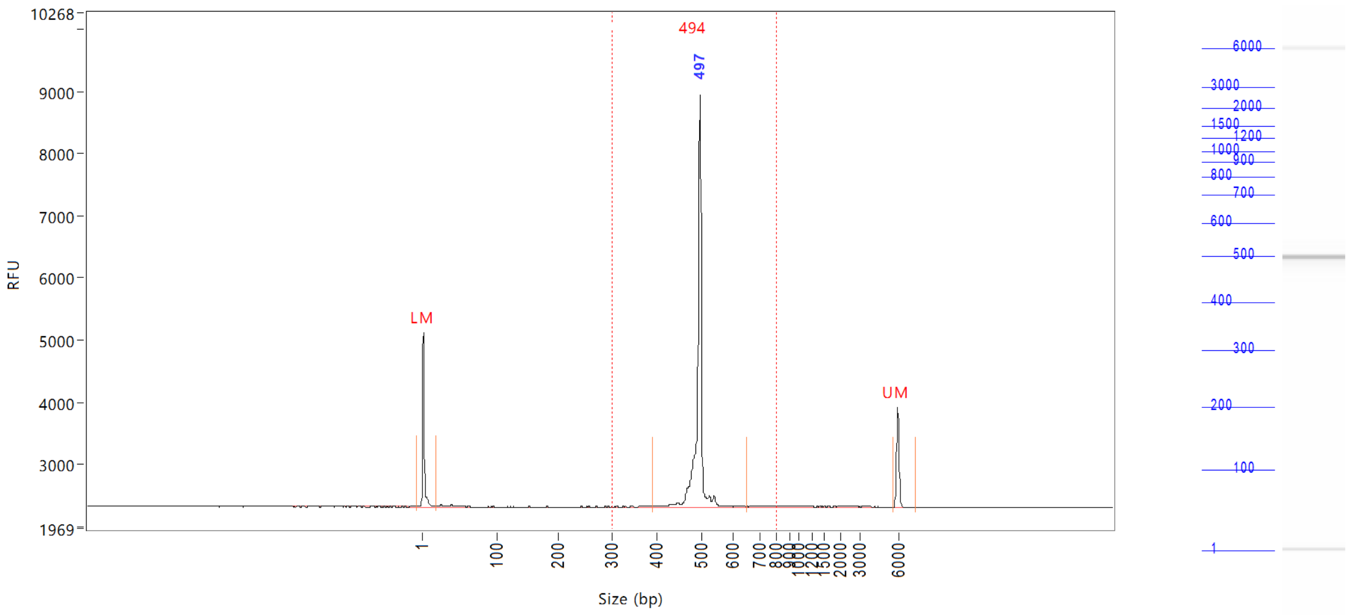

Peak	Size (bp)	Conc. (ng/uL)	From (bp)	To (bp)	Avg. Size (bp)	CV%	RFU	Corr. Peak Area
1	1 (LM)	0.0184	0	18	1	191.63	2800	16.583
2	497	0.6646	391	652	493	3.66	6626	50.006
3	6000 (UM)	0.0066	5625	7463	5981	1.63	1604	5.998

TIC:	0.6646	ng/uL
TIM:	2.201	nmole/L
Total Conc.:	0.7064	ng/uL

Smear Analysis	300 bp to 800 bp	0.6750 ng/ul	95.5 %Total	2.247 nmole/L	494 Avg. Size (b.p.)	6.23 %CV

```
Sample Peak Width (sec): 50   Sample Min Peak Height: 25    Sample Baseline V to V?: Y    Sample Baseline V to V pts: 3
Sample Filter: Binomial       # of Pts for Filter: 3        Sample Start Region (min): 0  Sample End Region (min): 50
Manual Baseline Start (min): 10     Manual Baseline End (min): 48
Marker Peak Width (sec): 5    Marker Min Peak Height: 200   Marker Baseline V to V?: Y    Marker Baseline V to V pts: 3
Lower Marker Selection: First Peak > 200 RFU                Upper Marker Selection: Last Peak > 200 RFU
Ladder Size (bp): 1, 100, 200, 300, 400, 500, 600, 700, 800, 900, 1000, 1200, 1500, 2000, 3000, 6000
Quantification Using: Ladder          Final Concentration (ng/uL): 0.0830          Dilution Factor: 12.0
```

 Copyright 2015 Advanced Analytical Technologies, Inc.

Sample: ID13507_34
Well Location: C10
Created: Tuesday, June 27, 2017 12:18:19 PM

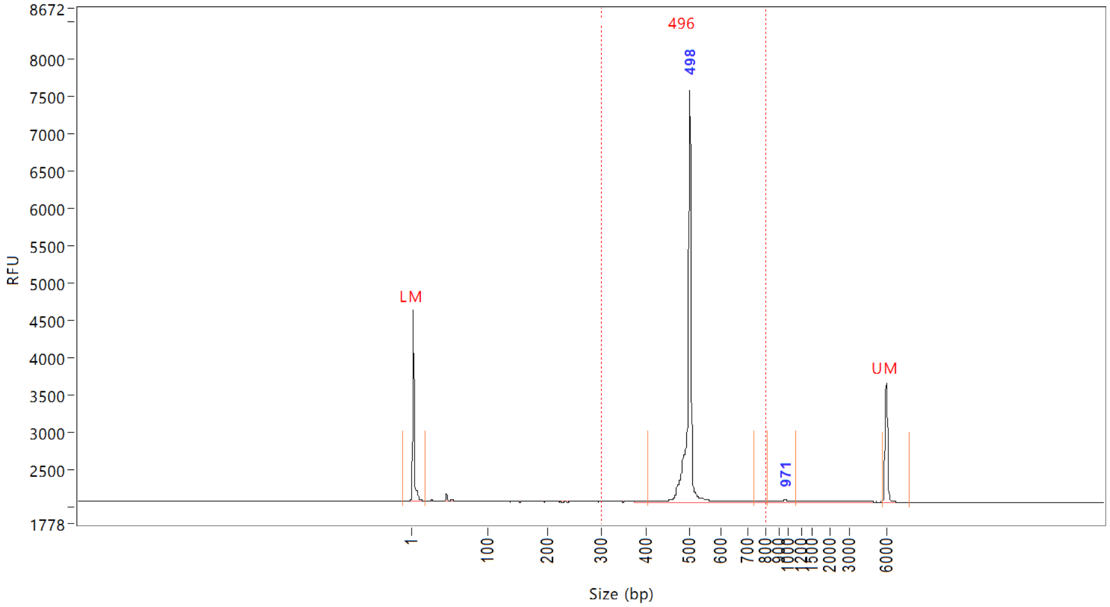

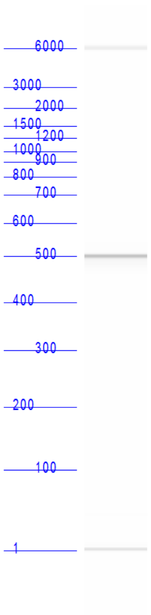

Peak	Size (bp)	Conc. (ng/uL)	From (bp)	To (bp)	Avg. Size (bp)	CV%	RFU	Corr. Peak Area
1	1 (LM)	0.0184	0	18	1	198.80	2557	15.259
2	498	0.5158	403	733	496	4.14	5502	35.711
3	971	0.0057	818	1125	947	7.42	33	0.393
4	6000 (UM)	0.0073	5679	7862	5992	1.83	1594	6.054

TIC:	0.5215	ng/uL
TIM:	1.712	nmole/L
Total Conc.:	0.5668	ng/uL

Smear Analysis 300 bp to 800 bp 0.5210 ng/ul 91.9 %Total 1.728 nmole/L 496 Avg. Size (b.p.) 5.68 %CV

```
Sample Peak Width (sec): 50   Sample Min Peak Height: 25    Sample Baseline V to V?: Y    Sample Baseline V to V pts: 3
Sample Filter: Binomial       # of Pts for Filter: 3        Sample Start Region (min): 0  Sample End Region (min): 50
Manual Baseline Start (min): 10     Manual Baseline End (min): 48
Marker Peak Width (sec): 5    Marker Min Peak Height: 200   Marker Baseline V to V?: Y    Marker Baseline V to V pts: 3
Lower Marker Selection: First Peak > 200 RFU                Upper Marker Selection: Last Peak > 200 RFU
Ladder Size (bp): 1, 100, 200, 300, 400, 500, 600, 700, 800, 900, 1000, 1200, 1500, 2000, 3000, 6000
Quantification Using: Ladder          Final Concentration (ng/uL): 0.0830          Dilution Factor: 12.0
```

 Copyright 2015 Advanced Analytical Technologies, Inc.

Sample: ID13507_36
Well Location: C12
Created: Tuesday, June 27, 2017 12:18:19 PM

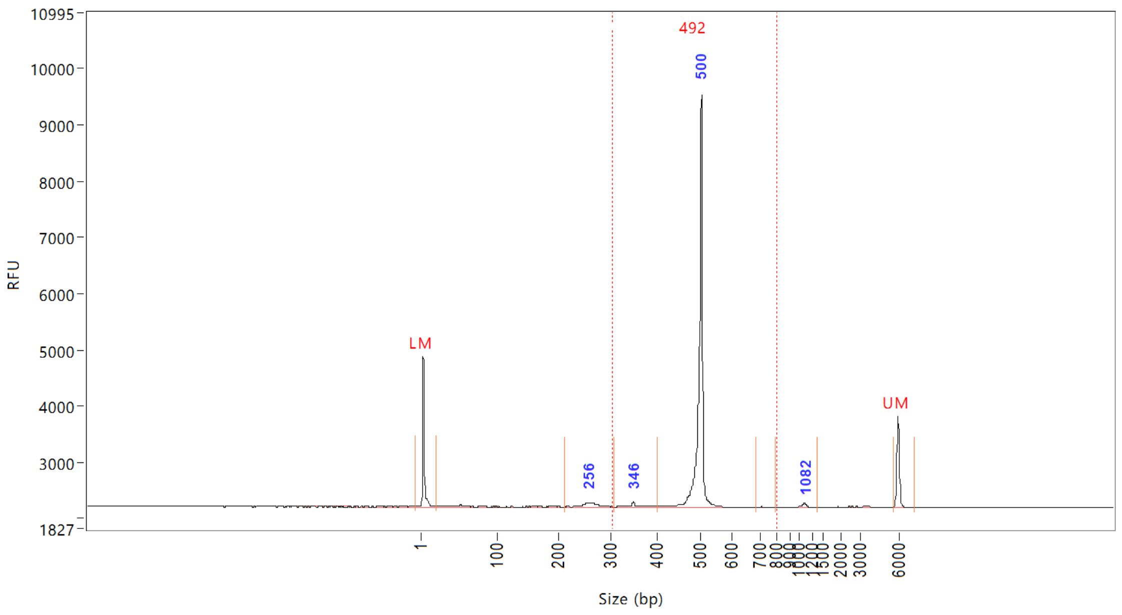

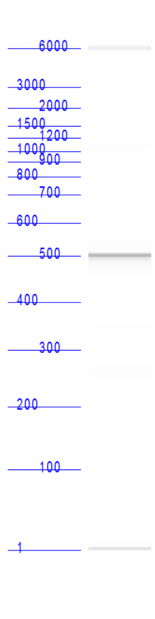

Peak	Size (bp)	Conc. (ng/uL)	From (bp)	To (bp)	Avg. Size (bp)	CV%	RFU	Corr. Peak Area
1	1 (LM)	0.0184	0	20	2	159.99	2667	15.976
2	256	0.0362	212	306	259	5.89	83	2.622
3	346	0.0191	306	400	353	6.14	99	1.381
4	500	0.6680	400	685	496	3.04	7323	48.418
5	1082	0.0098	790	1346	1039	9.36	81	0.713
6	6000 (UM)	0.0071	5625	7303	5982	2.12	1627	6.170

TIC:	0.7330	ng/uL
TIM:	2.536	nmole/L
Total Conc.:	0.7670	ng/uL

Smear Analysis	300 bp to 800 bp	0.6886 ng/ul	89.8 %Total	2.303 nmole/L	492 Avg. Size (b.p.)	6.15 %CV

Sample Peak Width (sec): 50 Sample Min Peak Height: 25 Sample Baseline V to V?: Y Sample Baseline V to V pts: 3
Sample Filter: Binomial # of Pts for Filter: 3 Sample Start Region (min): 0 Sample End Region (min): 50
Manual Baseline Start (min): 10 Manual Baseline End (min): 48
Marker Peak Width (sec): 5 Marker Min Peak Height: 200 Marker Baseline V to V?: Y Marker Baseline V to V pts: 3
Lower Marker Selection: First Peak > 200 RFU Upper Marker Selection: Last Peak > 200 RFU
Ladder Size (bp): 1, 100, 200, 300, 400, 500, 600, 700, 800, 900, 1000, 1200, 1500, 2000, 3000, 6000
Quantification Using: Ladder Final Concentration (ng/uL): 0.0830 Dilution Factor: 12.0

 Copyright 2015 Advanced Analytical Technologies, Inc.

Sample: ID13507_40
Well Location: D4
Created: Tuesday, June 27, 2017 12:18:19 PM

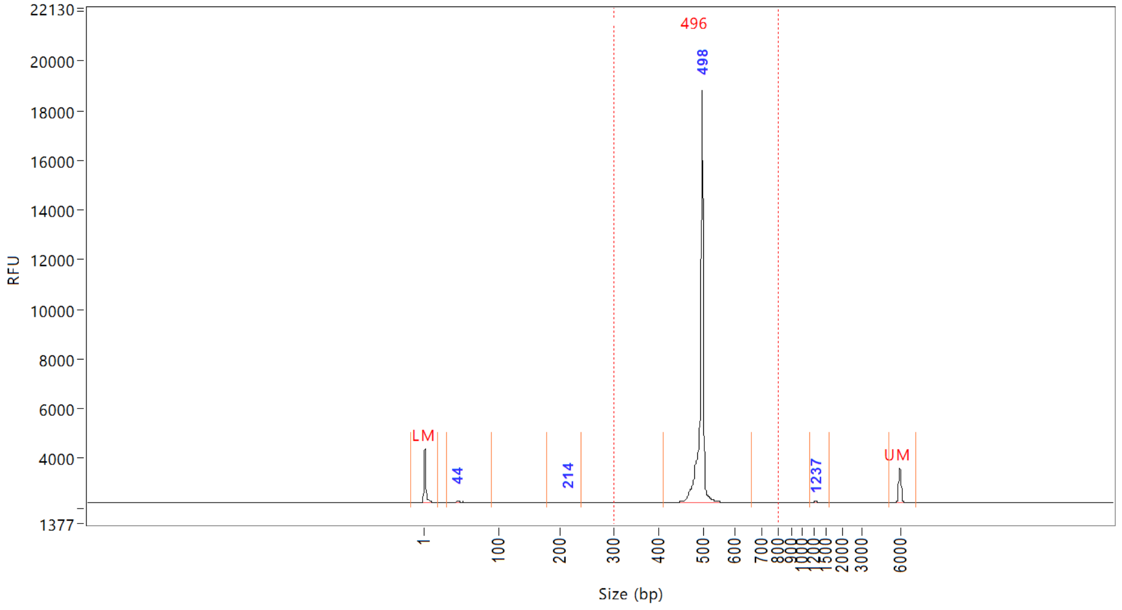

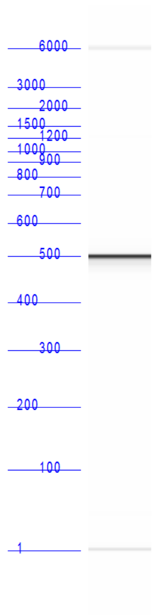

Peak	Size (bp)	Conc. (ng/uL)	From (bp)	To (bp)	Avg. Size (bp)	CV%	RFU	Corr. Peak Area
1	1 (LM)	0.0184	0	19	1	236.02	2195	13.301
2	44	0.0223	30	89	48	21.84	75	1.348
3	214	0.0063	179	240	210	7.43	27	0.380
4	498	1.6929	409	664	495	3.24	16594	102.161
5	1237	0.0134	1135	1628	1340	10.72	56	0.809
6	6000 (UM)	0.0074	5090	7330	5984	2.66	1363	5.333

TIC:	1.7349	ng/uL
TIM:	6.493	nmole/L
Total Conc.:	1.8231	ng/uL

Smear Analysis 300 bp to 800 bp 1.7257 ng/ul 94.7 %Total 5.721 nmole/L 496 Avg. Size (b.p.) 6.43 %CV

```
Sample Peak Width (sec): 50   Sample Min Peak Height: 25    Sample Baseline V to V?: Y    Sample Baseline V to V pts: 3
Sample Filter: Binomial       # of Pts for Filter: 3        Sample Start Region (min): 0  Sample End Region (min): 50
Manual Baseline Start (min): 10     Manual Baseline End (min): 48
Marker Peak Width (sec): 5    Marker Min Peak Height: 200   Marker Baseline V to V?: Y    Marker Baseline V to V pts: 3
Lower Marker Selection: First Peak > 200 RFU                Upper Marker Selection: Last Peak > 200 RFU
Ladder Size (bp): 1, 100, 200, 300, 400, 500, 600, 700, 800, 900, 1000, 1200, 1500, 2000, 3000, 6000
Quantification Using: Ladder          Final Concentration (ng/uL): 0.0830          Dilution Factor: 12.0
```

 Copyright 2015 Advanced Analytical Technologies, Inc.

Sample: ID13507_41
Well Location: D5
Created: Tuesday, June 27, 2017 12:18:19 PM

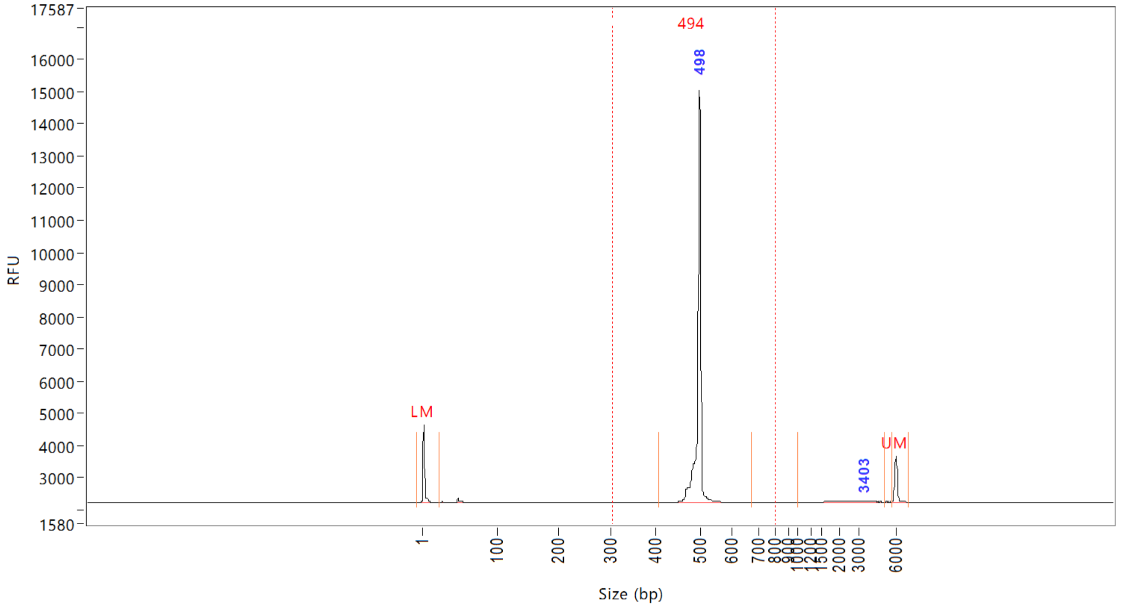

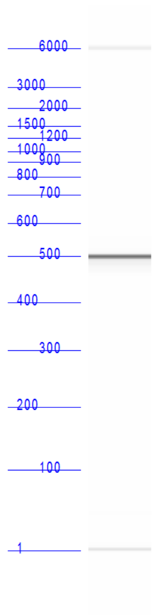

Peak	Size (bp)	Conc. (ng/uL)	From (bp)	To (bp)	Avg. Size (bp)	CV%	RFU	Corr. Peak Area
1	1 (LM)	0.0184	0	23	1	216.98	2389	14.192
2	498	1.1419	404	674	494	2.22	12791	73.527
3	3403	0.0284	1001	5063	2792	34.26	29	1.829
4	6000 (UM)	0.0072	5652	7037	5999	2.53	1422	5.564

TIC:	1.1704	ng/uL
TIM:	3.789	nmole/L
Total Conc.:	1.1935	ng/uL

Smear Analysis	300 bp to 800 bp	1.1419 ng/ul	95.7 %Total	3.802 nmole/L	494 Avg. Size (b.p.)	2.22 %CV

```
Sample Peak Width (sec): 50   Sample Min Peak Height: 25    Sample Baseline V to V?: Y    Sample Baseline V to V pts: 3
Sample Filter: Binomial       # of Pts for Filter: 3        Sample Start Region (min): 0  Sample End Region (min): 50
Manual Baseline Start (min): 10     Manual Baseline End (min): 48
Marker Peak Width (sec): 5    Marker Min Peak Height: 200   Marker Baseline V to V?: Y    Marker Baseline V to V pts: 3
Lower Marker Selection: First Peak > 200 RFU                Upper Marker Selection: Last Peak > 200 RFU
Ladder Size (bp): 1, 100, 200, 300, 400, 500, 600, 700, 800, 900, 1000, 1200, 1500, 2000, 3000, 6000
Quantification Using: Ladder          Final Concentration (ng/uL): 0.0830          Dilution Factor: 12.0
```

 Copyright 2015 Advanced Analytical Technologies, Inc.

Sample: ID13507_45
Well Location: D9
Created: Tuesday, June 27, 2017 12:18:19 PM

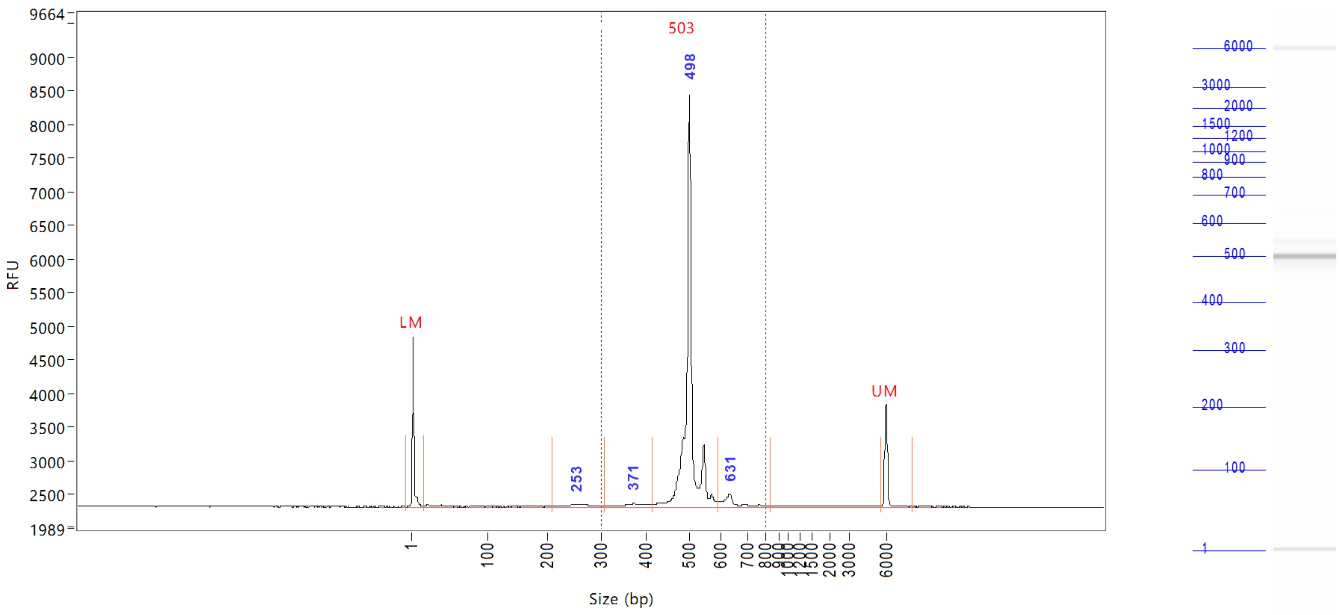

Peak	Size (bp)	Conc. (ng/uL)	From (bp)	To (bp)	Avg. Size (bp)	CV%	RFU	Corr. Peak Area
1	1 (LM)	0.0184	0	17	1	181.68	2528	15.202
2	253	0.0301	208	308	261	8.09	43	2.075
3	371	0.0384	308	414	370	7.21	50	2.650
4	498	0.9506	414	589	500	4.87	6130	65.566
5	631	0.0615	589	836	653	9.13	196	4.241
6	6000 (UM)	0.0076	5652	8101	6035	4.99	1540	6.250

TIC:	1.0806	ng/uL
TIM:	3.665	nmole/L
Total Conc.:	1.1389	ng/uL

Smear Analysis	300 bp to 800 bp	1.0485 ng/ul	92.1 %Total	3.431 nmole/L	503 Avg. Size (b.p.)	9.97 %CV

Sample Peak Width (sec): 50 Sample Min Peak Height: 25 Sample Baseline V to V?: Y Sample Baseline V to V pts: 3
Sample Filter: Binomial # of Pts for Filter: 3 Sample Start Region (min): 0 Sample End Region (min): 50
Manual Baseline Start (min): 10 Manual Baseline End (min): 48
Marker Peak Width (sec): 5 Marker Min Peak Height: 200 Marker Baseline V to V?: Y Marker Baseline V to V pts: 3
Lower Marker Selection: First Peak > 200 RFU Upper Marker Selection: Last Peak > 200 RFU
Ladder Size (bp): 1, 100, 200, 300, 400, 500, 600, 700, 800, 900, 1000, 1200, 1500, 2000, 3000, 6000
Quantification Using: Ladder Final Concentration (ng/uL): 0.0830 Dilution Factor: 12.0

 Copyright 2015 Advanced Analytical Technologies, Inc.

Sample: ID13507_47
Well Location: D11
Created: Tuesday, June 27, 2017 12:18:19 PM

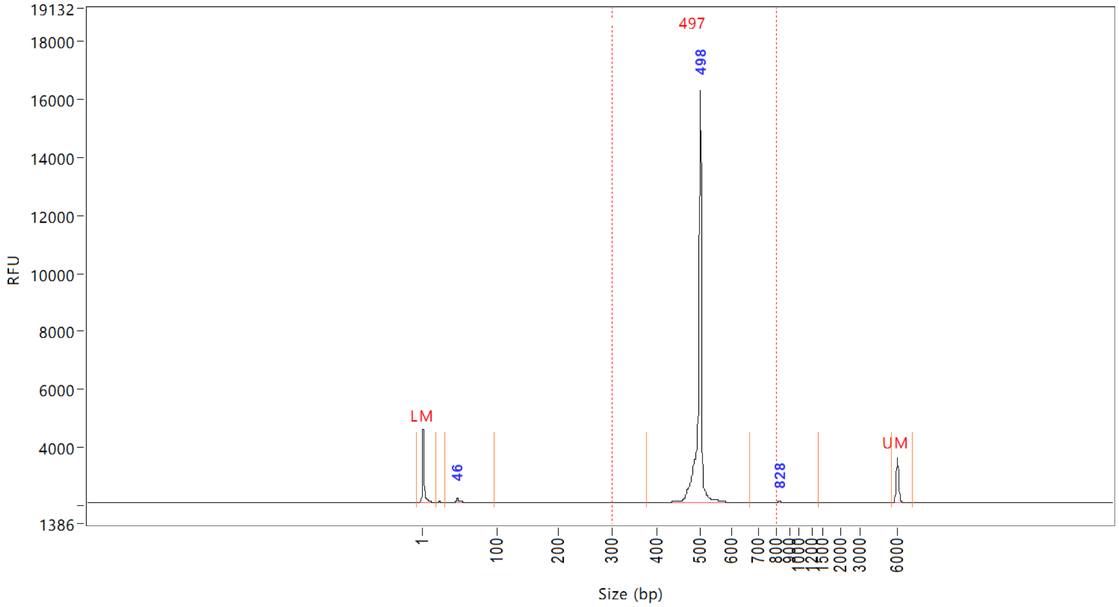

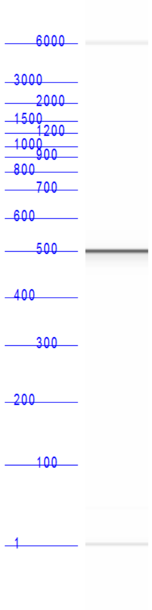

Peak	Size (bp)	Conc. (ng/uL)	From (bp)	To (bp)	Avg. Size (bp)	CV%	RFU	Corr. Peak Area
1	1 (LM)	0.0184	0	18	1	221.54	2519	15.421
2	46	0.0247	30	95	49	20.71	164	1.726
3	498	1.2671	378	667	495	3.41	14186	88.654
4	828	0.0208	667	1391	839	18.95	35	1.459
5	6000 (UM)	0.0069	5598	7250	5988	1.76	1538	5.815

TIC:	1.3126	ng/uL
TIM:	5.098	nmole/L
Total Conc.:	1.3297	ng/uL

Smear Analysis 300 bp to 800 bp 1.2792 ng/ul 96.2 %Total 4.235 nmole/L 497 Avg. Size (b.p.) 5.65 %CV

```
Sample Peak Width (sec): 50   Sample Min Peak Height: 25    Sample Baseline V to V?: Y    Sample Baseline V to V pts: 3
Sample Filter: Binomial       # of Pts for Filter: 3        Sample Start Region (min): 0  Sample End Region (min): 50
Manual Baseline Start (min): 10     Manual Baseline End (min): 48
Marker Peak Width (sec): 5    Marker Min Peak Height: 200   Marker Baseline V to V?: Y    Marker Baseline V to V pts: 3
Lower Marker Selection: First Peak > 200 RFU                Upper Marker Selection: Last Peak > 200 RFU
Ladder Size (bp): 1, 100, 200, 300, 400, 500, 600, 700, 800, 900, 1000, 1200, 1500, 2000, 3000, 6000
Quantification Using: Ladder          Final Concentration (ng/uL): 0.0830          Dilution Factor: 12.0
```

 Copyright 2015 Advanced Analytical Technologies, Inc.

Sample: ID13507_53
Well Location: E5
Created: Tuesday, June 27, 2017 12:18:19 PM

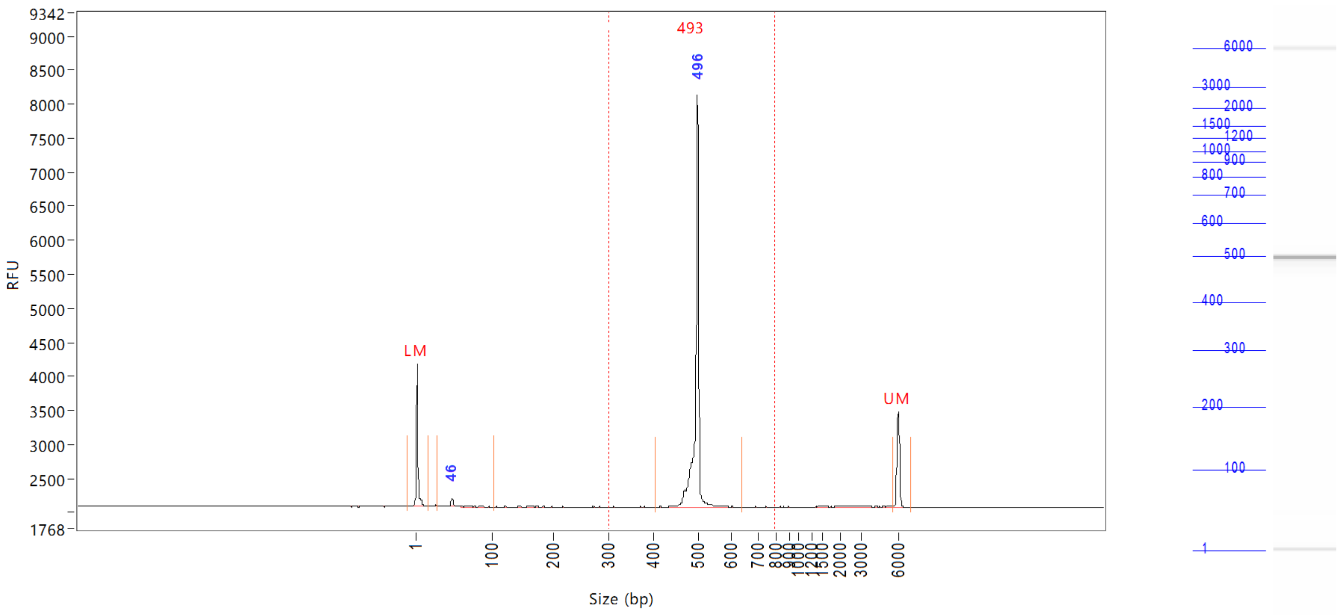

Peak	Size (bp)	Conc. (ng/uL)	From (bp)	To (bp)	Avg. Size (bp)	CV%	RFU	Corr. Peak Area
1	1 (LM)	0.0184	0	17	1	203.34	2105	12.410
2	46	0.0239	28	102	47	19.76	120	1.345
3	496	0.6582	403	643	493	2.77	6049	37.061
4	6000 (UM)	0.0079	5625	7011	5966	1.82	1413	5.338

TIC:	0.6821	ng/uL
TIM:	3.028	nmole/L
Total Conc.:	0.7208	ng/uL

Smear Analysis 300 bp to 800 bp 0.6633 ng/ul 92.0 %Total 2.213 nmole/L 493 Avg. Size (b.p.) 4.38 %CV

```
Sample Peak Width (sec): 50   Sample Min Peak Height: 25    Sample Baseline V to V?: Y    Sample Baseline V to V pts: 3
Sample Filter: Binomial       # of Pts for Filter: 3        Sample Start Region (min): 0  Sample End Region (min): 50
Manual Baseline Start (min): 10     Manual Baseline End (min): 48
Marker Peak Width (sec): 5    Marker Min Peak Height: 200   Marker Baseline V to V?: Y    Marker Baseline V to V pts: 3
Lower Marker Selection: First Peak > 200 RFU                Upper Marker Selection: Last Peak > 200 RFU
Ladder Size (bp): 1, 100, 200, 300, 400, 500, 600, 700, 800, 900, 1000, 1200, 1500, 2000, 3000, 6000
Quantification Using: Ladder          Final Concentration (ng/uL): 0.0830          Dilution Factor: 12.0
```

Sample: ID13507_55
Well Location: E7
Created: Tuesday, June 27, 2017 12:18:19 PM

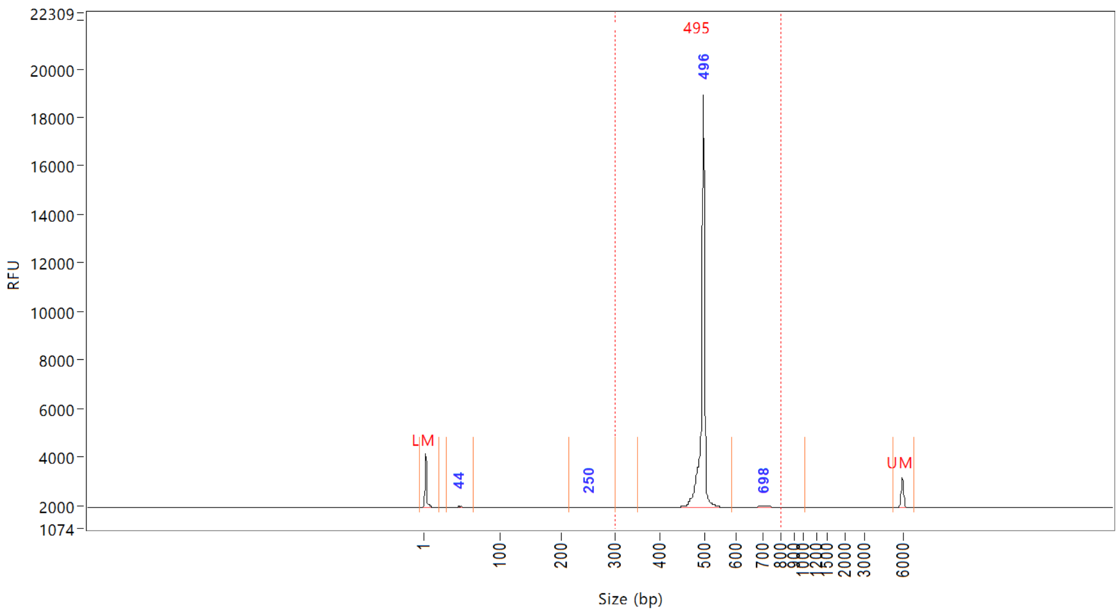

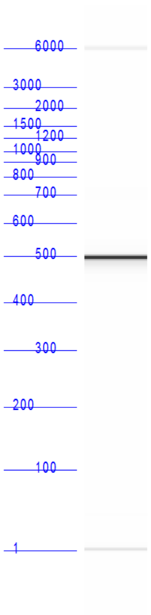

Peak	Size (bp)	Conc. (ng/uL)	From (bp)	To (bp)	Avg. Size (bp)	CV%	RFU	Corr. Peak Area
1	1 (LM)	0.0184	0	19	1	204.78	2192	12.994
2	44	0.0145	29	65	46	17.07	49	0.858
3	250	0.0170	215	299	259	5.86	28	1.003
4	496	1.7392	349	588	492	2.98	16980	102.535
5	698	0.0396	588	1033	699	9.49	61	2.336
6	6000 (UM)	0.0066	5250	6957	5967	2.11	1217	4.684

TIC:	1.8104	ng/uL
TIM:	6.508	nmole/L
Total Conc.:	1.8454	ng/uL

Smear Analysis	300 bp to 800 bp	1.7797 ng/ul	96.4 %Total	5.911 nmole/L	495 Avg. Size (b.p.)	6.70 %CV

```
Sample Peak Width (sec): 50   Sample Min Peak Height: 25    Sample Baseline V to V?: Y    Sample Baseline V to V pts: 3
Sample Filter: Binomial       # of Pts for Filter: 3        Sample Start Region (min): 0  Sample End Region (min): 50
Manual Baseline Start (min): 10     Manual Baseline End (min): 48
Marker Peak Width (sec): 5    Marker Min Peak Height: 200   Marker Baseline V to V?: Y    Marker Baseline V to V pts: 3
Lower Marker Selection: First Peak > 200 RFU                Upper Marker Selection: Last Peak > 200 RFU
Ladder Size (bp): 1, 100, 200, 300, 400, 500, 600, 700, 800, 900, 1000, 1200, 1500, 2000, 3000, 6000
Quantification Using: Ladder          Final Concentration (ng/uL): 0.0830         Dilution Factor: 12.0
```

 Copyright 2015 Advanced Analytical Technologies, Inc.

Sample: ID13507_57
Well Location: E9
Created: Tuesday, June 27, 2017 12:18:19 PM

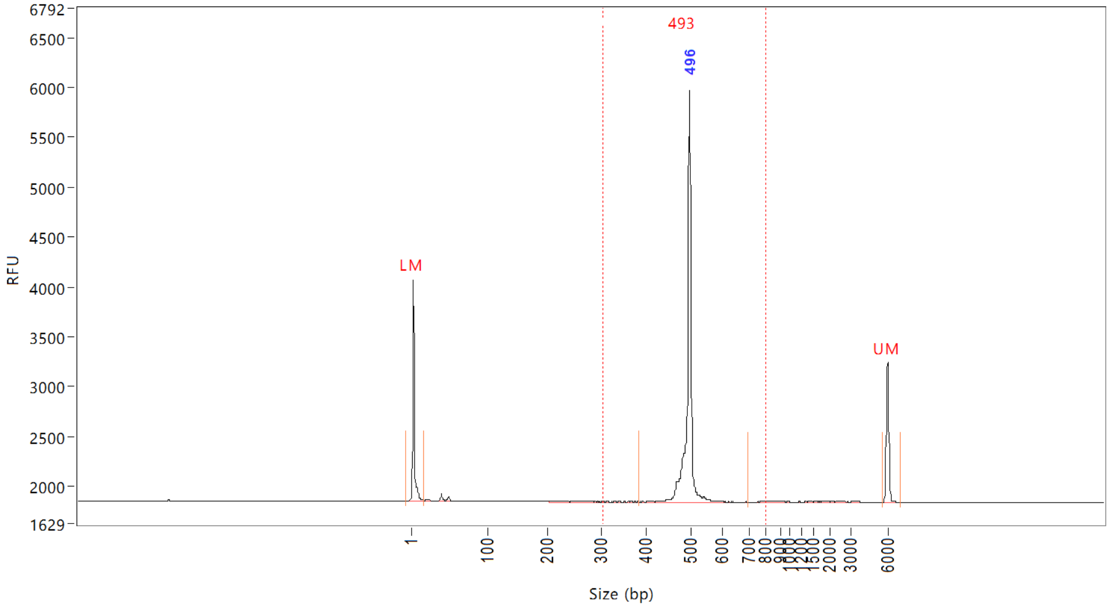

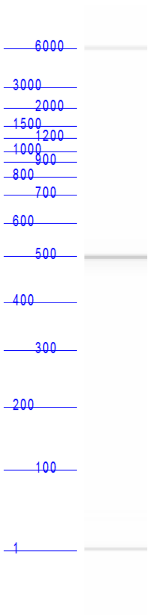

Peak	Size (bp)	Conc. (ng/uL)	From (bp)	To (bp)	Avg. Size (bp)	CV%	RFU	Corr. Peak Area
1	1 (LM)	0.0184	0	16	2	166.84	2218	13.640
2	496	0.4847	383	698	493	3.42	4122	29.998
3	6000 (UM)	0.0071	5652	7037	5983	1.76	1408	5.291

TIC:	0.4847	ng/uL
TIM:	1.607	nmole/L
Total Conc.:	0.5421	ng/uL

Smear Analysis 300 bp to 800 bp 0.4875 ng/ul 89.9 %Total 1.626 nmole/L 493 Avg. Size (b.p.) 4.78 %CV

```
Sample Peak Width (sec): 50   Sample Min Peak Height: 25    Sample Baseline V to V?: Y    Sample Baseline V to V pts: 3
Sample Filter: Binomial       # of Pts for Filter: 3        Sample Start Region (min): 0  Sample End Region (min): 50
Manual Baseline Start (min): 10     Manual Baseline End (min): 48
Marker Peak Width (sec): 5    Marker Min Peak Height: 200   Marker Baseline V to V?: Y    Marker Baseline V to V pts: 3
Lower Marker Selection: First Peak > 200 RFU                Upper Marker Selection: Last Peak > 200 RFU
Ladder Size (bp): 1, 100, 200, 300, 400, 500, 600, 700, 800, 900, 1000, 1200, 1500, 2000, 3000, 6000
Quantification Using: Ladder          Final Concentration (ng/uL): 0.0830          Dilution Factor: 12.0
```

 Copyright 2015 Advanced Analytical Technologies, Inc.

Sample: ID13507_70
Well Location: F10
Created: Tuesday, June 27, 2017 12:18:19 PM

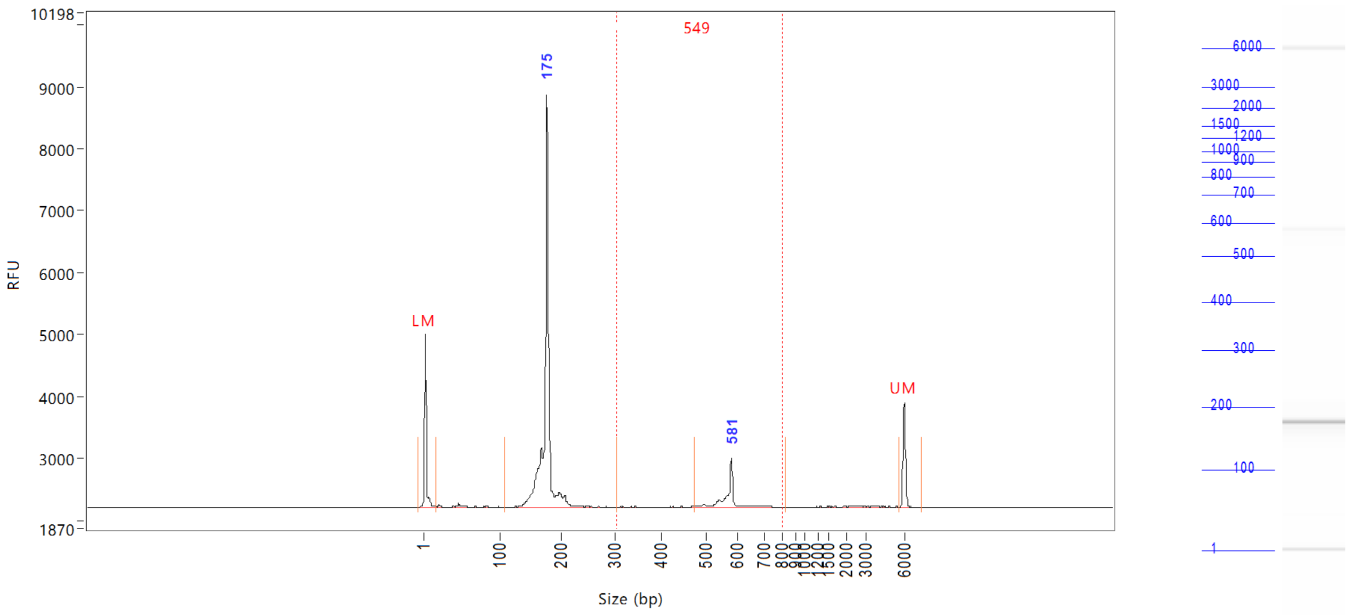

Peak	Size (bp)	Conc. (ng/uL)	From (bp)	To (bp)	Avg. Size (bp)	CV%	RFU	Corr. Peak Area
1	1 (LM)	0.0184	0	17	1	219.04	2793	16.953
2	175	0.9179	106	303	173	9.00	6657	70.601
3	581	0.1074	474	828	576	8.89	806	8.263
4	6000 (UM)	0.0070	5652	7383	5996	2.22	1691	6.483

TIC:	1.0253	ng/uL
TIM:	8.941	nmole/L
Total Conc.:	1.0886	ng/uL

Smear Analysis 300 bp to 800 bp 0.1232 ng/ul 11.3 %Total 0.369 nmole/L 549 Avg. Size (b.p.) 14.68 %CV

```
Sample Peak Width (sec): 50   Sample Min Peak Height: 25    Sample Baseline V to V?: Y    Sample Baseline V to V pts: 3
Sample Filter: Binomial       # of Pts for Filter: 3        Sample Start Region (min): 0  Sample End Region (min): 50
Manual Baseline Start (min): 10     Manual Baseline End (min): 48
Marker Peak Width (sec): 5    Marker Min Peak Height: 200   Marker Baseline V to V?: Y    Marker Baseline V to V pts: 3
Lower Marker Selection: First Peak > 200 RFU                Upper Marker Selection: Last Peak > 200 RFU
Ladder Size (bp): 1, 100, 200, 300, 400, 500, 600, 700, 800, 900, 1000, 1200, 1500, 2000, 3000, 6000
Quantification Using: Ladder          Final Concentration (ng/uL): 0.0830          Dilution Factor: 12.0
```


Copyright 2015 Advanced Analytical Technologies, Inc.

Sample: ID13507_71
Well Location: F11
Created: Tuesday, June 27, 2017 12:18:19 PM

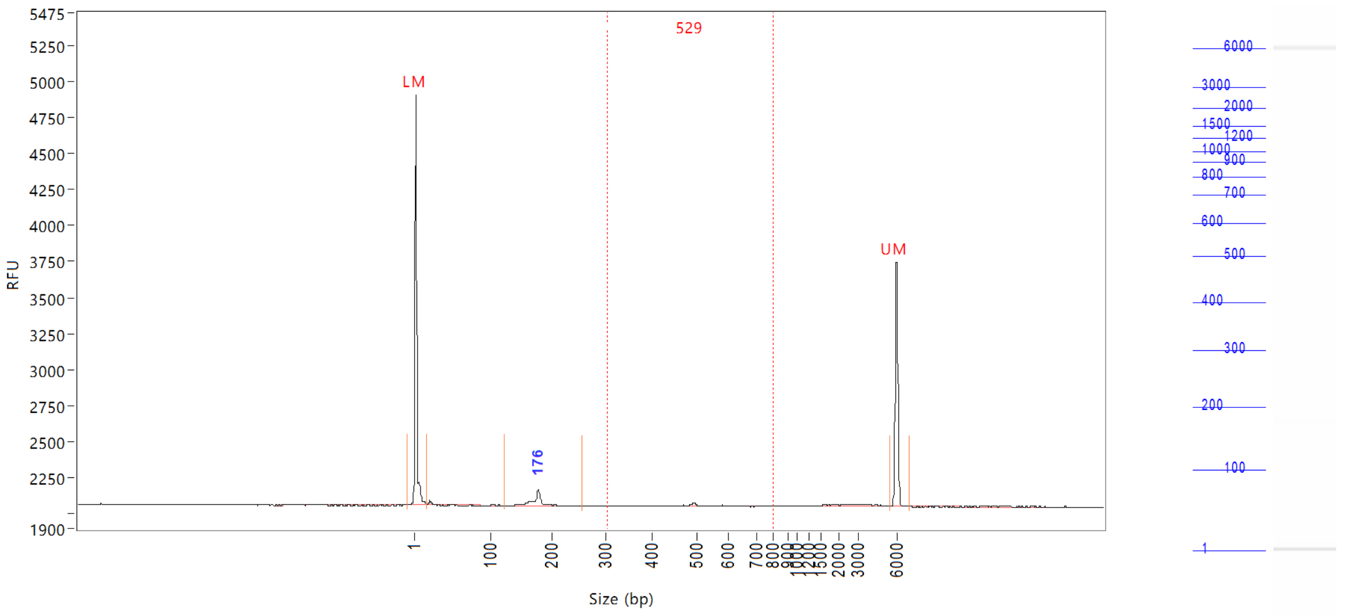

Peak	Size (bp)	Conc. (ng/uL)	From (bp)	To (bp)	Avg. Size (bp)	CV%	RFU	Corr. Peak Area
1	1 (LM)	0.0184	0	16	1	181.09	2841	16.800
2	176	0.0224	122	254	171	6.54	114	1.705
3	6000 (UM)	0.0071	5491	7064	5989	1.71	1691	6.466

TIC:	0.0224	ng/uL
TIM:	0.209	nmole/L
Total Conc.:	0.0443	ng/uL

Smear Analysis 300 bp to 800 bp 0.0034 ng/ul 7.6 %Total 0.010 nmole/L 529 Avg. Size (b.p.) 19.51 %CV

```
Sample Peak Width (sec): 50   Sample Min Peak Height: 25    Sample Baseline V to V?: Y    Sample Baseline V to V pts: 3
Sample Filter: Binomial       # of Pts for Filter: 3        Sample Start Region (min): 0  Sample End Region (min): 50
Manual Baseline Start (min): 10     Manual Baseline End (min): 48
Marker Peak Width (sec): 5    Marker Min Peak Height: 200   Marker Baseline V to V?: Y    Marker Baseline V to V pts: 3
Lower Marker Selection: First Peak > 200 RFU                Upper Marker Selection: Last Peak > 200 RFU
Ladder Size (bp): 1, 100, 200, 300, 400, 500, 600, 700, 800, 900, 1000, 1200, 1500, 2000, 3000, 6000
Quantification Using: Ladder          Final Concentration (ng/uL): 0.0830          Dilution Factor: 12.0
```

 Copyright 2015 Advanced Analytical Technologies, Inc.

Sample: ID13507_72
Well Location: F12
Created: Tuesday, June 27, 2017 12:18:19 PM

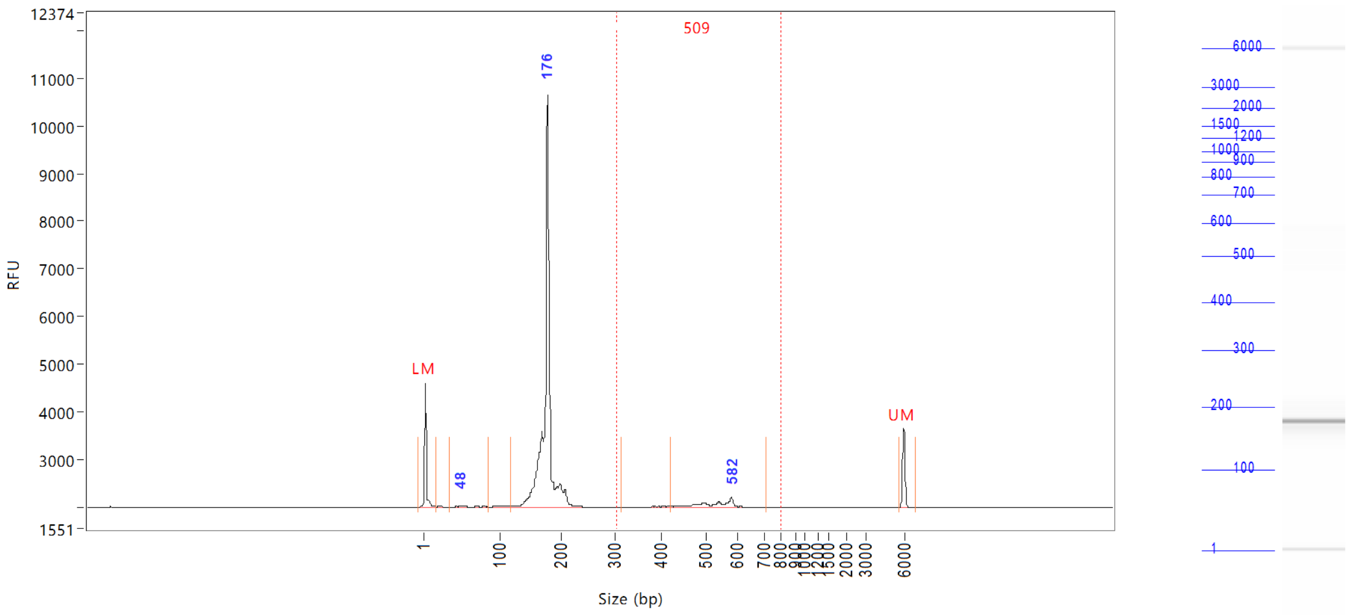

Peak	Size (bp)	Conc. (ng/uL)	From (bp)	To (bp)	Avg. Size (bp)	CV%	RFU	Corr. Peak Area
1	1 (LM)	0.0184	0	16	1	212.94	2613	15.912
2	48	0.0205	34	84	58	23.85	33	1.478
3	176	1.4839	118	313	174	8.96	8649	107.125
4	582	0.0960	421	709	531	10.02	209	6.932
5	6000 (UM)	0.0073	5625	6984	5977	1.88	1660	6.307

TIC:	1.6004	ng/uL
TIM:	14.853	nmole/L
Total Conc.:	1.6594	ng/uL

Smear Analysis 300 bp to 800 bp 0.1148 ng/ul 6.9 %Total 0.371 nmole/L 509 Avg. Size (b.p.) 16.32 %CV

```
Sample Peak Width (sec): 50   Sample Min Peak Height: 25    Sample Baseline V to V?: Y    Sample Baseline V to V pts: 3
Sample Filter: Binomial       # of Pts for Filter: 3        Sample Start Region (min): 0  Sample End Region (min): 50
Manual Baseline Start (min): 10     Manual Baseline End (min): 48
Marker Peak Width (sec): 5    Marker Min Peak Height: 200   Marker Baseline V to V?: Y    Marker Baseline V to V pts: 3
Lower Marker Selection: First Peak > 200 RFU                Upper Marker Selection: Last Peak > 200 RFU
Ladder Size (bp): 1, 100, 200, 300, 400, 500, 600, 700, 800, 900, 1000, 1200, 1500, 2000, 3000, 6000
Quantification Using: Ladder          Final Concentration (ng/uL): 0.0830          Dilution Factor: 12.0
```

 Copyright 2015 Advanced Analytical Technologies, Inc.

Sample: ID13507_73
Well Location: G1
Created: Tuesday, June 27, 2017 12:18:19 PM

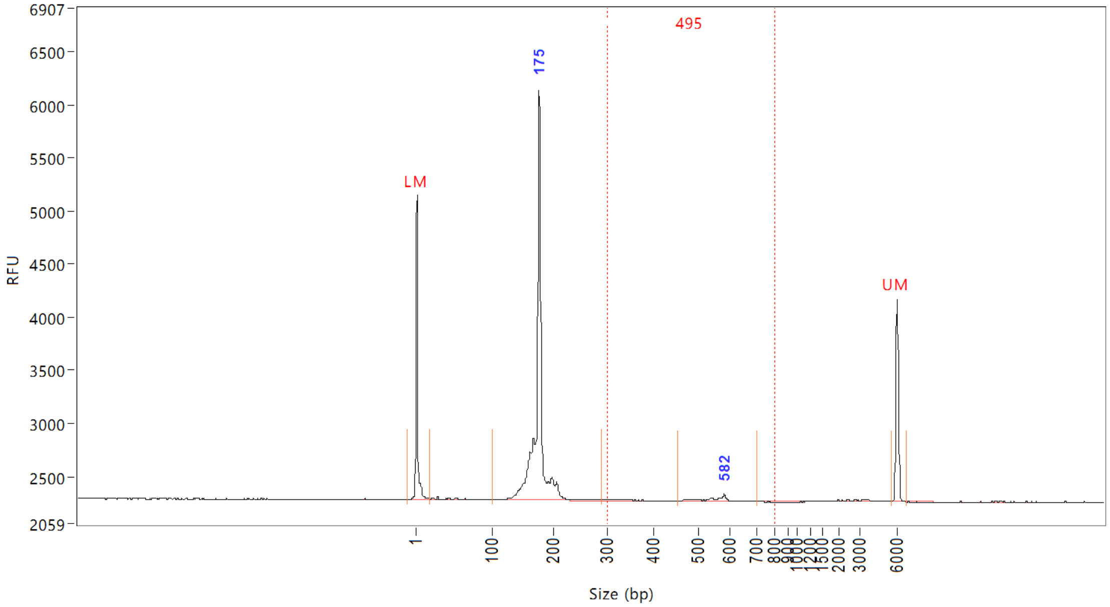

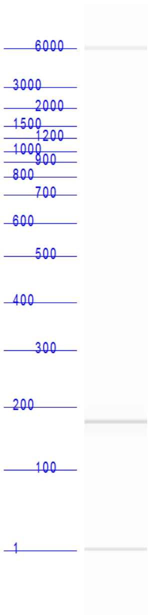

Peak	Size (bp)	Conc. (ng/uL)	From (bp)	To (bp)	Avg. Size (bp)	CV%	RFU	Corr. Peak Area
1	1 (LM)	0.0184	0	20	1	208.48	2865	16.804
2	175	0.6001	100	289	173	9.23	3853	45.752
3	582	0.0161	456	700	541	7.25	64	1.229
4	6000 (UM)	0.0077	5545	6718	5979	1.63	1898	7.086

TIC:	0.6162	ng/uL
TIM:	5.675	nmole/L
Total Conc.:	0.6503	ng/uL

Smear Analysis 300 bp to 800 bp 0.0208 ng/ul 3.2 %Total 0.069 nmole/L 495 Avg. Size (b.p.) 18.86 %CV

```
Sample Peak Width (sec): 50   Sample Min Peak Height: 25    Sample Baseline V to V?: Y    Sample Baseline V to V pts: 3
Sample Filter: Binomial       # of Pts for Filter: 3        Sample Start Region (min): 0  Sample End Region (min): 50
Manual Baseline Start (min): 10     Manual Baseline End (min): 48
Marker Peak Width (sec): 5    Marker Min Peak Height: 200   Marker Baseline V to V?: Y    Marker Baseline V to V pts: 3
Lower Marker Selection: First Peak > 200 RFU                Upper Marker Selection: Last Peak > 200 RFU
Ladder Size (bp): 1, 100, 200, 300, 400, 500, 600, 700, 800, 900, 1000, 1200, 1500, 2000, 3000, 6000
Quantification Using: Ladder          Final Concentration (ng/uL): 0.0830          Dilution Factor: 12.0
```

 Copyright 2015 Advanced Analytical Technologies, Inc.

Sample: ID13507_74
Well Location: G2
Created: Tuesday, June 27, 2017 12:18:19 PM

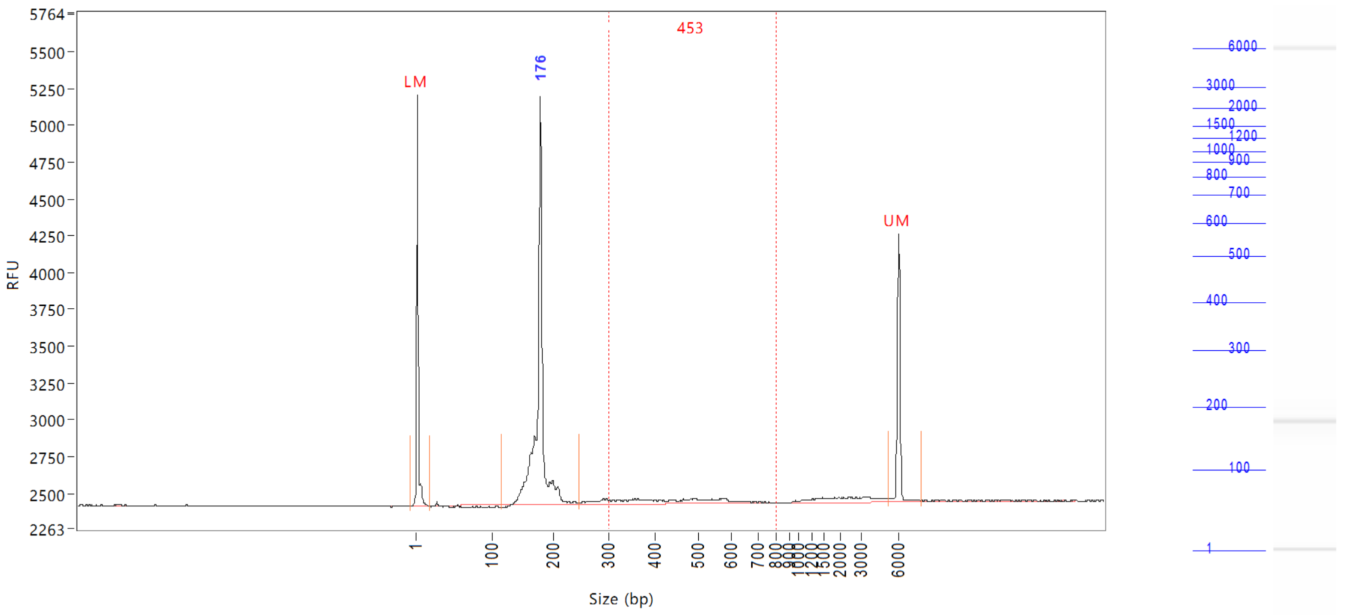

Peak	Size (bp)	Conc. (ng/uL)	From (bp)	To (bp)	Avg. Size (bp)	CV%	RFU	Corr. Peak Area
1	1 (LM)	0.0184	0	19	1	196.73	2786	16.026
2	176	0.4924	113	246	174	8.24	2767	35.803
3	6000 (UM)	0.0084	5170	7729	5992	3.94	1821	7.333

TIC:	0.4924	ng/uL
TIM:	4.604	nmole/L
Total Conc.:	0.6320	ng/uL

Smear Analysis 300 bp to 800 bp 0.0768 ng/ul 12.2 %Total 0.279 nmole/L 453 Avg. Size (b.p.) 23.19 %CV

Sample Peak Width (sec): 50 Sample Min Peak Height: 25 Sample Baseline V to V?: Y Sample Baseline V to V pts: 3
Sample Filter: Binomial # of Pts for Filter: 3 Sample Start Region (min): 0 Sample End Region (min): 50
Manual Baseline Start (min): 10 Manual Baseline End (min): 48
Marker Peak Width (sec): 5 Marker Min Peak Height: 200 Marker Baseline V to V?: Y Marker Baseline V to V pts: 3
Lower Marker Selection: First Peak > 200 RFU Upper Marker Selection: Last Peak > 200 RFU
Ladder Size (bp): 1, 100, 200, 300, 400, 500, 600, 700, 800, 900, 1000, 1200, 1500, 2000, 3000, 6000
Quantification Using: Ladder Final Concentration (ng/uL): 0.0830 Dilution Factor: 12.0

Sample: ID13507_75
Well Location: G3
Created: Tuesday, June 27, 2017 12:18:19 PM

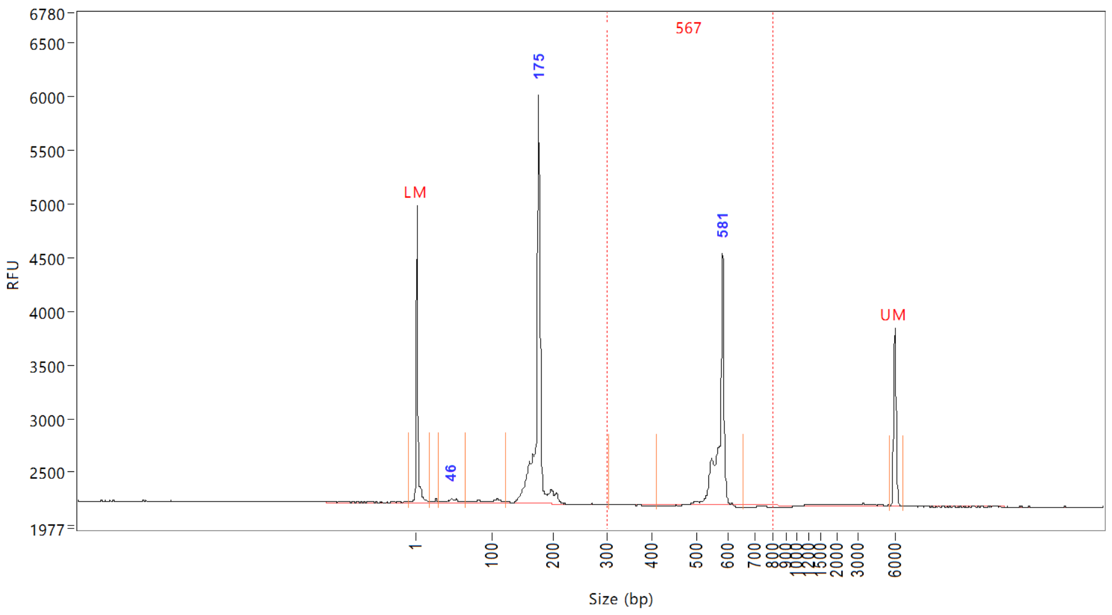

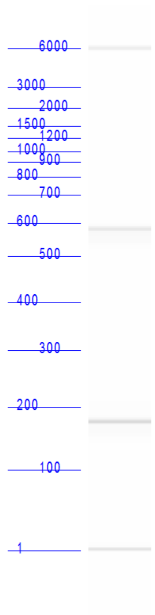

Peak	Size (bp)	Conc. (ng/uL)	From (bp)	To (bp)	Avg. Size (bp)	CV%	RFU	Corr. Peak Area
1	1 (LM)	0.0184	0	19	1	200.99	2765	16.473
2	46	0.0206	31	65	49	18.30	46	1.539
3	175	0.5235	121	304	172	6.70	3804	39.128
4	581	0.2691	409	654	567	3.46	2344	20.114
5	6000 (UM)	0.0070	5625	6638	5971	1.65	1656	6.239

TIC:	0.8132	ng/uL
TIM:	6.402	nmole/L
Total Conc.:	0.8638	ng/uL

Smear Analysis 300 bp to 800 bp 0.2691 ng/ul 31.2 %Total 0.781 nmole/L 567 Avg. Size (b.p.) 3.46 %CV

```
Sample Peak Width (sec): 50   Sample Min Peak Height: 25    Sample Baseline V to V?: Y    Sample Baseline V to V pts: 3
Sample Filter: Binomial       # of Pts for Filter: 3        Sample Start Region (min): 0  Sample End Region (min): 50
Manual Baseline Start (min): 10     Manual Baseline End (min): 48
Marker Peak Width (sec): 5    Marker Min Peak Height: 200   Marker Baseline V to V?: Y    Marker Baseline V to V pts: 3
Lower Marker Selection: First Peak > 200 RFU                Upper Marker Selection: Last Peak > 200 RFU
Ladder Size (bp): 1, 100, 200, 300, 400, 500, 600, 700, 800, 900, 1000, 1200, 1500, 2000, 3000, 6000
Quantification Using: Ladder          Final Concentration (ng/uL): 0.0830          Dilution Factor: 12.0
```

 Copyright 2015 Advanced Analytical Technologies, Inc.

Sample: ID13507_76
Well Location: G4
Created: Tuesday, June 27, 2017 12:18:19 PM

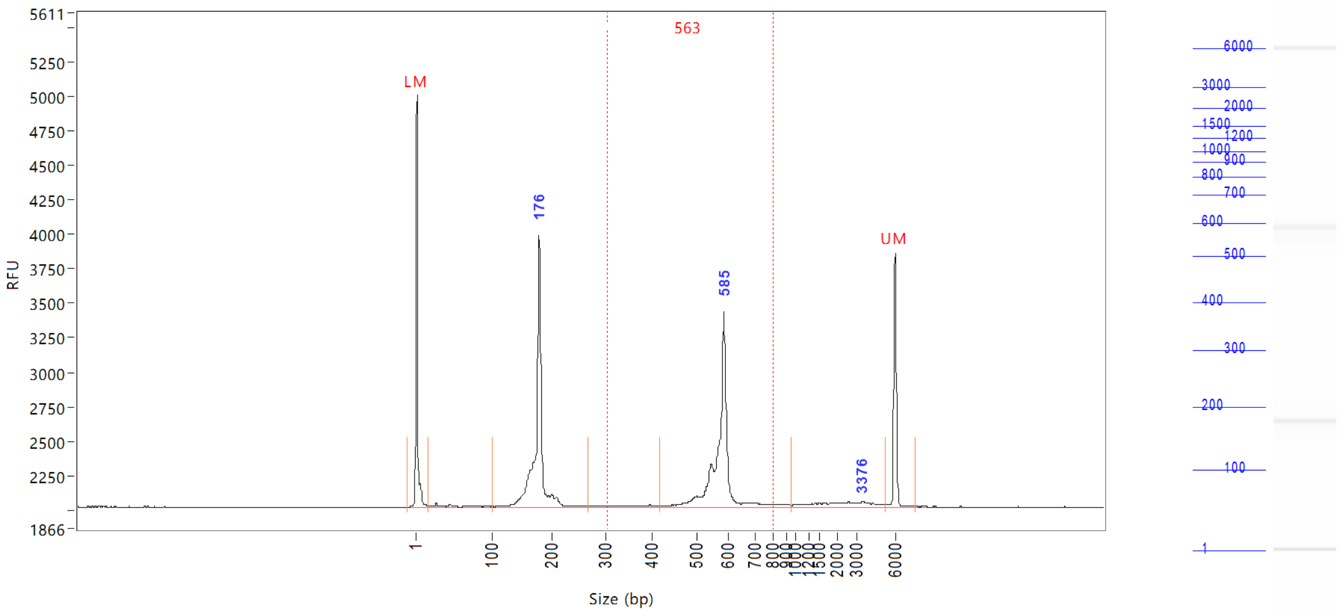

Peak	Size (bp)	Conc. (ng/uL)	From (bp)	To (bp)	Avg. Size (bp)	CV%	RFU	Corr. Peak Area
1	1 (LM)	0.0184	0	18	1	209.18	2993	17.603
2	176	0.3387	100	266	173	9.85	1978	27.054
3	585	0.2895	417	952	578	11.23	1426	23.121
4	3376	0.0436	952	5277	2451	47.05	42	3.479
5	6000 (UM)	0.0076	5277	7622	5998	3.37	1842	7.325

TIC:	0.6718	ng/uL
TIM:	3.993	nmole/L
Total Conc.:	0.7051	ng/uL

Smear Analysis 300 bp to 800 bp 0.2959 ng/ul 42.0 %Total 0.866 nmole/L 563 Avg. Size (b.p.) 11.57 %CV

```
Sample Peak Width (sec): 50   Sample Min Peak Height: 25    Sample Baseline V to V?: Y    Sample Baseline V to V pts: 3
Sample Filter: Binomial       # of Pts for Filter: 3        Sample Start Region (min): 0  Sample End Region (min): 50
Manual Baseline Start (min): 10     Manual Baseline End (min): 48
Marker Peak Width (sec): 5    Marker Min Peak Height: 200   Marker Baseline V to V?: Y    Marker Baseline V to V pts: 3
Lower Marker Selection: First Peak > 200 RFU                Upper Marker Selection: Last Peak > 200 RFU
Ladder Size (bp): 1, 100, 200, 300, 400, 500, 600, 700, 800, 900, 1000, 1200, 1500, 2000, 3000, 6000
Quantification Using: Ladder          Final Concentration (ng/uL): 0.0830          Dilution Factor: 12.0
```


Copyright 2015 Advanced Analytical Technologies, Inc.

Sample: ID13507_77
Well Location: G5
Created: Tuesday, June 27, 2017 12:18:19 PM

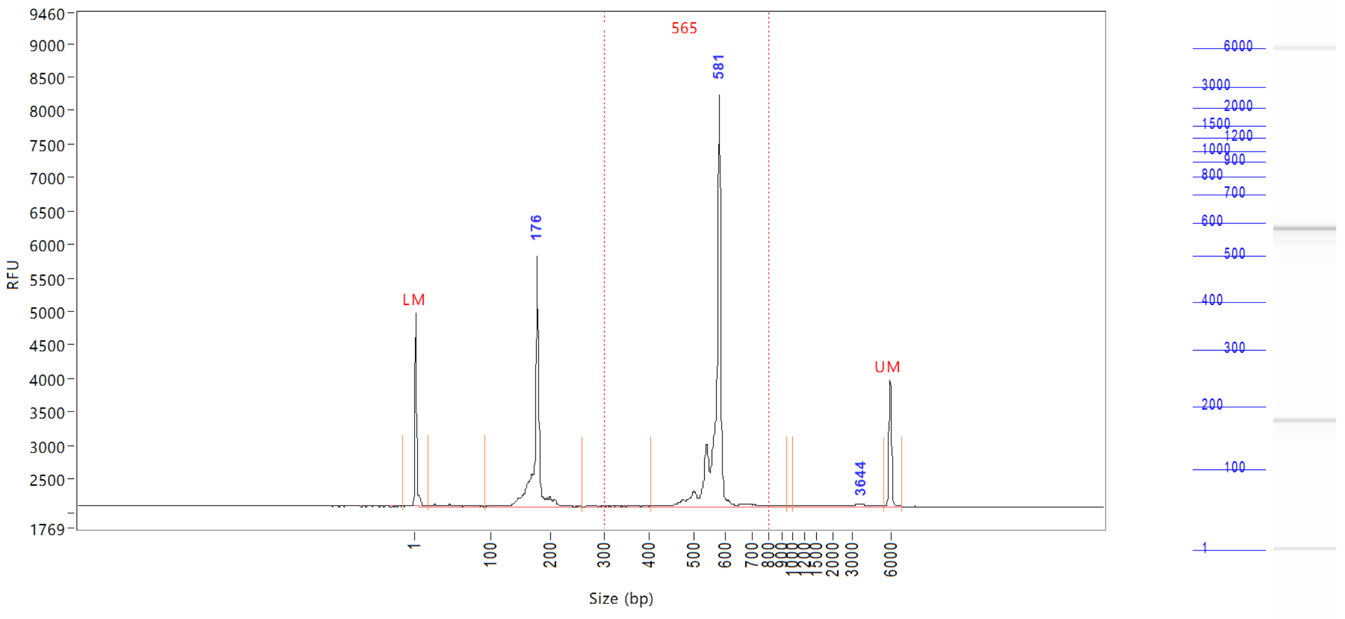

Peak	Size (bp)	Conc. (ng/uL)	From (bp)	To (bp)	Avg. Size (bp)	CV%	RFU	Corr. Peak Area
1	1 (LM)	0.0184	0	19	1	206.03	2888	16.916
2	176	0.5291	92	258	173	8.41	3735	40.609
3	581	0.7457	403	956	568	7.80	6143	57.228
4	3644	0.0339	1023	5545	2782	44.12	37	2.605
5	6000 (UM)	0.0079	5545	6984	5987	2.26	1889	7.320

TIC:	1.3088	ng/uL
TIM:	7.061	nmole/L
Total Conc.:	1.3313	ng/uL

Smear Analysis	300 bp to 800 bp	0.7473 ng/ul	56.1 %Total	2.178 nmole/L	565 Avg. Size (b.p.)	7.52 %CV

Sample Peak Width (sec): 50 Sample Min Peak Height: 25 Sample Baseline V to V?: Y Sample Baseline V to V pts: 3
Sample Filter: Binomial # of Pts for Filter: 3 Sample Start Region (min): 0 Sample End Region (min): 50
Manual Baseline Start (min): 10 Manual Baseline End (min): 48
Marker Peak Width (sec): 5 Marker Min Peak Height: 200 Marker Baseline V to V?: Y Marker Baseline V to V pts: 3
Lower Marker Selection: First Peak > 200 RFU Upper Marker Selection: Last Peak > 200 RFU
Ladder Size (bp): 1, 100, 200, 300, 400, 500, 600, 700, 800, 900, 1000, 1200, 1500, 2000, 3000, 6000
Quantification Using: Ladder Final Concentration (ng/uL): 0.0830 Dilution Factor: 12.0

 Copyright 2015 Advanced Analytical Technologies, Inc.

Sample: ID13507_78
Well Location: G6
Created: Tuesday, June 27, 2017 12:18:19 PM

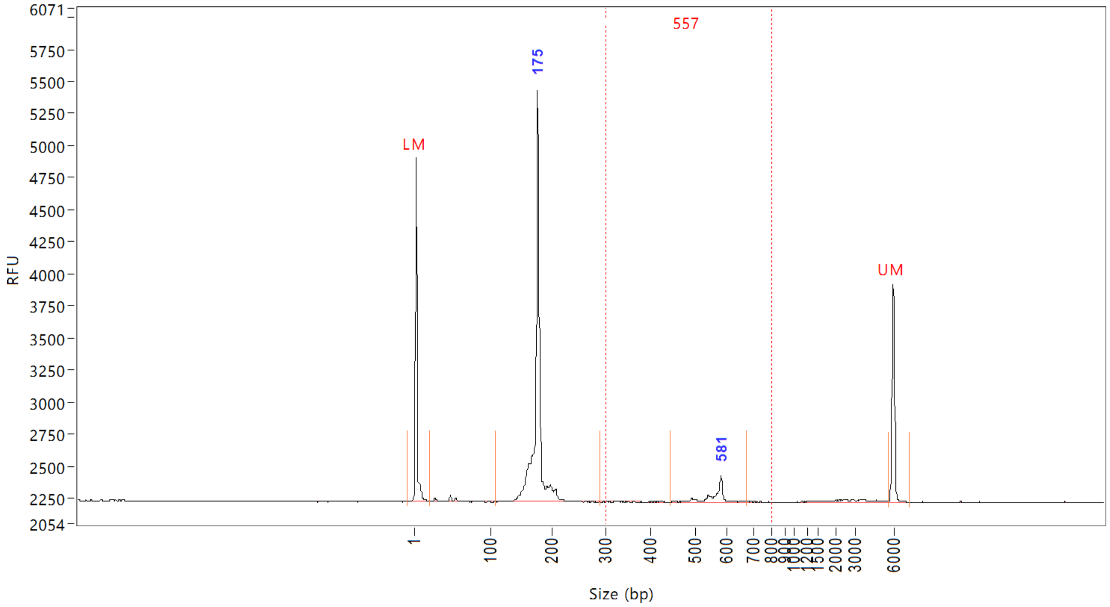

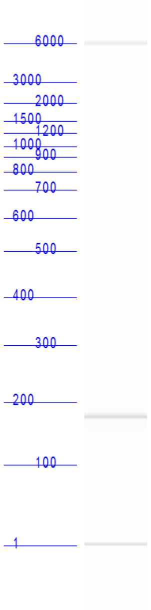

Peak	Size (bp)	Conc. (ng/uL)	From (bp)	To (bp)	Avg. Size (bp)	CV%	RFU	Corr. Peak Area
1	1 (LM)	0.0184	0	20	1	188.22	2682	15.598
2	175	0.4701	108	291	174	6.82	3204	33.271
3	581	0.0370	444	670	558	6.78	208	2.616
4	6000 (UM)	0.0078	5572	7303	5978	2.27	1695	6.612
	TIC:	0.5071	ng/uL					
	TIM:	4.515	nmole/L					
	Total Conc.:	0.5378	ng/uL					

Smear Analysis	300 bp to 800 bp	0.0386 ng/ul	7.2 %Total	0.114 nmole/L	557 Avg. Size (b.p.)	9.24 %CV

```
Sample Peak Width (sec): 50   Sample Min Peak Height: 25    Sample Baseline V to V?: Y    Sample Baseline V to V pts: 3
Sample Filter: Binomial       # of Pts for Filter: 3        Sample Start Region (min): 0  Sample End Region (min): 50
Manual Baseline Start (min): 10     Manual Baseline End (min): 48
Marker Peak Width (sec): 5    Marker Min Peak Height: 200   Marker Baseline V to V?: Y    Marker Baseline V to V pts: 3
Lower Marker Selection: First Peak > 200 RFU                Upper Marker Selection: Last Peak > 200 RFU
Ladder Size (bp): 1, 100, 200, 300, 400, 500, 600, 700, 800, 900, 1000, 1200, 1500, 2000, 3000, 6000
Quantification Using: Ladder          Final Concentration (ng/uL): 0.0830         Dilution Factor: 12.0
```

 Copyright 2015 Advanced Analytical Technologies, Inc.

Sample: ID13507_79
Well Location: G7
Created: Tuesday, June 27, 2017 12:18:19 PM

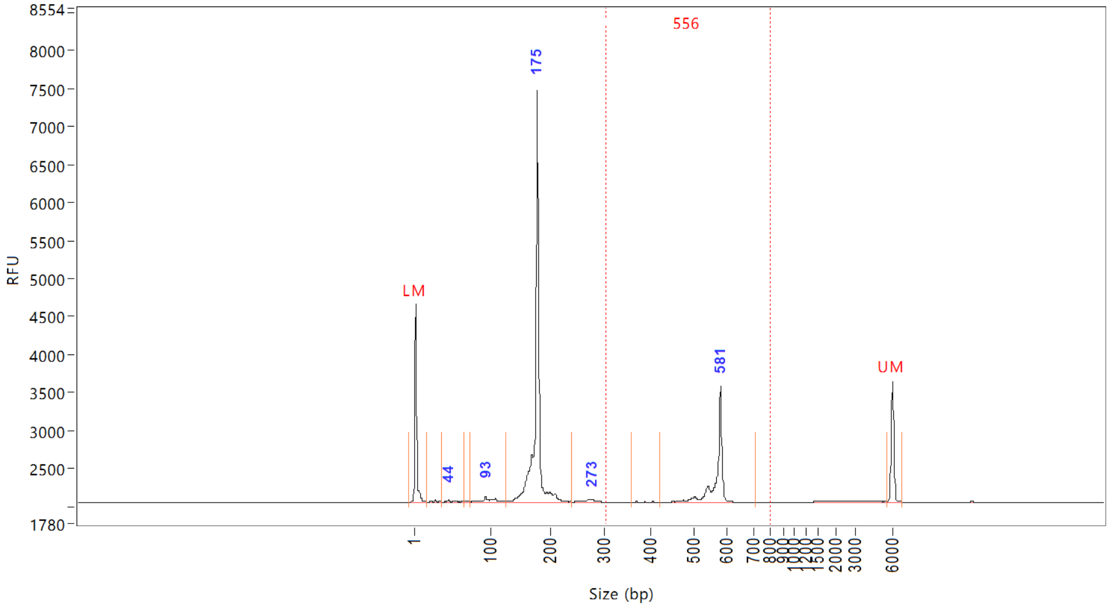

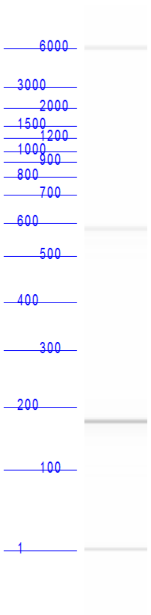

Peak	Size (bp)	Conc. (ng/uL)	From (bp)	To (bp)	Avg. Size (bp)	CV%	RFU	Corr. Peak Area
1	1 (LM)	0.0184	0	18	1	217.37	2616	15.639
2	44	0.0102	36	66	50	15.75	39	0.726
3	93	0.0288	74	126	98	12.06	84	2.045
4	175	0.7190	126	238	174	6.57	5412	51.013
5	273	0.0173	238	360	279	9.54	36	1.226
6	581	0.1921	421	717	562	5.63	1521	13.628
7	6000 (UM)	0.0074	5598	6824	5994	1.90	1592	6.296

TIC:	0.9674	ng/uL
TIM:	8.283	nmole/L
Total Conc.:	0.9928	ng/uL

Smear Analysis	300 bp to 800 bp	0.1983 ng/ul	20.0 %Total	0.587 nmole/L	556 Avg. Size (b.p.)	8.35 %CV

Sample Peak Width (sec): 50 Sample Min Peak Height: 25 Sample Baseline V to V?: Y Sample Baseline V to V pts: 3
Sample Filter: Binomial # of Pts for Filter: 3 Sample Start Region (min): 0 Sample End Region (min): 50
Manual Baseline Start (min): 10 Manual Baseline End (min): 48
Marker Peak Width (sec): 5 Marker Min Peak Height: 200 Marker Baseline V to V?: Y Marker Baseline V to V pts: 3
Lower Marker Selection: First Peak > 200 RFU Upper Marker Selection: Last Peak > 200 RFU
Ladder Size (bp): 1, 100, 200, 300, 400, 500, 600, 700, 800, 900, 1000, 1200, 1500, 2000, 3000, 6000
Quantification Using: Ladder Final Concentration (ng/uL): 0.0830 Dilution Factor: 12.0

 Copyright 2015 Advanced Analytical Technologies, Inc.

Sample: ID13507_80
Well Location: G8
Created: Tuesday, June 27, 2017 12:18:19 PM

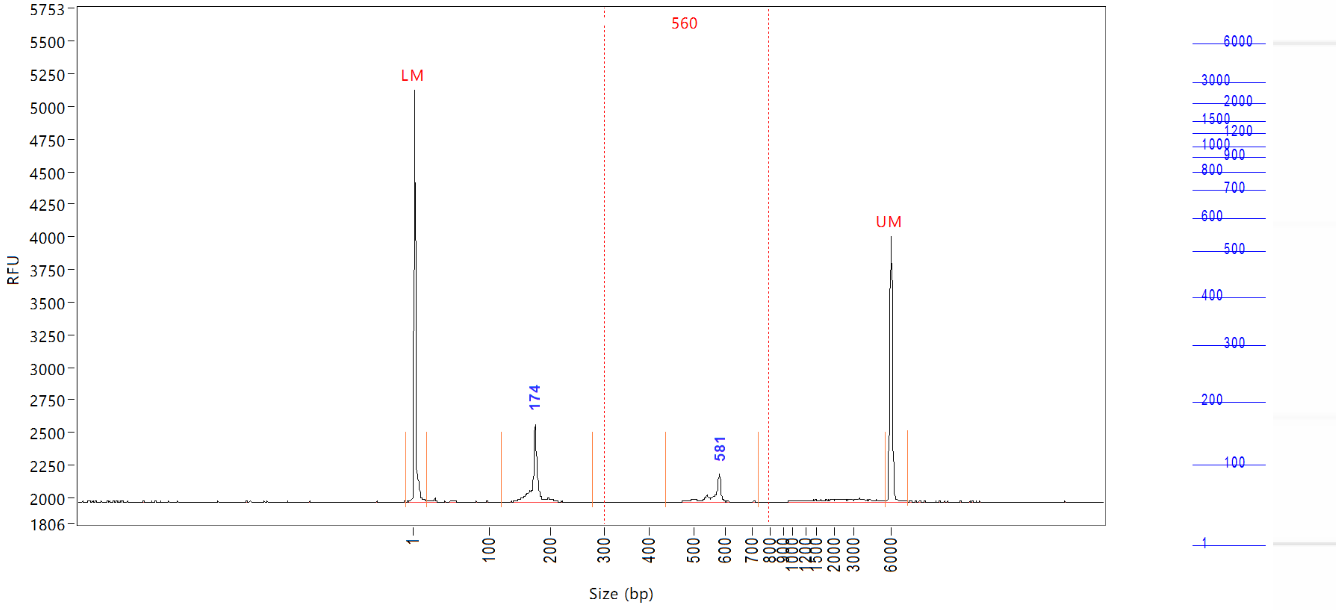

Peak	Size (bp)	Conc. (ng/uL)	From (bp)	To (bp)	Avg. Size (bp)	CV%	RFU	Corr. Peak Area
1	1 (LM)	0.0184	0	19	1	192.88	3153	18.306
2	174	0.0838	119	278	172	6.81	594	6.958
3	581	0.0274	438	739	560	5.51	216	2.272
4	6000 (UM)	0.0079	5545	7356	5976	2.12	2035	7.836
	TIC:	0.1111	ng/uL					
	TIM:	0.871	nmole/L					
	Total Conc.:	0.1356	ng/uL					

Smear Analysis	300 bp to 800 bp	0.0274 ng/ul	20.2 %Total	0.080 nmole/L	560 Avg. Size (b.p.)	5.69 %CV

```
Sample Peak Width (sec): 50   Sample Min Peak Height: 25    Sample Baseline V to V?: Y    Sample Baseline V to V pts: 3
Sample Filter: Binomial       # of Pts for Filter: 3        Sample Start Region (min): 0  Sample End Region (min): 50
Manual Baseline Start (min): 10     Manual Baseline End (min): 48
Marker Peak Width (sec): 5    Marker Min Peak Height: 200   Marker Baseline V to V?: Y    Marker Baseline V to V pts: 3
Lower Marker Selection: First Peak > 200 RFU                Upper Marker Selection: Last Peak > 200 RFU
Ladder Size (bp): 1, 100, 200, 300, 400, 500, 600, 700, 800, 900, 1000, 1200, 1500, 2000, 3000, 6000
Quantification Using: Ladder          Final Concentration (ng/uL): 0.0830          Dilution Factor: 12.0
```

 Copyright 2015 Advanced Analytical Technologies, Inc.

Sample: ID13507_81
Well Location: G9
Created: Tuesday, June 27, 2017 12:18:19 PM

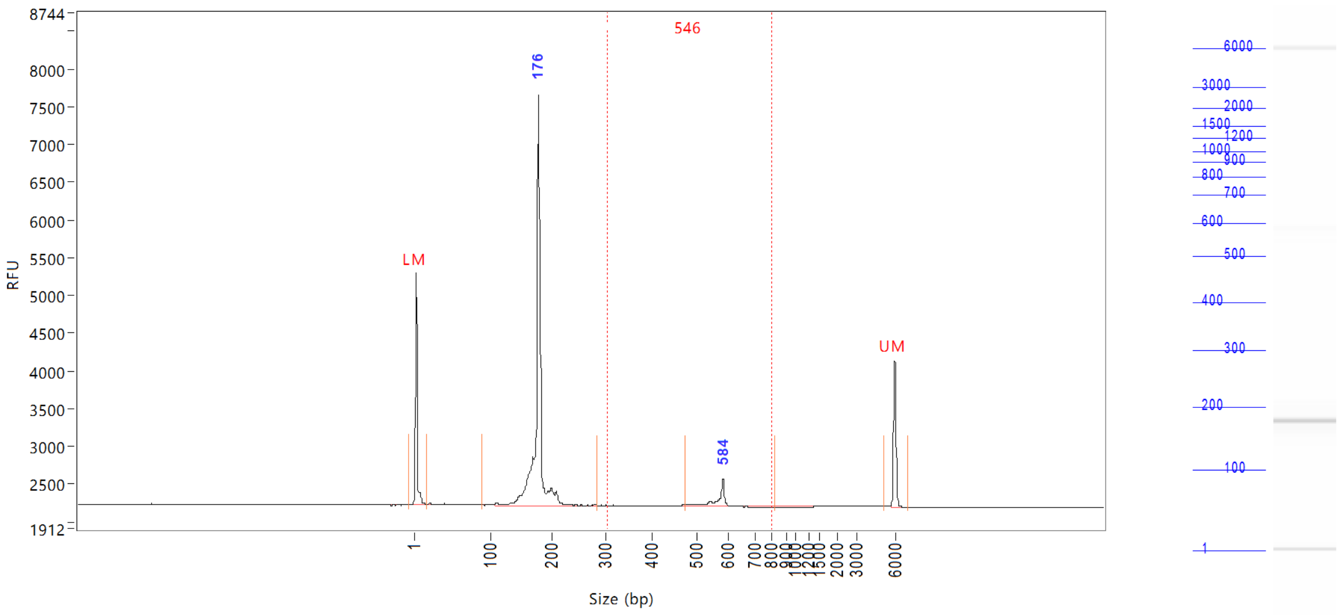

Peak	Size (bp)	Conc. (ng/uL)	From (bp)	To (bp)	Avg. Size (bp)	CV%	RFU	Corr. Peak Area
1	1 (LM)	0.0184	0	16	1	159.28	3073	18.339
2	176	0.6990	88	282	174	8.40	5434	58.165
3	584	0.0443	475	813	561	5.52	365	3.685
4	6000 (UM)	0.0073	5170	7090	5985	1.74	1924	7.290

TIC:	0.7433	ng/uL
TIM:	6.641	nmole/L
Total Conc.:	0.7613	ng/uL

Smear Analysis	300 bp to 800 bp	0.0481 ng/ul	6.3 %Total	0.145 nmole/L	546 Avg. Size (b.p.)	10.79 %CV

```
Sample Peak Width (sec): 50   Sample Min Peak Height: 25    Sample Baseline V to V?: Y    Sample Baseline V to V pts: 3
Sample Filter: Binomial       # of Pts for Filter: 3        Sample Start Region (min): 0  Sample End Region (min): 50
Manual Baseline Start (min): 10     Manual Baseline End (min): 48
Marker Peak Width (sec): 5    Marker Min Peak Height: 200   Marker Baseline V to V?: Y    Marker Baseline V to V pts: 3
Lower Marker Selection: First Peak > 200 RFU                Upper Marker Selection: Last Peak > 200 RFU
Ladder Size (bp): 1, 100, 200, 300, 400, 500, 600, 700, 800, 900, 1000, 1200, 1500, 2000, 3000, 6000
Quantification Using: Ladder          Final Concentration (ng/uL): 0.0830          Dilution Factor: 12.0
```

 Copyright 2015 Advanced Analytical Technologies, Inc.

Sample: ID13507_82
Well Location: G10
Created: Tuesday, June 27, 2017 12:18:19 PM

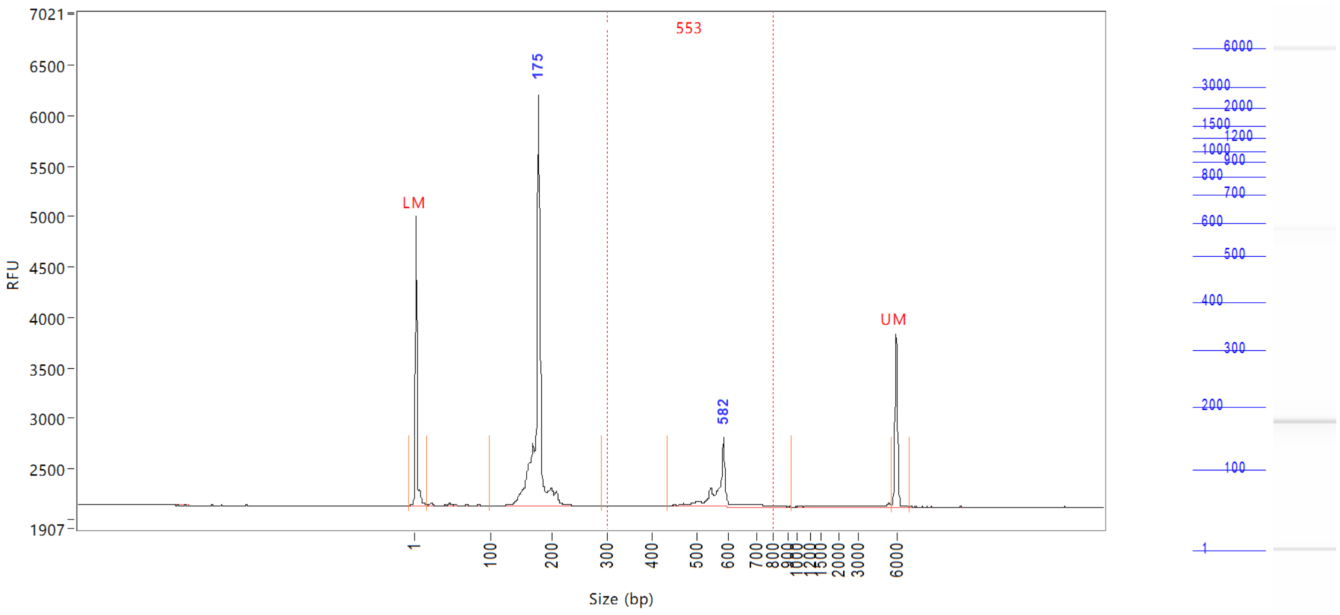

Peak	Size (bp)	Conc. (ng/uL)	From (bp)	To (bp)	Avg. Size (bp)	CV%	RFU	Corr. Peak Area
1	1 (LM)	0.0184	0	17	1	197.72	2872	17.133
2	175	0.6081	97	289	174	9.33	4076	47.269
3	582	0.1278	433	949	569	10.74	683	9.936
4	6000 (UM)	0.0071	5598	7037	5972	2.05	1722	6.655

TIC:	0.7359	ng/uL
TIM:	6.066	nmole/L
Total Conc.:	0.7790	ng/uL

Smear Analysis	300 bp to 800 bp	0.1361 ng/ul	17.5 %Total	0.405 nmole/L	553 Avg. Size (b.p.)	13.41 %CV

Sample Peak Width (sec): 50 Sample Min Peak Height: 25 Sample Baseline V to V?: Y Sample Baseline V to V pts: 3
Sample Filter: Binomial # of Pts for Filter: 3 Sample Start Region (min): 0 Sample End Region (min): 50
Manual Baseline Start (min): 10 Manual Baseline End (min): 48
Marker Peak Width (sec): 5 Marker Min Peak Height: 200 Marker Baseline V to V?: Y Marker Baseline V to V pts: 3
Lower Marker Selection: First Peak > 200 RFU Upper Marker Selection: Last Peak > 200 RFU
Ladder Size (bp): 1, 100, 200, 300, 400, 500, 600, 700, 800, 900, 1000, 1200, 1500, 2000, 3000, 6000
Quantification Using: Ladder Final Concentration (ng/uL): 0.0830 Dilution Factor: 12.0

Copyright 2015 Advanced Analytical Technologies, Inc.

Sample: ID13507_83
Well Location: G11
Created: Tuesday, June 27, 2017 12:18:19 PM

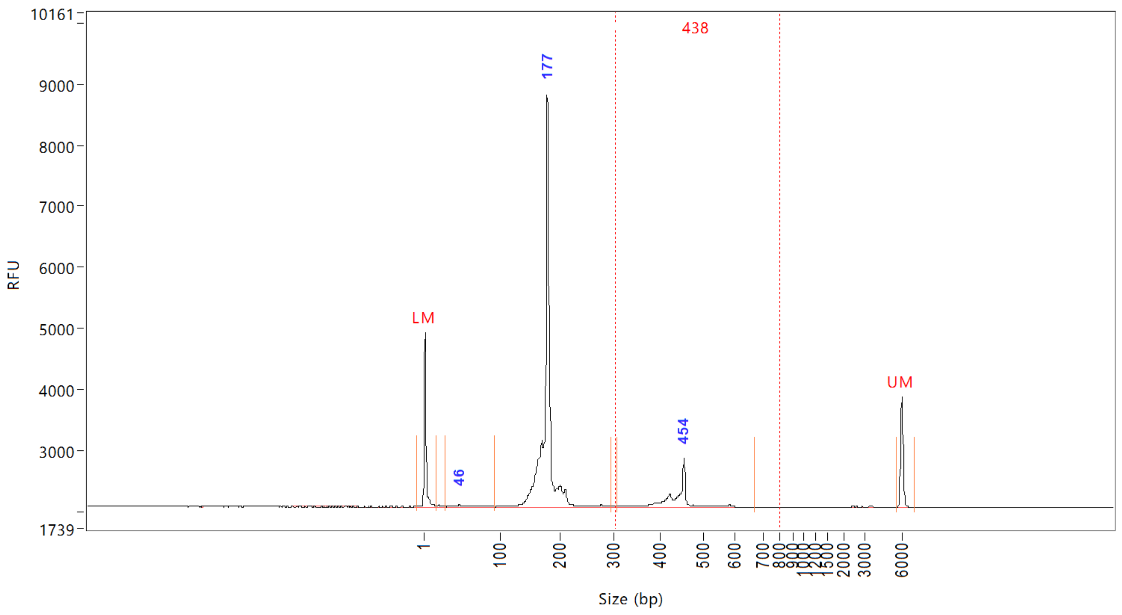

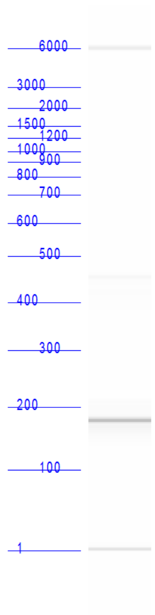

Peak	Size (bp)	Conc. (ng/uL)	From (bp)	To (bp)	Avg. Size (bp)	CV%	RFU	Corr. Peak Area
1	1 (LM)	0.0184	0	17	1	171.18	2842	17.186
2	46	0.0151	29	93	60	29.85	49	1.179
3	177	1.0557	93	295	175	9.51	6723	82.314
4	454	0.1618	308	668	439	10.50	802	12.619
5	6000 (UM)	0.0073	5545	6957	5980	1.75	1813	6.874

TIC:	1.2326	ng/uL
TIM:	10.938	nmole/L
Total Conc.:	1.2437	ng/uL

Smear Analysis 300 bp to 800 bp 0.1627 ng/ul 13.1 %Total 0.611 nmole/L 438 Avg. Size (b.p.) 10.74 %CV

Sample Peak Width (sec): 50 Sample Min Peak Height: 25 Sample Baseline V to V?: Y Sample Baseline V to V pts: 3
Sample Filter: Binomial # of Pts for Filter: 3 Sample Start Region (min): 0 Sample End Region (min): 50
Manual Baseline Start (min): 10 Manual Baseline End (min): 48
Marker Peak Width (sec): 5 Marker Min Peak Height: 200 Marker Baseline V to V?: Y Marker Baseline V to V pts: 3
Lower Marker Selection: First Peak > 200 RFU Upper Marker Selection: Last Peak > 200 RFU
Ladder Size (bp): 1, 100, 200, 300, 400, 500, 600, 700, 800, 900, 1000, 1200, 1500, 2000, 3000, 6000
Quantification Using: Ladder Final Concentration (ng/uL): 0.0830 Dilution Factor: 12.0

 Copyright 2015 Advanced Analytical Technologies, Inc.

Sample: ID13507_84
Well Location: G12
Created: Tuesday, June 27, 2017 12:18:19 PM

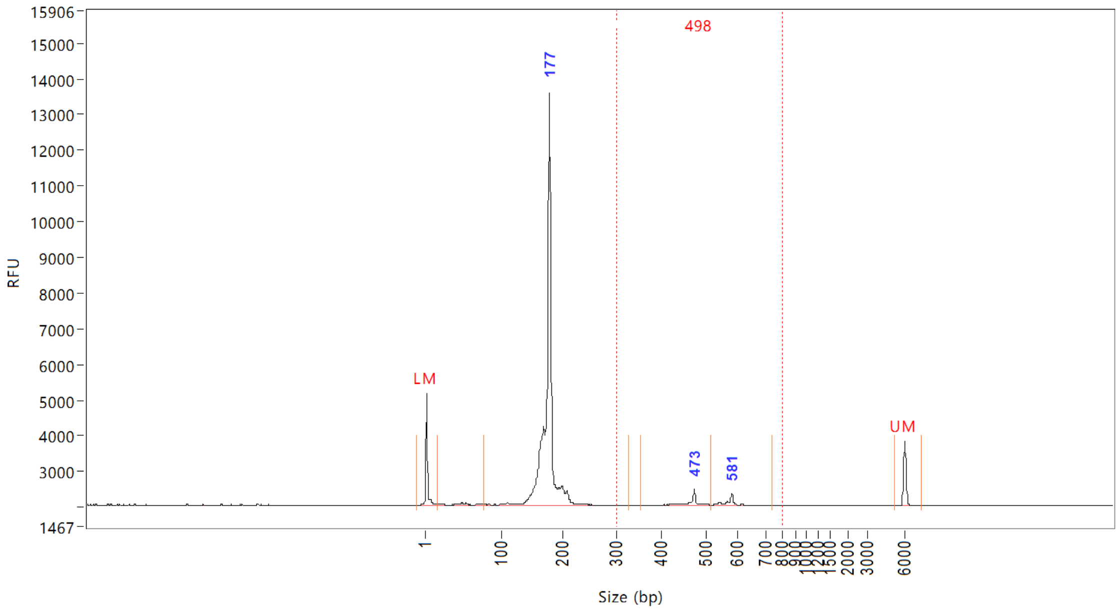

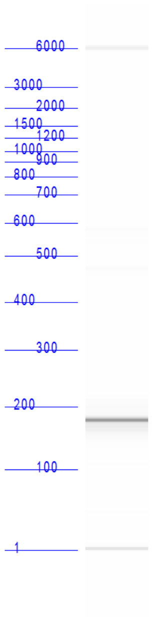

Peak	Size (bp)	Conc. (ng/uL)	From (bp)	To (bp)	Avg. Size (bp)	CV%	RFU	Corr. Peak Area
1	1 (LM)	0.0184	0	17	2	164.23	3120	18.799
2	177	1.6851	77	327	174	9.12	11533	143.726
3	473	0.0529	353	514	455	6.57	436	4.509
4	581	0.0391	514	739	567	3.68	312	3.334
5	6000 (UM)	0.0067	5143	7277	5974	1.77	1820	6.832

TIC:	1.7771	ng/uL
TIM:	15.906	nmole/L
Total Conc.:	1.8068	ng/uL

Smear Analysis 300 bp to 800 bp 0.0945 ng/ul 5.2 %Total 0.313 nmole/L 498 Avg. Size (b.p.) 13.44 %CV

```
Sample Peak Width (sec): 50   Sample Min Peak Height: 25    Sample Baseline V to V?: Y    Sample Baseline V to V pts: 3
Sample Filter: Binomial       # of Pts for Filter: 3        Sample Start Region (min): 0  Sample End Region (min): 50
Manual Baseline Start (min): 10     Manual Baseline End (min): 48
Marker Peak Width (sec): 5    Marker Min Peak Height: 200   Marker Baseline V to V?: Y    Marker Baseline V to V pts: 3
Lower Marker Selection: First Peak > 200 RFU                Upper Marker Selection: Last Peak > 200 RFU
Ladder Size (bp): 1, 100, 200, 300, 400, 500, 600, 700, 800, 900, 1000, 1200, 1500, 2000, 3000, 6000
Quantification Using: Ladder          Final Concentration (ng/uL): 0.0830          Dilution Factor: 12.0
```

 Copyright 2015 Advanced Analytical Technologies, Inc.

Sample: ID13507_85
Well Location: H1
Created: Tuesday, June 27, 2017 12:18:19 PM

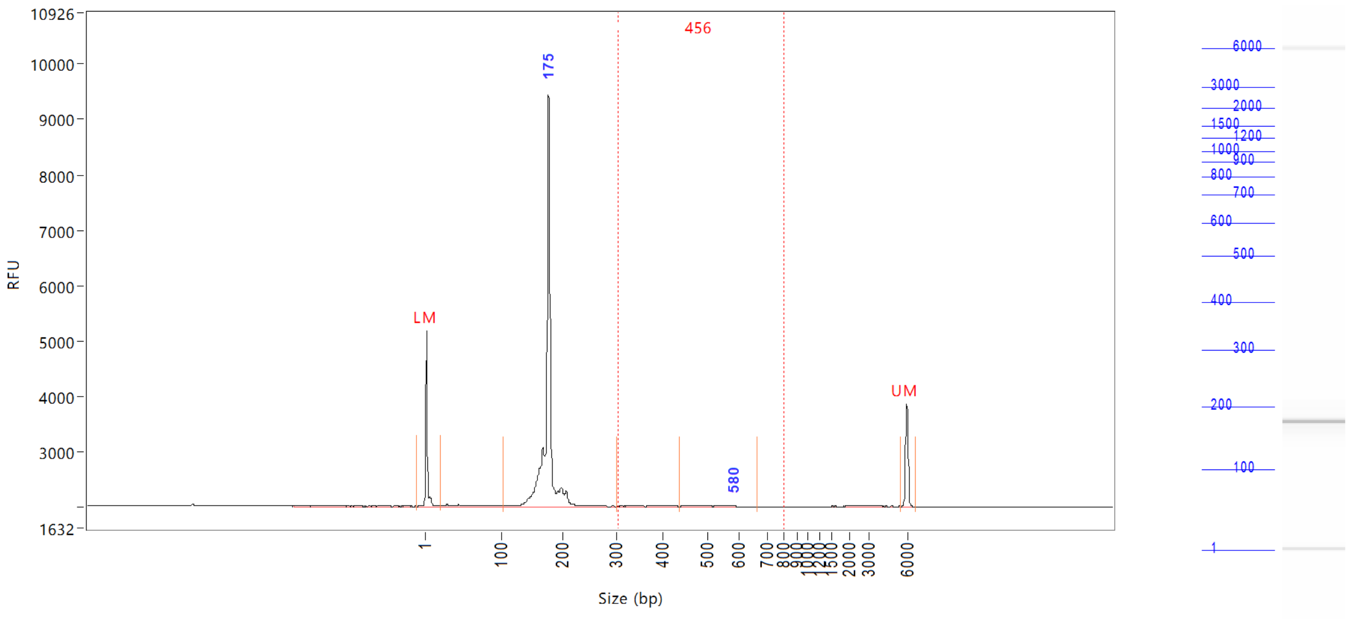

Peak	Size (bp)	Conc. (ng/uL)	From (bp)	To (bp)	Avg. Size (bp)	CV%	RFU	Corr. Peak Area
1	1 (LM)	0.0184	0	21	1	209.26	3166	18.569
2	175	0.9587	102	299	173	8.72	7421	80.769
3	580	0.0152	437	668	518	9.43	31	1.278
4	6000 (UM)	0.0069	5545	6665	5972	1.75	1846	7.022

TIC:	0.9739	ng/uL
TIM:	9.064	nmole/L
Total Conc.:	1.0216	ng/uL

Smear Analysis	300 bp to 800 bp	0.0266 ng/ul	2.6 %Total	0.096 nmole/L	456 Avg. Size (b.p.)	19.17 %CV

```
Sample Peak Width (sec): 50   Sample Min Peak Height: 25    Sample Baseline V to V?: Y    Sample Baseline V to V pts: 3
Sample Filter: Binomial       # of Pts for Filter: 3        Sample Start Region (min): 0  Sample End Region (min): 50
Manual Baseline Start (min): 10     Manual Baseline End (min): 48
Marker Peak Width (sec): 5    Marker Min Peak Height: 200   Marker Baseline V to V?: Y    Marker Baseline V to V pts: 3
Lower Marker Selection: First Peak > 200 RFU                Upper Marker Selection: Last Peak > 200 RFU
Ladder Size (bp): 1, 100, 200, 300, 400, 500, 600, 700, 800, 900, 1000, 1200, 1500, 2000, 3000, 6000
Quantification Using: Ladder          Final Concentration (ng/uL): 0.0830          Dilution Factor: 12.0
```

 Copyright 2015 Advanced Analytical Technologies, Inc.

Sample: ID13507_86
Well Location: H2
Created: Tuesday, June 27, 2017 12:18:19 PM

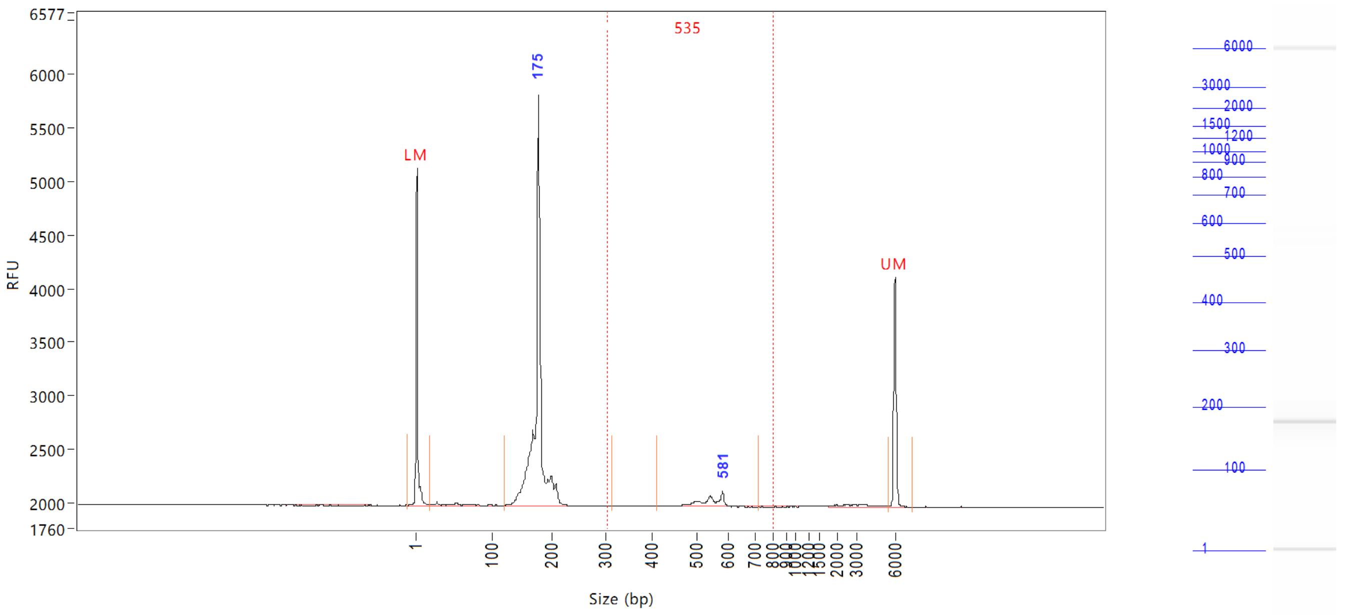

Peak	Size (bp)	Conc. (ng/uL)	From (bp)	To (bp)	Avg. Size (bp)	CV%	RFU	Corr. Peak Area
1	1 (LM)	0.0184	0	20	1	197.83	3154	18.329
2	175	0.5977	121	313	173	7.88	3835	49.702
3	581	0.0403	412	717	536	6.68	138	3.354
4	6000 (UM)	0.0081	5545	7383	5978	1.81	2152	8.130

TIC:	0.6380	ng/uL
TIM:	5.721	nmole/L
Total Conc.:	0.6682	ng/uL

Smear Analysis 300 bp to 800 bp 0.0405 ng/ul 6.1 %Total 0.124 nmole/L 535 Avg. Size (b.p.) 6.96 %CV

Sample Peak Width (sec): 50 Sample Min Peak Height: 25 Sample Baseline V to V?: Y Sample Baseline V to V pts: 3
Sample Filter: Binomial # of Pts for Filter: 3 Sample Start Region (min): 0 Sample End Region (min): 50
Manual Baseline Start (min): 10 Manual Baseline End (min): 48
Marker Peak Width (sec): 5 Marker Min Peak Height: 200 Marker Baseline V to V?: Y Marker Baseline V to V pts: 3
Lower Marker Selection: First Peak > 200 RFU Upper Marker Selection: Last Peak > 200 RFU
Ladder Size (bp): 1, 100, 200, 300, 400, 500, 600, 700, 800, 900, 1000, 1200, 1500, 2000, 3000, 6000
Quantification Using: Ladder Final Concentration (ng/uL): 0.0830 Dilution Factor: 12.0

 Copyright 2015 Advanced Analytical Technologies, Inc.

Sample: ID13507_87
Well Location: H3
Created: Tuesday, June 27, 2017 12:18:19 PM

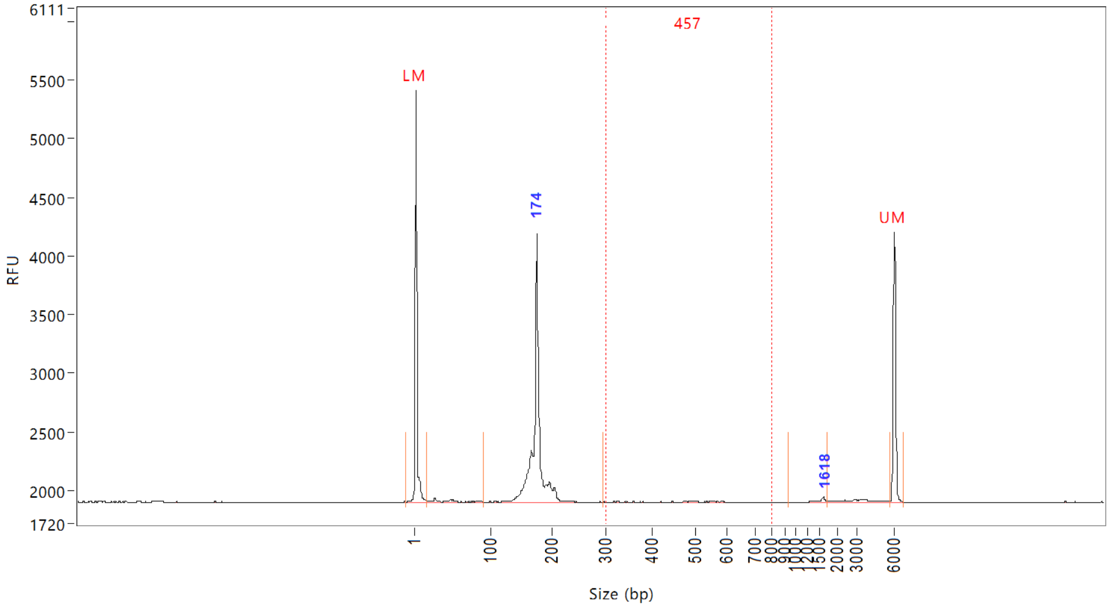

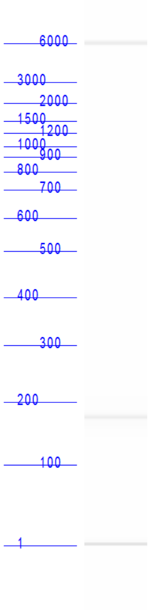

Peak	Size (bp)	Conc. (ng/uL)	From (bp)	To (bp)	Avg. Size (bp)	CV%	RFU	Corr. Peak Area
1	1 (LM)	0.0184	0	18	2	167.52	3509	20.951
2	174	0.3203	90	294	173	9.30	2295	30.446
3	1618	0.0041	934	1726	1497	10.89	45	0.391
4	6000 (UM)	0.0076	5625	6745	5983	1.76	2306	8.721

TIC:	0.3244	ng/uL
TIM:	3.028	nmole/L
Total Conc.:	0.3607	ng/uL

Smear Analysis	300 bp to 800 bp	0.0101 ng/ul	2.8 %Total	0.036 nmole/L	457 Avg. Size (b.p.)	19.37 %CV

Sample Peak Width (sec): 50 Sample Min Peak Height: 25 Sample Baseline V to V?: Y Sample Baseline V to V pts: 3
Sample Filter: Binomial # of Pts for Filter: 3 Sample Start Region (min): 0 Sample End Region (min): 50
Manual Baseline Start (min): 10 Manual Baseline End (min): 48
Marker Peak Width (sec): 5 Marker Min Peak Height: 200 Marker Baseline V to V?: Y Marker Baseline V to V pts: 3
Lower Marker Selection: First Peak > 200 RFU Upper Marker Selection: Last Peak > 200 RFU
Ladder Size (bp): 1, 100, 200, 300, 400, 500, 600, 700, 800, 900, 1000, 1200, 1500, 2000, 3000, 6000
Quantification Using: Ladder Final Concentration (ng/uL): 0.0830 Dilution Factor: 12.0

Copyright 2015 Advanced Analytical Technologies, Inc.

Sample: ID13507_88
Well Location: H4
Created: Tuesday, June 27, 2017 12:18:19 PM

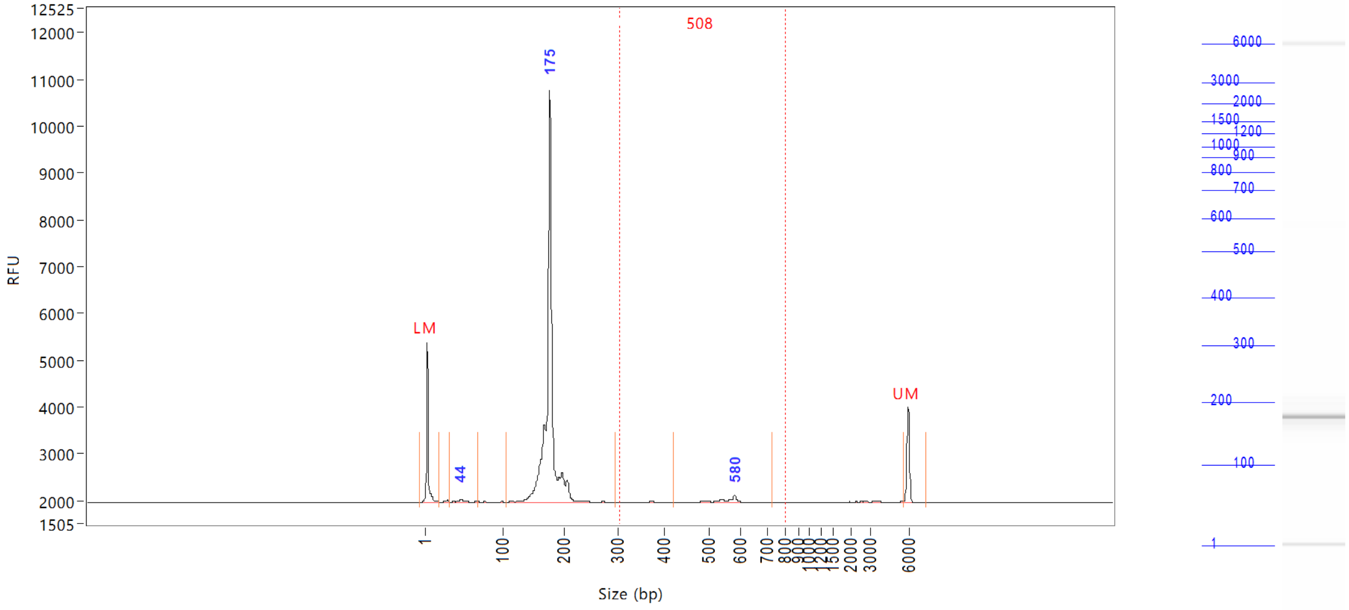

Peak	Size (bp)	Conc. (ng/uL)	From (bp)	To (bp)	Avg. Size (bp)	CV%	RFU	Corr. Peak Area
1	1 (LM)	0.0184	0	17	1	235.00	3407	20.563
2	44	0.0159	31	67	48	18.29	60	1.481
3	175	1.2286	105	294	175	8.40	8806	114.615
4	580	0.0353	422	725	549	9.98	164	3.294
5	6000 (UM)	0.0069	5679	7356	5980	2.25	2042	7.738

TIC:	1.2797	ng/uL
TIM:	12.220	nmole/L
Total Conc.:	1.3247	ng/uL

Smear Analysis 300 bp to 800 bp 0.0472 ng/ul 3.6 %Total 0.153 nmole/L 508 Avg. Size (b.p.) 19.24 %CV

```
Sample Peak Width (sec): 50   Sample Min Peak Height: 25    Sample Baseline V to V?: Y    Sample Baseline V to V pts: 3
Sample Filter: Binomial       # of Pts for Filter: 3        Sample Start Region (min): 0  Sample End Region (min): 50
Manual Baseline Start (min): 10     Manual Baseline End (min): 48
Marker Peak Width (sec): 5    Marker Min Peak Height: 200   Marker Baseline V to V?: Y    Marker Baseline V to V pts: 3
Lower Marker Selection: First Peak > 200 RFU                Upper Marker Selection: Last Peak > 200 RFU
Ladder Size (bp): 1, 100, 200, 300, 400, 500, 600, 700, 800, 900, 1000, 1200, 1500, 2000, 3000, 6000
Quantification Using: Ladder          Final Concentration (ng/uL): 0.0830         Dilution Factor: 12.0
```

 Copyright 2015 Advanced Analytical Technologies, Inc.

Sample: ID13507_89
Well Location: H5
Created: Tuesday, June 27, 2017 12:18:19 PM

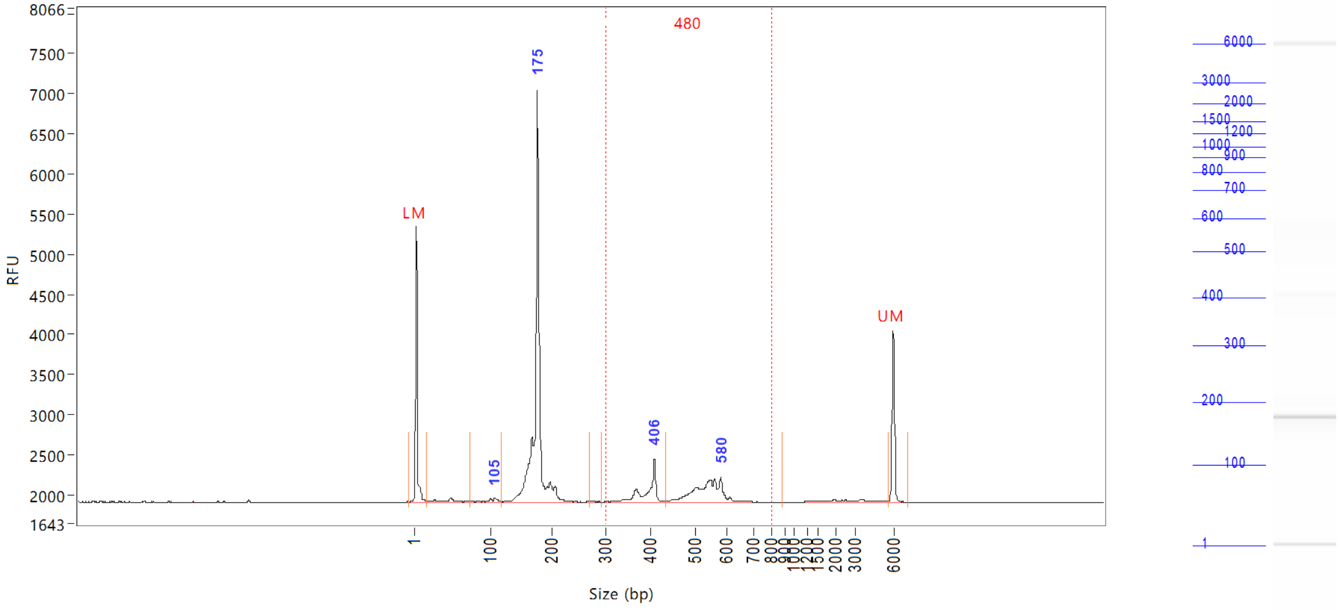

Peak	Size (bp)	Conc. (ng/uL)	From (bp)	To (bp)	Avg. Size (bp)	CV%	RFU	Corr. Peak Area
1	1 (LM)	0.0184	0	16	1	217.00	3431	20.333
2	105	0.0136	72	116	97	10.98	50	1.258
3	175	0.6133	116	269	173	7.96	5131	56.572
4	406	0.0886	291	431	384	6.69	541	8.177
5	580	0.1528	431	885	537	9.15	323	14.092
6	6000 (UM)	0.0074	5625	7117	5979	2.04	2137	8.201

TIC:	0.8683	ng/uL
TIM:	6.777	nmole/L
Total Conc.:	0.9122	ng/uL

Smear Analysis	300 bp to 800 bp	0.2406 ng/ul	26.4 %Total	0.825 nmole/L	480 Avg. Size (b.p.)	17.50 %CV

Sample Peak Width (sec): 50 Sample Min Peak Height: 25 Sample Baseline V to V?: Y Sample Baseline V to V pts: 3
Sample Filter: Binomial # of Pts for Filter: 3 Sample Start Region (min): 0 Sample End Region (min): 50
Manual Baseline Start (min): 10 Manual Baseline End (min): 48
Marker Peak Width (sec): 5 Marker Min Peak Height: 200 Marker Baseline V to V?: Y Marker Baseline V to V pts: 3
Lower Marker Selection: First Peak > 200 RFU Upper Marker Selection: Last Peak > 200 RFU
Ladder Size (bp): 1, 100, 200, 300, 400, 500, 600, 700, 800, 900, 1000, 1200, 1500, 2000, 3000, 6000
Quantification Using: Ladder Final Concentration (ng/uL): 0.0830 Dilution Factor: 12.0

 Copyright 2015 Advanced Analytical Technologies, Inc.

Sample: ID13507_90
Well Location: H6
Created: Tuesday, June 27, 2017 12:18:19 PM

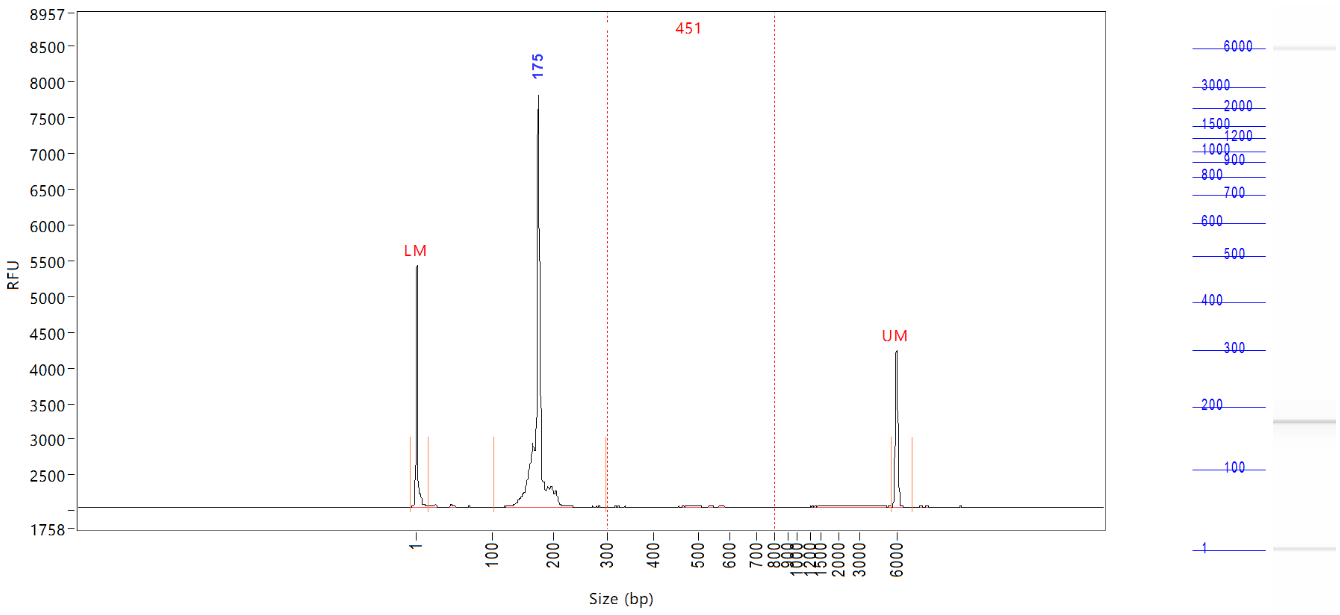

Peak	Size (bp)	Conc. (ng/uL)	From (bp)	To (bp)	Avg. Size (bp)	CV%	RFU	Corr. Peak Area
1	1 (LM)	0.0184	0	18	1	219.12	3375	20.141
2	175	0.7060	103	297	173	8.33	5753	64.513
3	6000 (UM)	0.0076	5572	7250	5974	1.93	2182	8.360

TIC:	0.7060	ng/uL
TIM:	6.643	nmole/L
Total Conc.:	0.7507	ng/uL

Smear Analysis 300 bp to 800 bp 0.0153 ng/ul 2.0 %Total 0.056 nmole/L 451 Avg. Size (b.p.) 18.53 %CV

Sample Peak Width (sec): 50 Sample Min Peak Height: 25 Sample Baseline V to V?: Y Sample Baseline V to V pts: 3
Sample Filter: Binomial # of Pts for Filter: 3 Sample Start Region (min): 0 Sample End Region (min): 50
Manual Baseline Start (min): 10 Manual Baseline End (min): 48
Marker Peak Width (sec): 5 Marker Min Peak Height: 200 Marker Baseline V to V?: Y Marker Baseline V to V pts: 3
Lower Marker Selection: First Peak > 200 RFU Upper Marker Selection: Last Peak > 200 RFU
Ladder Size (bp): 1, 100, 200, 300, 400, 500, 600, 700, 800, 900, 1000, 1200, 1500, 2000, 3000, 6000
Quantification Using: Ladder Final Concentration (ng/uL): 0.0830 Dilution Factor: 12.0

 Copyright 2015 Advanced Analytical Technologies, Inc.

Sample: ID13507_91
Well Location: H7
Created: Tuesday, June 27, 2017 12:18:19 PM

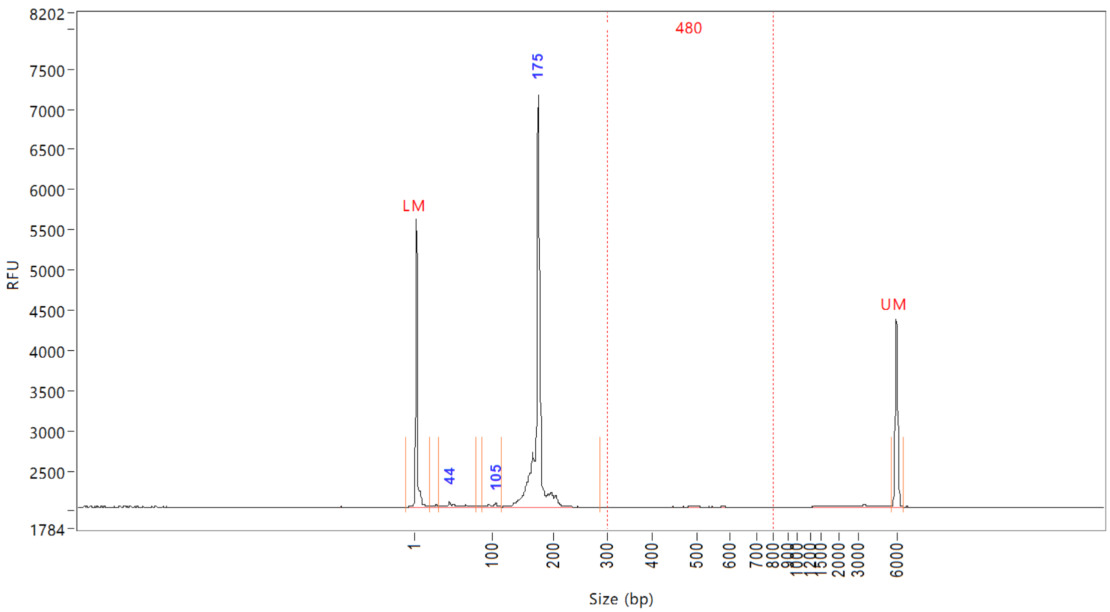

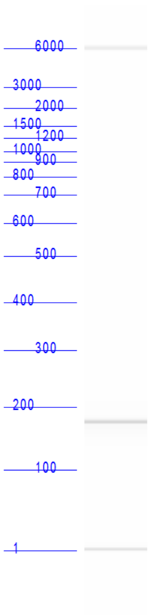

Peak	Size (bp)	Conc. (ng/uL)	From (bp)	To (bp)	Avg. Size (bp)	CV%	RFU	Corr. Peak Area
1	1 (LM)	0.0184	0	19	1	210.96	3591	21.074
2	44	0.0189	31	79	53	23.06	62	1.811
3	105	0.0099	87	116	99	6.92	49	0.947
4	175	0.5415	116	286	173	7.49	5126	51.772
5	6000 (UM)	0.0077	5652	6558	5981	1.66	2346	8.857

TIC:	0.5703	ng/uL
TIM:	5.946	nmole/L
Total Conc.:	0.5980	ng/uL

Smear Analysis 300 bp to 800 bp 0.0059 ng/ul 1.0 %Total 0.020 nmole/L 480 Avg. Size (b.p.) 15.97 %CV

```
Sample Peak Width (sec): 50   Sample Min Peak Height: 25    Sample Baseline V to V?: Y    Sample Baseline V to V pts: 3
Sample Filter: Binomial       # of Pts for Filter: 3        Sample Start Region (min): 0  Sample End Region (min): 50
Manual Baseline Start (min): 10     Manual Baseline End (min): 48
Marker Peak Width (sec): 5    Marker Min Peak Height: 200   Marker Baseline V to V?: Y    Marker Baseline V to V pts: 3
Lower Marker Selection: First Peak > 200 RFU                Upper Marker Selection: Last Peak > 200 RFU
Ladder Size (bp): 1, 100, 200, 300, 400, 500, 600, 700, 800, 900, 1000, 1200, 1500, 2000, 3000, 6000
Quantification Using: Ladder          Final Concentration (ng/uL): 0.0830          Dilution Factor: 12.0
```

 Copyright 2015 Advanced Analytical Technologies, Inc.

Sample: ID13507_92
Well Location: H8
Created: Tuesday, June 27, 2017 12:18:19 PM

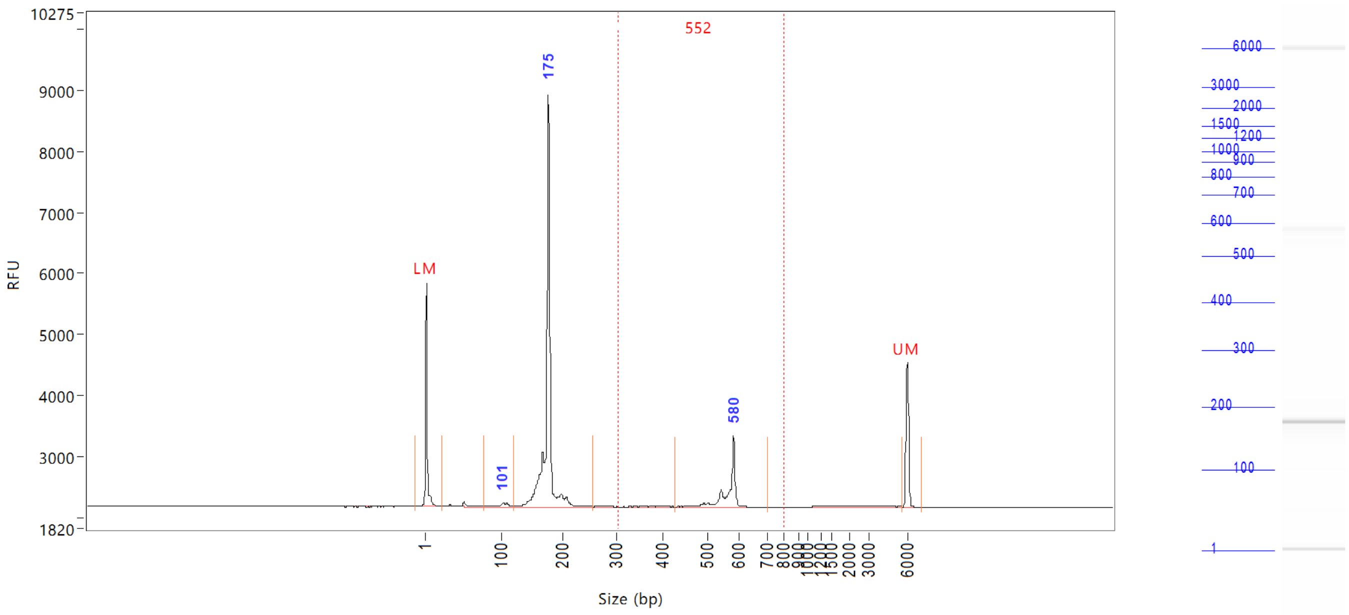

Peak	Size (bp)	Conc. (ng/uL)	From (bp)	To (bp)	Avg. Size (bp)	CV%	RFU	Corr. Peak Area
1	1 (LM)	0.0184	0	22	2	173.84	3652	22.063
2	101	0.0175	76	119	99	10.50	70	1.750
3	175	0.6453	119	255	173	6.97	6748	64.599
4	580	0.1181	425	703	558	5.91	1157	11.821
5	6000 (UM)	0.0075	5625	7117	5988	1.81	2381	9.024

TIC:	0.7809	ng/uL
TIM:	6.673	nmole/L
Total Conc.:	0.8261	ng/uL

Smear Analysis	300 bp to 800 bp	0.1224 ng/ul	14.8 %Total	0.365 nmole/L	552 Avg. Size (b.p.)	8.61 %CV

Sample Peak Width (sec): 50 Sample Min Peak Height: 25 Sample Baseline V to V?: Y Sample Baseline V to V pts: 3
Sample Filter: Binomial # of Pts for Filter: 3 Sample Start Region (min): 0 Sample End Region (min): 50
Manual Baseline Start (min): 10 Manual Baseline End (min): 48
Marker Peak Width (sec): 5 Marker Min Peak Height: 200 Marker Baseline V to V?: Y Marker Baseline V to V pts: 3
Lower Marker Selection: First Peak > 200 RFU Upper Marker Selection: Last Peak > 200 RFU
Ladder Size (bp): 1, 100, 200, 300, 400, 500, 600, 700, 800, 900, 1000, 1200, 1500, 2000, 3000, 6000
Quantification Using: Ladder Final Concentration (ng/uL): 0.0830 Dilution Factor: 12.0

 Copyright 2015 Advanced Analytical Technologies, Inc.

Sample: ID13507_93
Well Location: H9
Created: Tuesday, June 27, 2017 12:18:19 PM

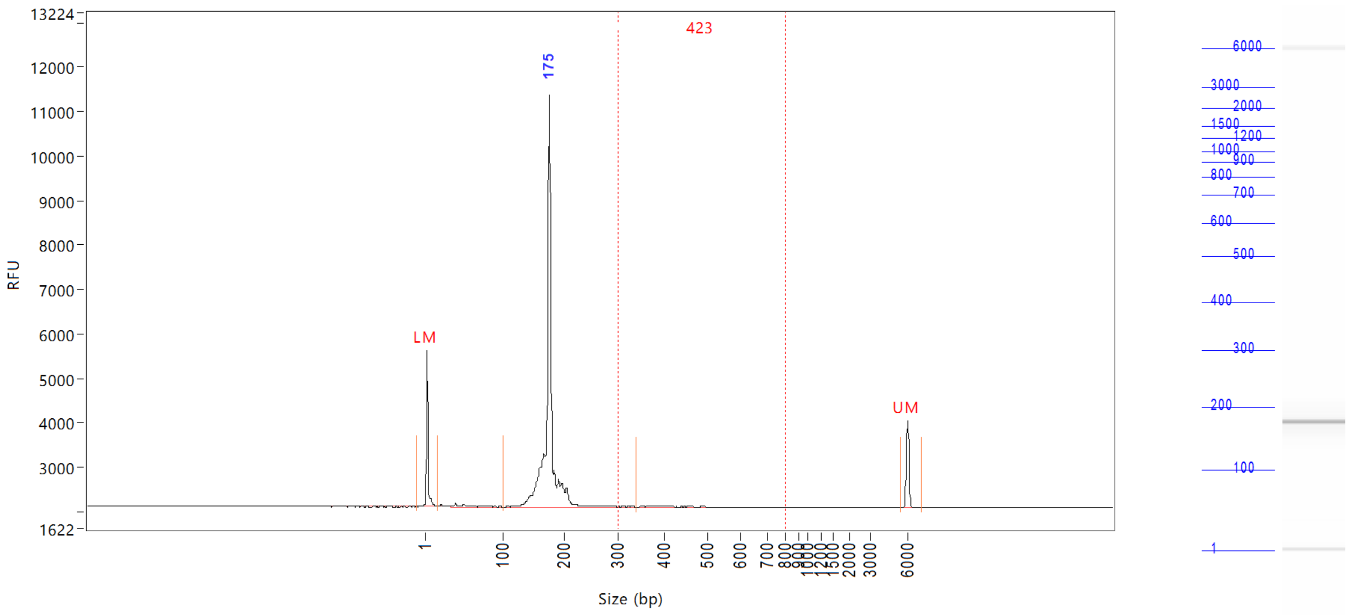

Peak	Size (bp)	Conc. (ng/uL)	From (bp)	To (bp)	Avg. Size (bp)	CV%	RFU	Corr. Peak Area
1	1 (LM)	0.0184	0	16	1	190.72	3509	20.779
2	175	1.1719	100	341	175	9.89	9262	110.485
3	6000 (UM)	0.0065	5491	7090	5984	1.78	1947	7.367

TIC:	1.1719	ng/uL
TIM:	11.028	nmole/L
Total Conc.:	1.2198	ng/uL

Smear Analysis 300 bp to 800 bp 0.0169 ng/ul 1.4 %Total 0.066 nmole/L 423 Avg. Size (b.p.) 19.59 %CV

```
Sample Peak Width (sec): 50   Sample Min Peak Height: 25    Sample Baseline V to V?: Y    Sample Baseline V to V pts: 3
Sample Filter: Binomial       # of Pts for Filter: 3        Sample Start Region (min): 0  Sample End Region (min): 50
Manual Baseline Start (min): 10     Manual Baseline End (min): 48
Marker Peak Width (sec): 5    Marker Min Peak Height: 200   Marker Baseline V to V?: Y    Marker Baseline V to V pts: 3
Lower Marker Selection: First Peak > 200 RFU                Upper Marker Selection: Last Peak > 200 RFU
Ladder Size (bp): 1, 100, 200, 300, 400, 500, 600, 700, 800, 900, 1000, 1200, 1500, 2000, 3000, 6000
Quantification Using: Ladder          Final Concentration (ng/uL): 0.0830         Dilution Factor: 12.0
```

 Copyright 2015 Advanced Analytical Technologies, Inc.

Supplementary Figure S3: Calibration curves and efficiency data for all the qPCR assays presented in this manuscript.

Candida albicans
Efficiency: 95.34%

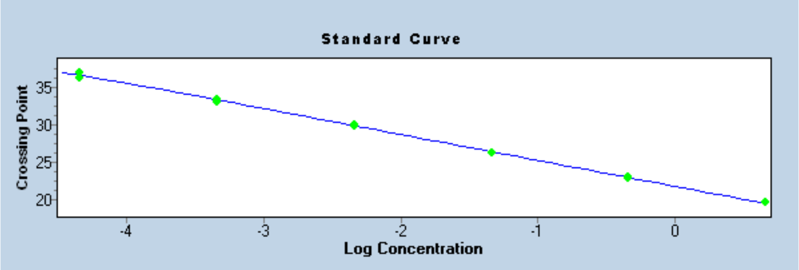

Candida tropicalis
Efficiency: 100.50%

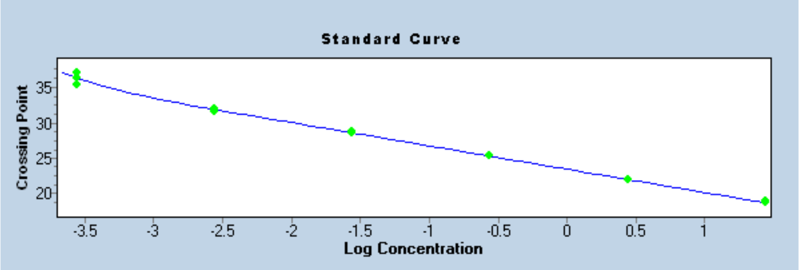

Saccharomyces cerevisiae
Efficiency: 105.96%

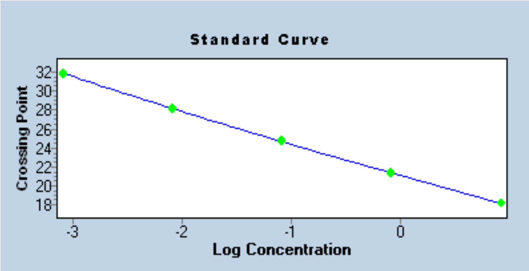

Cryptococcus neoformans
Efficiency: 101.44%

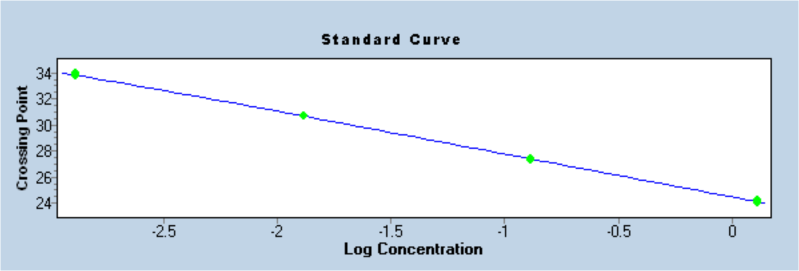

Malassezia furfur
Efficiency: 98.07%

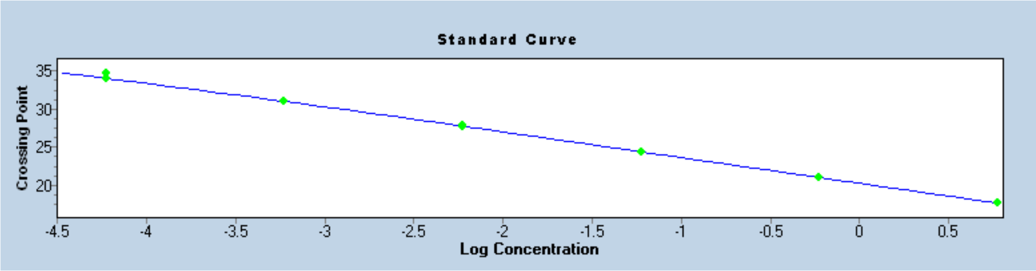

Aspergillus fumigatus
Efficiency: 99.87%

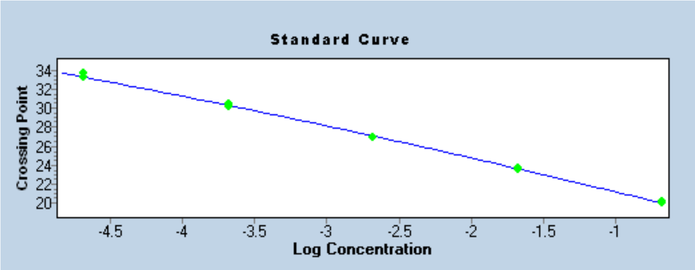

Penicillium chrysogenum
Efficiency: 94.10%

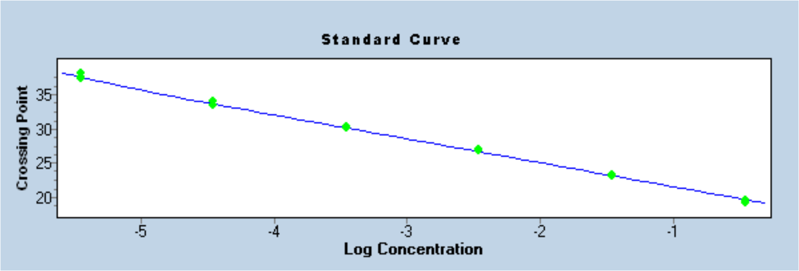

Figure 1D assay (*C. albicans* gBlock used as standard)
Efficiency: 95.91%

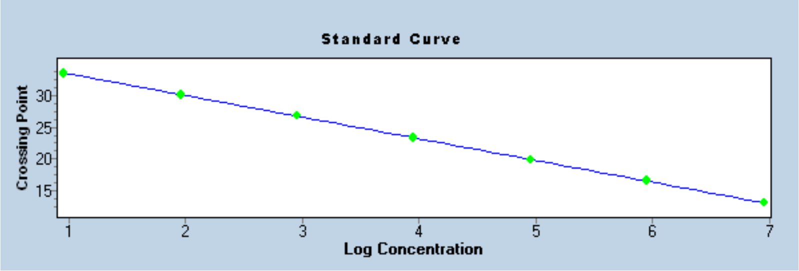

Figure 1E assays (*C. albicans* gBlock used as standard)
Efficiency: 98.27%

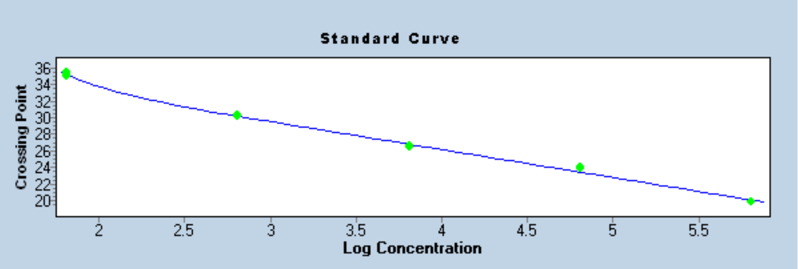

Supplementary Data S1: sequences to confirm id of the pure fungal strains used in this study.

```
>Af_ITS2_premix -- 10..338 of sequence (A. fumigatus)
TACCTGATCCGAGGTCACCTTAGAAAAATAAAGTTGGGTGTCGGCTGGCGCCGGCCGGGC
CTACAGAGCAGGTGACAAAGCCCCATACGCTCGAGGACCGGACGCGGTGCCGCCGCTGCC
TTTCGGGCCCGTCCCCCGGGAGAGGGGGACGGGGCCCAACACACAAGCCGTGCTTGAGG
GCAGCAATGACGCTCGGACAGGCATGCCCCCCGGAATACCAGGGGGCGCAATGTGCGTTC
AAAGACTCGATGATTCACTGAATTCTGCAATTCACATTACTTATCGCATTTCGCCGCGTT
CTTCATCGCTGCGAGTCTAGAATGAAATT
>Ca_ITS2_premix -- 12..320 of sequence (C. albicans)
TCTACCTGATTTGAGGTCAAGTTTGAAGATATACGTGGTAGACGTTACCGCCGCAAGCAA
TGTTTTTGGTTAGACCTAAGCCATTGTCAAAGCGATCCCGCCTTACCACTACCGTCTTTC
AAGCAAACCCAAGTCGTATTGCTCAACACCAAACCCAGCGGTTTGAGGGAGAAACGACGC
TCAAACAGGCATGCCCTCCGGAATACCAGAGGGCGCAATGTGCGTTCAAAGATTCGATGA
TTCACGAATACCTGCAATTCATATTACGTATCGCATTTCGCCGCGTTCTTCATCGCTGAC
GAGTCTAGA
>Cn_ITS2_premix -- 10..352 of sequence (C. neoformans)
CTACCTGATTTGAGGTCAACAAAAAGAGATGGTTGTTATCAGCAAGCCGAAGACTACCCC
ATAGGCCCAGCGAAACTTATTACGCCGGGCTGACAGGTAATCACCTTCCCACTAACACAT
TTAAGGCGAGCCGACGTCCTTTGCAGGTCGCGGCAAACACCCAAATCCAAGTCCAACAGG
TAATAAAACCCGAGGGATTGAGATTTTCATGACTCTCAAACAGGCATGCCCTTCGGAATA
CCAAAGGGCGCAAGTTGCGTTCAAAGATTCGATGATTCACTGAATTCTGCAATTCACATT
ACTTATCGCATTTCGCCGCGTTCTTCATCGCTGCGAGTCTAGA
>Ct_ITS2_premix -- 11..302 of sequence (C. tropicalis)
TCTACCTGATTTGAGGTCAAGTTATGAAATAAATTGTGGTGGCCACTAGCAAAATAAGCG
TTTTTGGATAAACCTAAGTCGCTTAAAATAAGTTTCCACGTTAAATTCTTTCAAACAAAC
CTAGCGTATTGCTCAACACCAAACCCGGGGGTTTGAGGGAGAAATGACGCTCAAACAGGC
ATGCCCTTTGGAATACCAAAGGGCGCAATGTGCGTTCAAAGATTCGATGATTCACGAATA
TCTGCAATTCATATTACGTATCGCATTTCGCCGCGTTCTTCATCGCTACGGA
>Mf_ITS2_premix -- 11..528 of sequence (M. furfur)
TCTACCTGATTTGAGACCAGAAATGAAAAAAGGGAATGCGTTCACAAGAACTGCTCCATG
CTTGCGCACAGACCAGCCTCCACGCAGGATTGGTAGCGCACCAACACACCCACAGAGTGA
CAAAGGCACCACATCGGCCCCTTGCCTCAGGACGCACACACAGCAAATGACGTATCATGC
CATGCGCTTGCCTGGGGCAAAGTTTGGTATAGCTGACGGGCGGAGTTCCTCCATCCCCTC
TGCCGCCCTCCCTTTCAGAGCGGTTTGCAGAGTGTGTCCCAAAGGCGCTAATGCATTTCG
GGCGAGCCTGTTTCTTGCGAAACAGGCAGAAGCACCCATCCAACCTCGTCCGCCAAAGCA
GTGCAATCGCAACCGCTTGGGGAGAGAGAATTCACGGCACTCAAACAGGCATGCTCCACG
GAATACCATGGAGCGCAAGGTGCGTTCAAAGATTCGATGATTCACGGAATTCTGCAATTC
ACATTACCTATCGCGTTTCGCCGCGTTCTTCATCGCTG
>Pc_ITS2_premix -- 9..321 of sequence (P. crysogenum)
CTACCTGATCCGAGGTCACCTGGATAAAAATTTGGGTTGATCGGCAAGCGCCGGCCGGGC
CTACAGAGCGGGTGACAAAGCCCCATACGCTCGAGGACCGGACGCGGTGCCGCCGCTGCC
TTTCGGGCCCGTCCCCCGGGATCGGAGGACGGGGCCCAACACACAAGCCGTGCTTGAGGG
CAGCAATGACGCTCGGACAGGCATGCCCCCCGGAATACCAGGGGGCGCAATGTGCGTTCA
AAGACTCGATGATTCACTGAATTTGCAATTCACATTACGTATCGCATTTCGCCGCGTTCT
TCATCGCTGAGGA
>Sc_ITS2_premix -- 9..193 of sequence (S. cerevisiae)
TCTACCTGATTTGAGGTCAACTTTAAGAACATTGTTCGCCTAGACGCTCTCTTCTTATCG
ATAACGTTCCAATACGCTCAGTATAAAAAAGATTAGCCGCAGTTGGTAAAACCTAAAACG
ACCGTACTTGCATTATACCTCAAGCACGCAAAGAAACCTCTCTTTGGAAAAAAAAAATCC
CAAGG
```
